# New 1,3,4-Thiadiazole Derivatives with Anticancer Activity

**DOI:** 10.3390/molecules27061814

**Published:** 2022-03-10

**Authors:** Sara Janowska, Dmytro Khylyuk, Anna Bielawska, Anna Szymanowska, Agnieszka Gornowicz, Krzysztof Bielawski, Jarosław Noworól, Sławomir Mandziuk, Monika Wujec

**Affiliations:** 1 Department of Organic Chemistry, Faculty of Pharmacy, Medical University, 4a Chodzki Street, 20-093 Lublin, Poland; sarajanowska@umlub.pl (S.J.); dmytrokhylyuk@umlub.pl (D.K.); 2 Department of Biotechnology, Faculty of Pharmacy, Medical University of Bialystok, Kilinskiego 1 Street, 15-089 Bialystok, Poland; anna.bielawska@umb.edu.pl (A.B.); anna.szymanowska@umb.edu.pl (A.S.); agnieszka.gornowicz@umb.edu.pl (A.G.); 3 Department of Synthesis and Technology of Drugs, Faculty of Pharmacy, Medical University of Bialystok, Kilinskiego 1 Street, 15-089 Bialystok, Poland; krzysztof.bielawski@umb.edu.pl; 4 Health Care Institute, State Higher School of Technology and Economics in Jarosław, Czarnieckiego 16 Street, 37-500 Jarosław, Poland; jaroslaw.noworol@pwste.edu.pl; 5 Department of Pneumology, Oncology and Allergology, Medical University of Lublin, 8 Jaczewskiego Street, 20-090 Lublin, Poland; slawomir.mandziuk@umlub.pl

**Keywords:** 1,3,4-thiadiazole, cytotoxic activity, breast cancer, docking studies

## Abstract

We designed and synthesized the 1,3,4-thiadiazole derivatives differing in the structure of the substituents in C2 and C5 positions. The cytotoxic activity of the obtained compounds was then determined in biological studies using MCF-7 and MDA-MB-231 breast cancer cells and normal cell line (fibroblasts). The results showed that in both breast cancer cell lines, the strongest anti-proliferative activity was exerted by 2-(2-trifluorometylophenylamino)-5-(3-methoxyphenyl)-1,3,4-thiadiazole. The IC_50_ values of this compound against MCF-7 and MDA-MB-231 breast cancer cells were 49.6 µM and 53.4 µM, respectively. Importantly, all new compounds had weaker cytotoxic activity on normal cell line than on breast cancer cell lines. In silico studies demonstrated a possible multitarget mode of action for the synthesized compounds. The most likely mechanism of action for the new compounds is connected with the activities of Caspase 3 and Caspase 8 and activation of BAX proteins.

## 1. Introduction

Breast cancer affects 14% of all women living in the world [1]. It is the most frequently diagnosed neoplasm in female patients. The basic available treatments for breast cancer are surgery, radiotherapy, and chemotherapy, individually tailored to the patient. Unfortunately, one of the main problems of the pharmacotherapy of cancers, including breast cancer, is the rapidly developing drug resistance. For this reason, it is necessary to search for new anticancer drugs [2].

Estrogens are listed by the WHO as one of the most important factors stimulating the development of breast cancer. These hormones can promote the development of breast cancer by stimulating proliferation and altering gene expression. Estrogens can also initiate the process of carcinogenesis through reactive metabolites. Many of the pharmacological treatments for breast cancer currently available, such as tamoxifen, mainly target the estrogen receptors (ER). As a consequence, these drugs are not effective in the treatment of non-estrogen-dependent cases of breast cancer. Research is ongoing to find new therapeutic and chemopreventive agents acting independently of estrogen receptors, effective against both estrogen-dependent and non-estrogen-dependent breast cancer cells [3].

Over the past 20 years, tremendous efforts have been made to unravel the molecular basis of cancer. Several proteins involved in the development of various types of cancer have been described, such as the suppressor protein p53 or the protein S100A4, a metastasis promoter [4]. This newly gained knowledge about the processes in the neoplastic cell is used to design and synthesize new cytotoxic and cytostatic substances. This resulted in the significant development of cancer pharmacotherapy towards targeted therapies. New drugs are designed to target tumor-specific proteins. Such therapeutics tend to have fewer severe side effects than old drugs because they are more selective for neoplastic changes [5]. One of them with documented antitumor activity are thiadiazoles. Four isomeric forms can be distinguished among the thiadiazole derivatives: 1,3,4-thiadiazole; 1,2,3-thiadiazole; 1,2,4-thiadiazole; and 1,2,5-thiadiazole. Published studies show that 1,3,4-thiadiazole derivatives are the most promising group in terms of potential therapeutic activity [6]. Compounds belonging to the group of 1,3,4-thiadiazole derivatives have shown in studies the potential of antibacterial, antifungal, antituberculosis, anti-inflammatory, analgesic, antipsychotic, anticonvulsant, antidepressant, and anti-leishmanial properties. Many studies have been published showing the anticancer activity of substances from this group [7].

It has been shown that some 1,3,4-thiadiazole derivatives can interfere with processes related to DNA replication. The bioactive properties of these compounds are explained by the fact that their molecular structure contains a heterocyclic ring, which is a bioisostere of a pyrimidine, which is the backbone of the structures of three nucleobases. Probably due to this similarity, cytostatic active 1,3,4-thiadiazole molecules interfere with DNA synthesis and, consequently, inhibit replication of both human tumor and bacterial cells. This allows them to inhibit the multiplication of both bacterial and cancer cells. Therefore, among the 1,3,4-thiadiazole derivatives, candidates for new antibiotic and anticancer drugs are sought [6].

**Figure 1:**
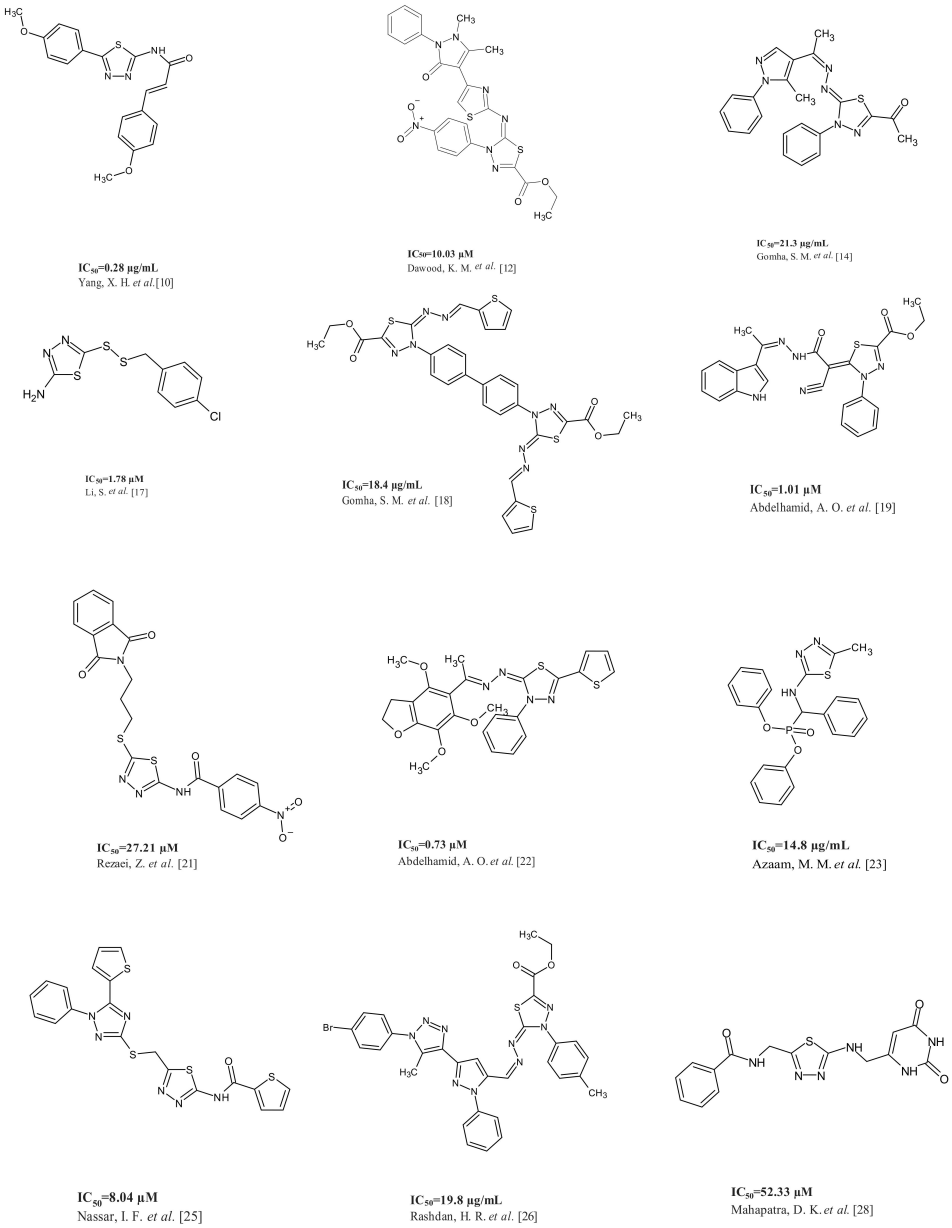
1,3,4-thiadiazole derivatives recently identified as possessing cytotoxic activity against MCF-7 cancer cell lines [10,12,14,17,18,19,21,22,23,25,26,28].

Among the 1,3,4-thiadiazole derivatives tested for antitumor activity, there are thiadiazole systems fused with other rings and simple 2,5-disubstituted rings. Significantly more published research results indicate the therapeutic potential of molecules containing a simple unfused ring. Some of them have been shown to have promising anticancer activity, exceeding the reference drugs in tests. Many studies have demonstrated the activity of compounds from this group against breast cancer cell lines (Figure 1) [8,9,10,11,12,13,14,15,16,17,18,19,20,21,22,23,24,25,26,27,28,29]. Therefore, we assumed that the search for cytotoxic compounds in the group of simple 2,5-disubstituted 1,3,4-thiadiazole derivatives is a promising direction of research.

Thiadiazole derivatives act through various molecular targets, for example, CA IX carbonic anhydrase, Src and Abl kinases, and topoisomerase II [30,31,32].

In order to develop new 1,3,4-thiadiazole derivatives, we created a library of molecular patterns and reports on the antitumor activity demonstrated by them in tests. Based on the analysis of the collected data, we designed and synthesized a group of 1,3,4-thiadiazole derivatives differing in the structure of the substituents in C2 and C5 positions. The cytotoxic activity of the obtained compounds was then determined in biological studies using MCF-7 and MDA-MB-231 breast cancer cells. The MCF-7 cell line, derived from a pleural effusion of malignant breast cancer, is a widely studied model for hormone-dependent human breast cancer. In contrast, the MDA-MB-231 cell line provides a model for human breast cancer, which exhibits an estrogen-independent state and does not express estrogen receptors.

In addition, in silico studies, we determined the interaction of the obtained compounds with proteins involved in neoplastic processes, such as topoisomerase IIb, Caspase 3, Caspase 8, Bcl-xl, Bcl2, and BAX.

## 2. Results and Discussion

### 2.1. Chemistry

**Scheme 1:**
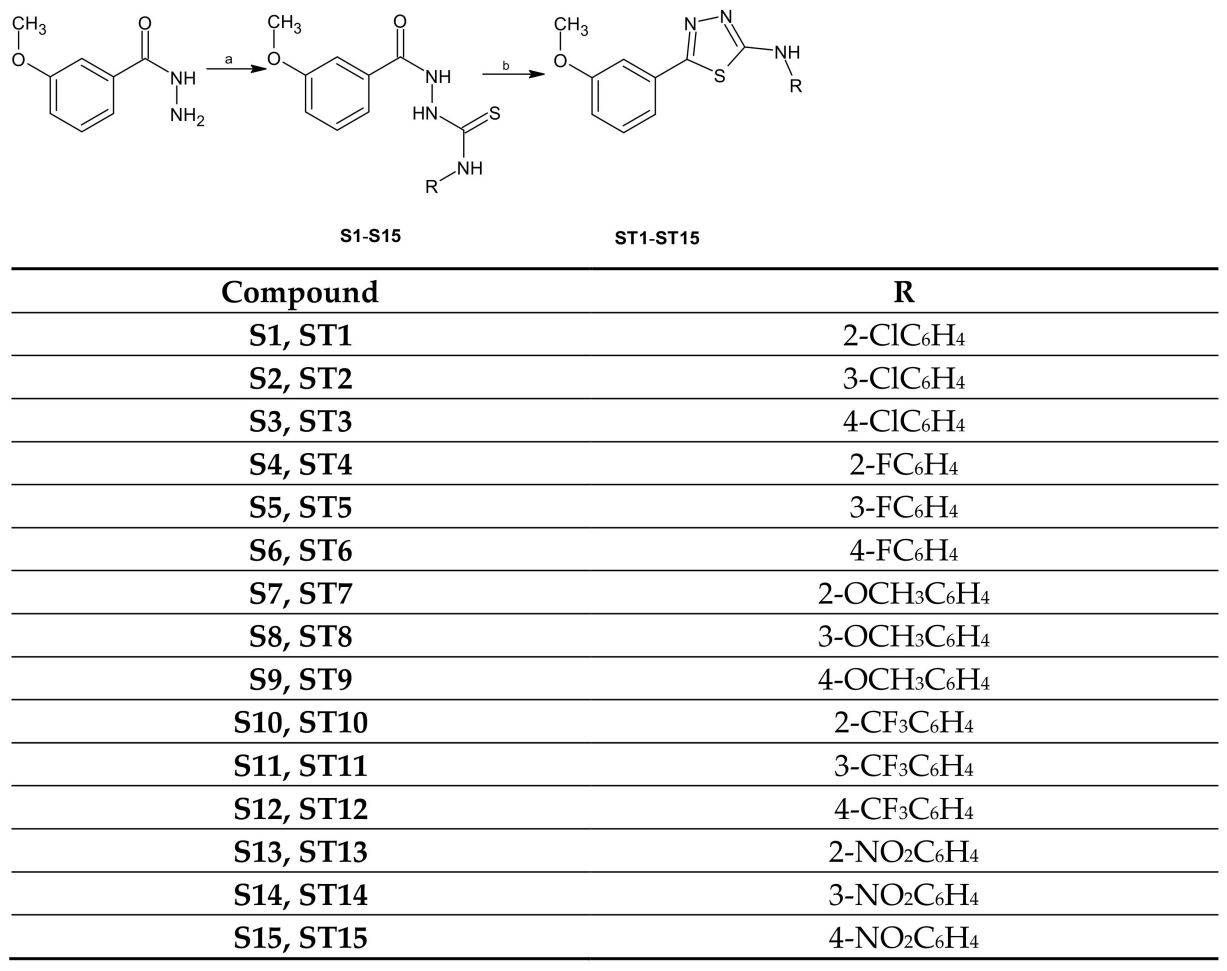
Synthesis of thiosemicarbazide and 1,3,4-thiadiazole derivatives. Reagents and conditions: (**a**) aryl isothiocyanates, 96% EtOH, reflux; (**b**) H_2_SO_4_ (conc), rt.

The title compounds **ST1–ST15** were obtained in a two-step synthesis (Scheme 1).

The reaction of 3-methoxybenzhydrazide with aryl isothiocyanates (2-, 3-, 4-chlorophenyl, 2-, 3-, 4-fluorophenyl, 2-, 3-, 4-methoxyphenyl, 2-, 3-, 4-trifluoromethylphenyl and 2-, 3-, 4-nitrophenyl) gave the corresponding 1,4-disubstituted thiosemicarbazides **S1**–**S15**, which in the reaction with concentrate sulfuric acid in room temperature lead to formation 1,3,4-thiadiazole derivatives **ST1**–**ST15**. The reaction yields ranged from 43 to 96% for thiosemicarbazide derivatives and 18–70% for 1,3,4-thiadiazoles. The structures of the new compounds were determined using IR, ^1^H NMR, ^13^C NMR, and elemental analyses.

The ^1^H NMR spectra showed the chemical shifts of the N1, N2, and N4 protons, which confirmed the formation of the thiosemicarbazide scaffold. The thiosemicarbazide protons were observed between 9.22 and 10.08, between 9.72 and 10.08, and between 10.49 and 10.73 ppm, respectively, as two or three singlets. The ^1^H NMR spectra confirmed the formation of thiadiazole derivatives. The N1, N2, and N4 protons of the thiosemicarbazide were not detected in the 1,3,4-thiadiazole compounds. Instead, N-H peaks of the amino group of cyclic compounds were observed between 8.26 and 11.32 ppm. Infrared spectra showed strong peaks in the range 1651–1688 cm^−1^ corresponding to the carbonyl group and 1316–1368 cm^−1^, which corresponds to the C=S group in thiosemicarbazide derivatives. In IR spectra of thiadiazole derivatives, absorption peaks in the range of 715–781 cm^−1^ from C-S were observed. In the ^13^C NMR spectra, the signals of a methyl group in the range of 53.13–57.40 ppm were observed. All other signals of carbons are adequate to the structure. The ^1^H NMR and ^13^C NMR spectra are presented in the Supplementary Materials file.

### 2.2. Bioactivity Studies

**Figure 2:**
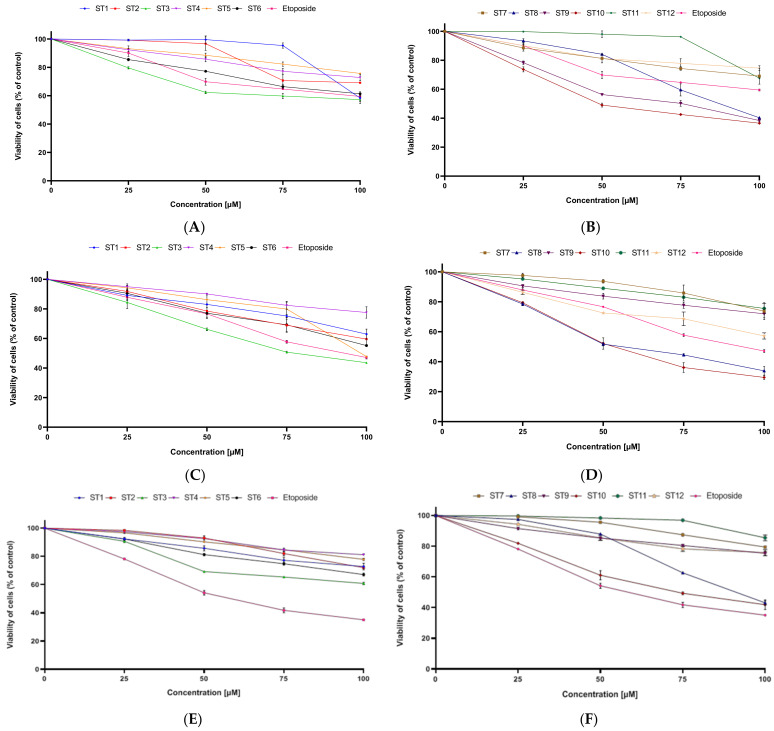
The viability of MCF-7 (**A**,**B**) and MDA-MB-231 (**C**,**D**) cells and fibroblasts (**E**,**F**) treated for 24 h with various concentrations of tested compounds (**ST1–ST12**). Mean ± SD values from three independent experiments (n = 3) done in duplicate are presented.

The cytotoxic activity of novel synthesized compounds (**ST1**–**ST15**) against two breast cancer cell lines was assessed using MTT assay and biosynthesis DNA. The cells were exposed to the tested compounds for 24 h. As a reference drug, etoposide was used. The obtained results revealed that all tested compounds exhibited cytotoxicity in concentration-dependent manner against MCF-7 and MDA-MB-231 (Figure 2).

The strongest anticancer activity against MCF-7 was exhibited by **ST10**, which had IC_50_ = 49.6 µM. A moderate inhibitory effect on the survival of MCF-7 cells was demonstrated by two compounds, **ST8** and **ST9**, which had IC_50_ = 87.4 µM and 75.1 µM, correspondingly. It is worth noticing that those three compounds exerted higher anticancer activity against MCF-7 than etoposide. The IC_50_ of the reference drug and the other synthesized compounds was above 100 µM.

In the MDA-MB-231, **ST10** also compound exerted the strongest anticancer activity. The concentration of **ST10** needed to inhibit 50% viability of the cells was 53.4 µM. Similar anticancer activity had compound **ST8** (IC_50_ = 56.4 µM). The moderate anticancer activity against this cell line was exhibited by **ST3**. The IC_50_ value was 78.6 µM. Other synthesized compounds had IC_50_ above 100 µM.

The results presented in this paper indicate that in both cell lines, the strongest cytotoxic properties were exhibited by compound **ST10**. However, high anticancer activity against MDA-MB-231 was also exerted by **ST8**. The biological activity of obtained thiadiazoles (**ST1**–**ST15**) depends on the position and kind of the substituent of N.

A significant problem with many anticancer compounds is their high toxicity to normal cells. Therefore, the effect of novel 1,3,4-thiadiazole derivatives on the viability of normal cell lines (fibroblasts) was investigated. It was shown that all new compounds had weaker cytotoxic activity on normal cell lines than on breast cancer cell lines.

**Figure 3:**
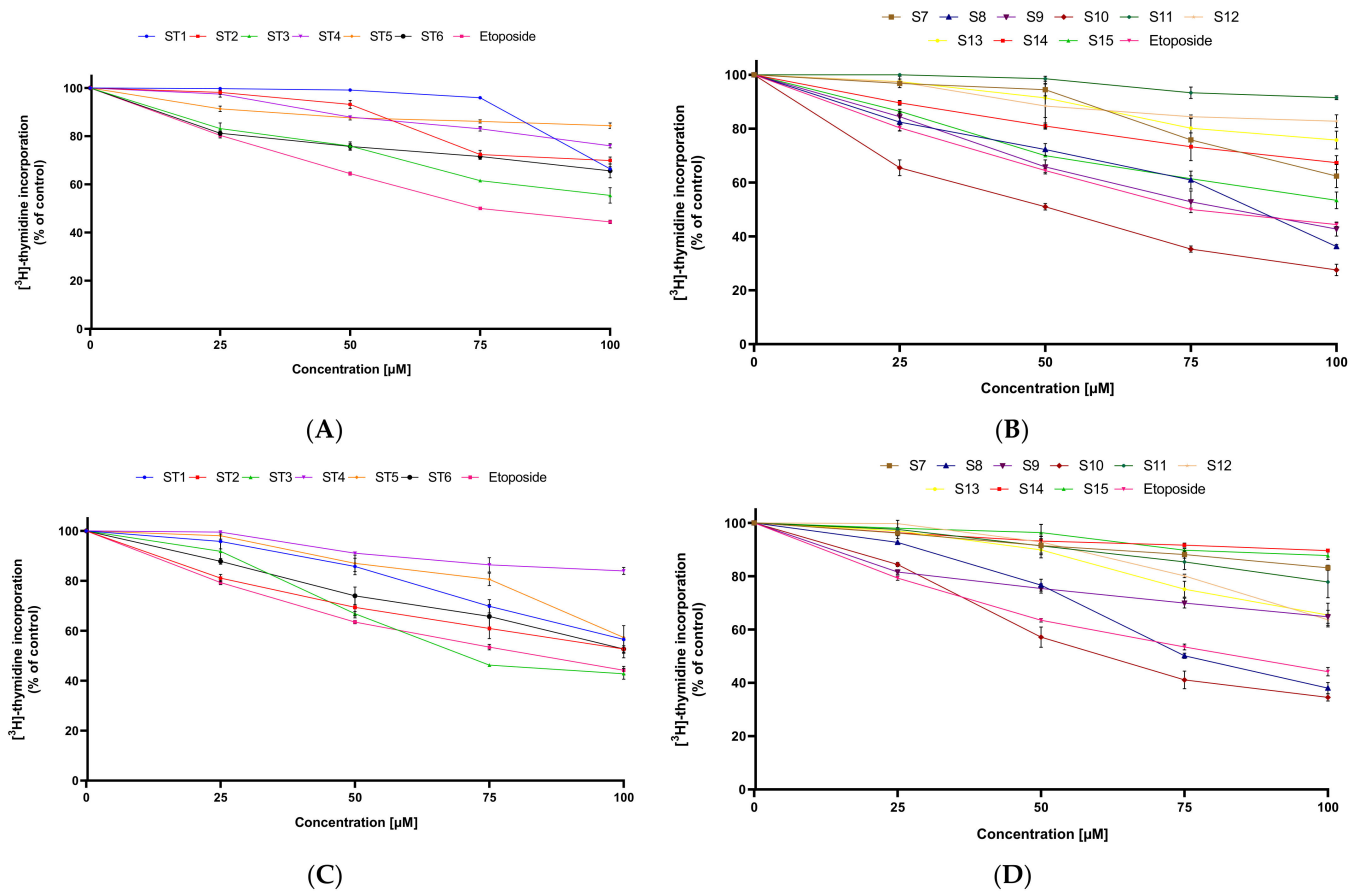
The anti-proliferative effect of the tested compounds (**ST1**–**ST15**) against MCF-7 (**A**,**B**) and MDA-MB-231 (**C**,**D**). Mean ± SD values from three independent experiments (*n* = 3) done in duplicate are presented.

Further research indicated that all tested compounds (**ST1**–**ST15**) lead to inhibition of DNA biosynthesis in MCF-7 and MDA-MB-231, and as the used concentration increased, the effect was enhanced (Figure 3).

In the [3H]-thymidine incorporation assay, **ST10** exhibited the strongest anti-proliferative activity against MCF-7 (IC_50_ = 51.5 µM), followed by **ST9** (IC_50_ = 82 µM) and **ST8** (IC_50_ = 86.5 µM). The rest of the synthesized compounds showed lower inhibitory activity against MCF-7 than the reference drug.

In MDA-MB-231, **ST10** compound also exerted the strongest anti-proliferative activity. The IC_50_ value of **ST10** against this cell line was 64.2 µM. Moreover, two compounds, **ST3** and **ST8**, inhibited proliferation of this cell line higher than the reference drug (etoposide). The IC_50_ values were 73.8 µM and 75.2 µM, respectively. The other derivatives of 1,3,4-thiadiazole had weak anti-proliferative activity against the MDA-MB-231 breast cancer cell line. The IC_50_ value for each of them was above 100 µM.

These results showed that in both breast cancer cell lines, the strongest anti-proliferative activity was exerted by compound **ST10**. The moderate cytostatic activity against estrogen-dependent cell lines (MCF-7) had compounds **ST8** and **ST9**, while against estrogen-independent cell line (MDA-MB-231), mild anti-proliferative activity had compound **ST3** and compound **ST8**.

### 2.3. Docking

The docking simulations technique was performed using AutoDock Tools with 15 compounds. Each compound was docked into each of six different targets. The result of the docking study presented as binding energies and Estimated Inhibition Constant, Ki. From a total of 50 docking modes represented by LGA cluster analysis, the lowest energy docking mode with respective Ki prediction was selected from each docking simulation. For estimating inhibition activity to each target, we compared the binging energies of synthesized compounds and reference molecules from the downloaded spectrum (exception –BAX protein). All results are summarized in Table 1 and Table 2. Docking simulations demonstrated a possible multitarget mode of action for the synthesized compounds. Nevertheless, anticancer properties of **ST1**-**ST15** probably are mainly connected with the activities of Caspase 3 and Caspase 8 and activation of BAX proteins. The most active compounds according to biological assays **ST8** and **ST15** also demonstrated good results during in silico simulations, which are closed to docking scores of the references ligands.

**Figure 4:**
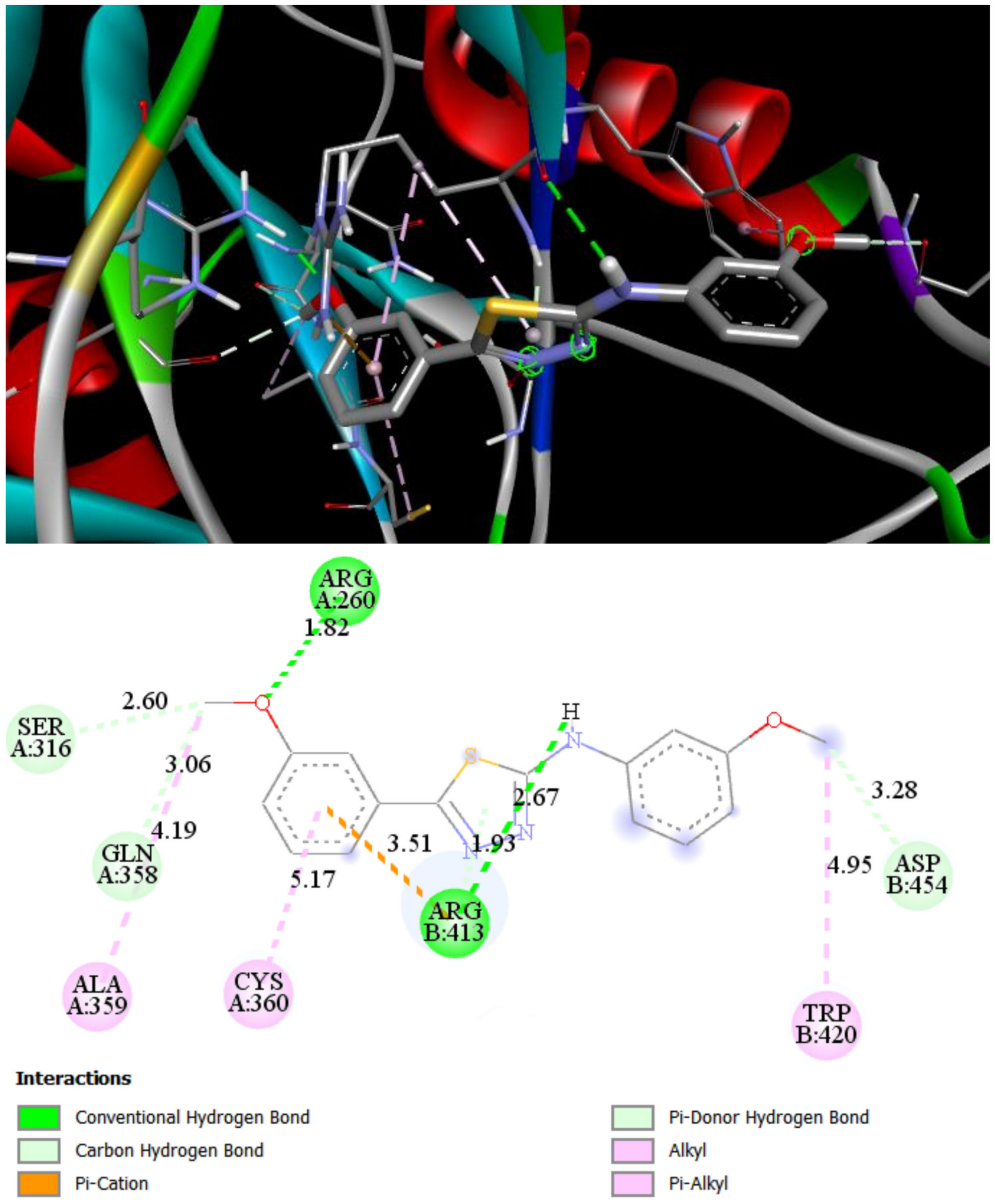
2D and 3D interaction maps of **ST8** with Caspase 8.

**ST8** makes two hydrogen bonds with ARG260 (1.82 Å) and ARG413 (2.67 Å). Additionally, lipophilic amino acids ALA359, CYS360, and TRP410 are bound to the ligands by alkyl and Pi-alkyl interactions. Additionally, weak carbon–hydrogen bonds with SER316 and GLN358 increase the summary binding energy of **ST8** with Caspase 8 (Figure 4).

**Figure 5:**
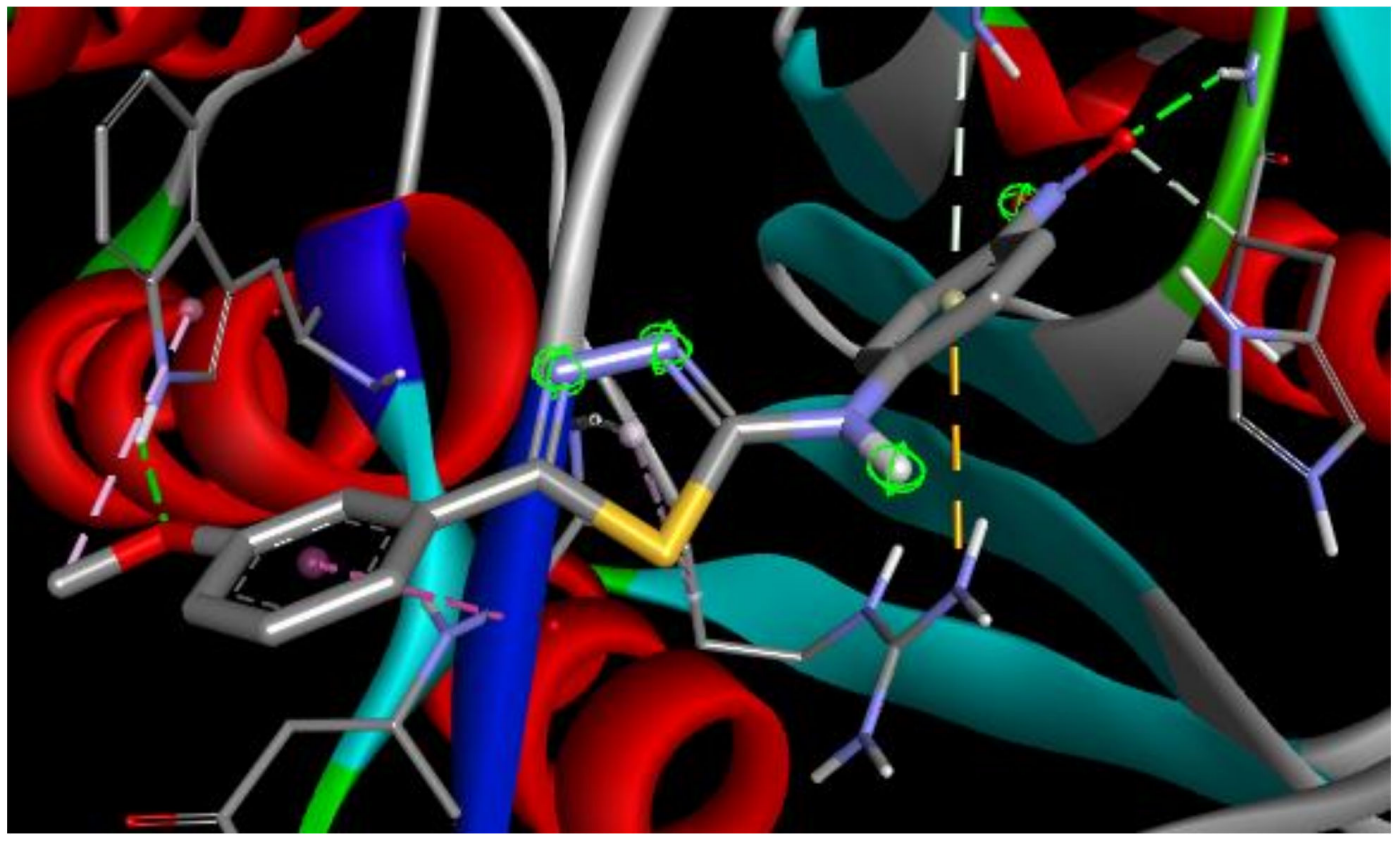

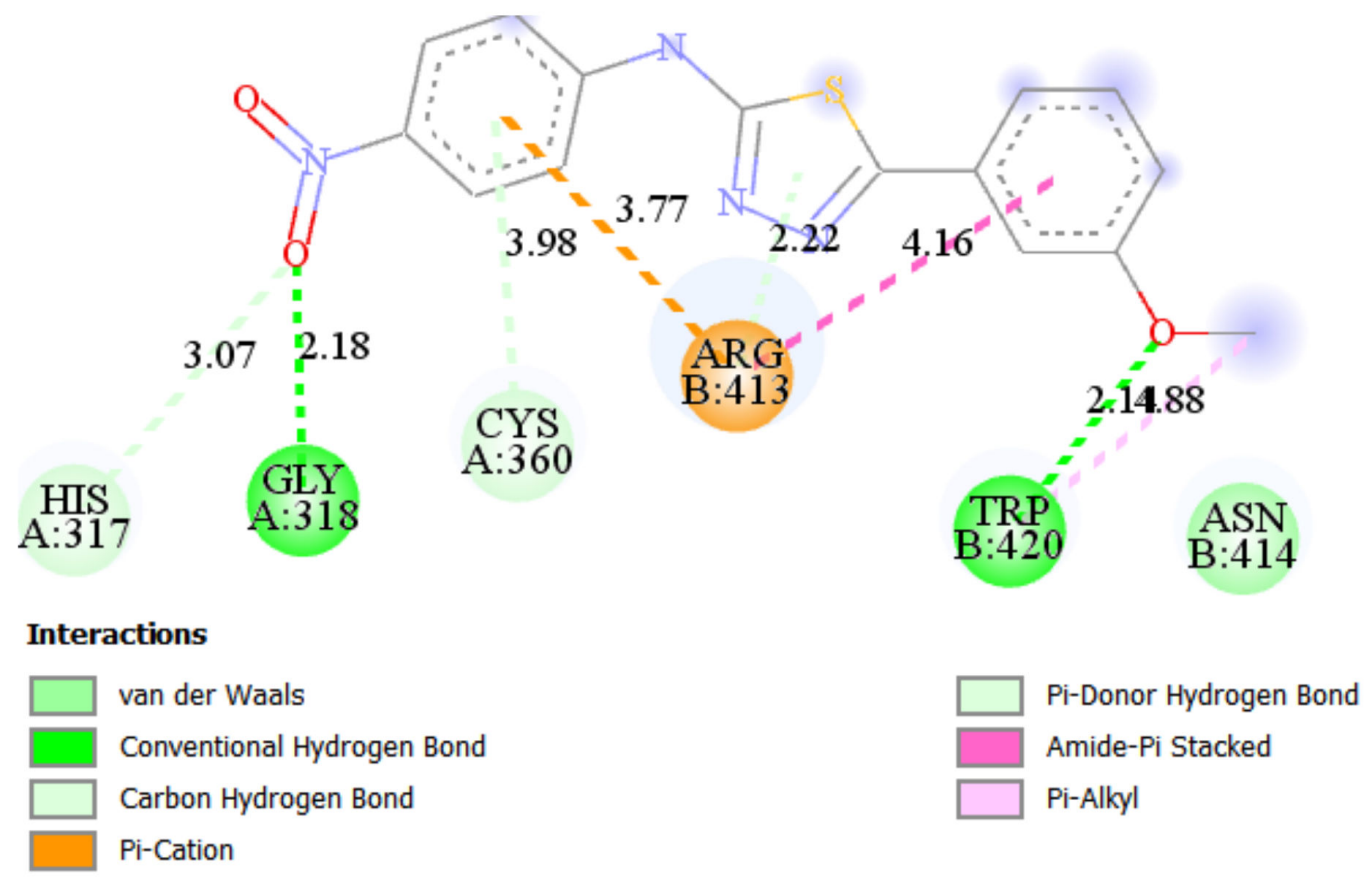
2D and 3D interaction maps of **ST15** with Caspase 8.

The best docking score in simulation with Caspase 8 was achieved by **ST15** with a value of binding energy −8.28 kcal/mol and inhibition constant 0.852 μM. Compound **ST15** bound to the active site by its oxygen of 4-NO_2_ with GLY318 as a hydrogen bond acceptor with a bond length 2.18 Å. 3-methoxy group forms the hydrogen bond to the TRP420 with a bond length of 2.14 Å. ARG413 forms with both phenyl rings of the molecules by Pi-cation and amide-Pi stacked interactions (Figure 5).

**Figure 6:**
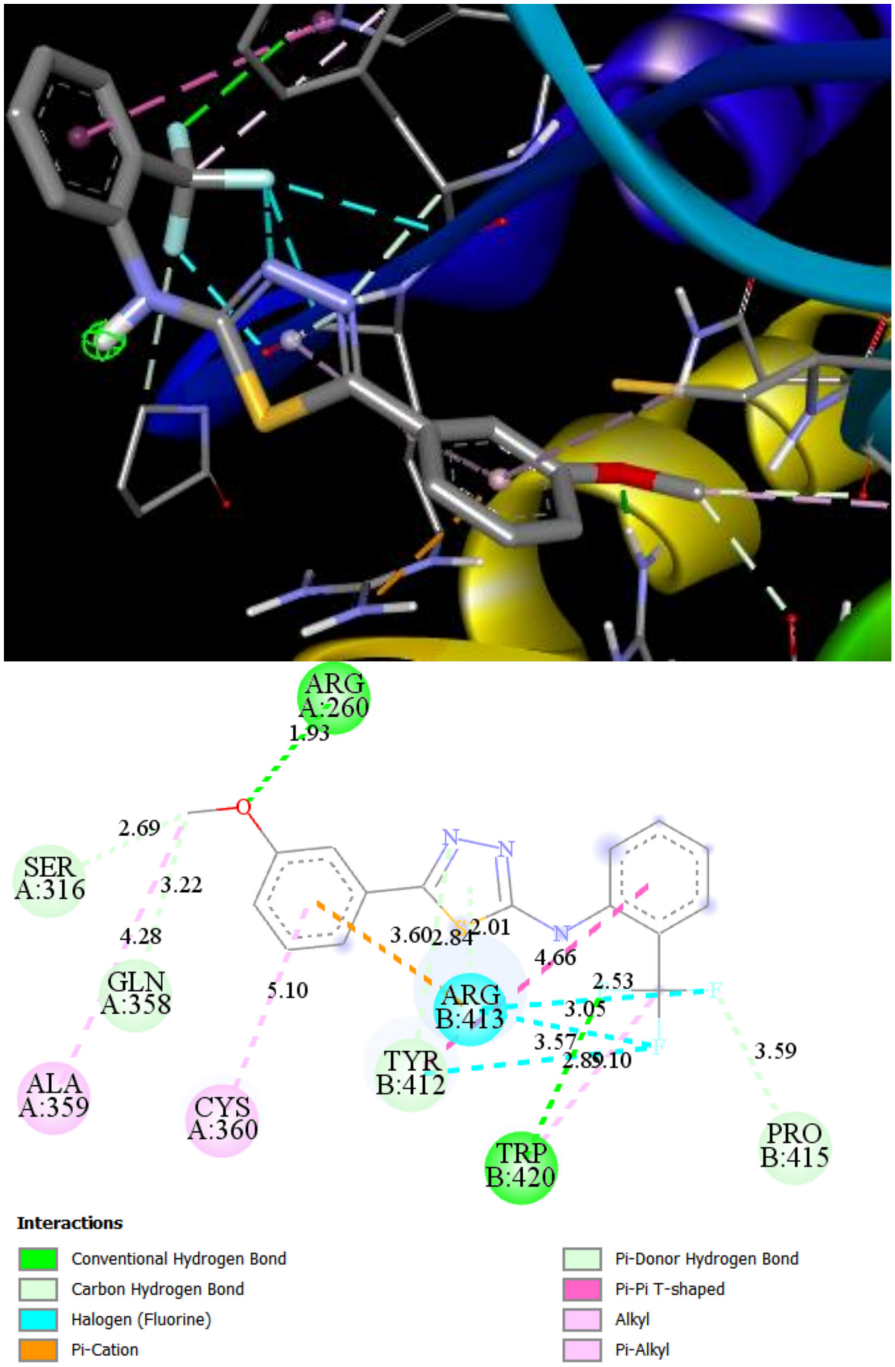
2D and 3D interaction maps of **ST10** with Caspase 8.

The most active according to the biological assays, **ST10** shows the best binding affinities (−7.34 kcal/mol, Ki = 4.16 μM) for Caspase 8 and (−7.43 kcal/mol, Ki = 3.56 μM) for Bax-protein. In the ST10–Caspase8 complex, the hydrogen bonds are observed with ARG260 (1.93 Å) and TRP420 (2.89 Å) amino acid residues (Figure 6). The trifluoromethyl group forms a number of halogen interactions with electron-poor species of TYR12 and ARG413. Lipophilic aminoacids ALA359, CYS360, ARG413, and ARG413 connect to the **ST10** by different types of hydrophobic interactions. In addition, carbon and Pi-donor hydrogen bonds contribute to the stabilization of the ST10–Caspase 8 complex.

**Figure 7:**
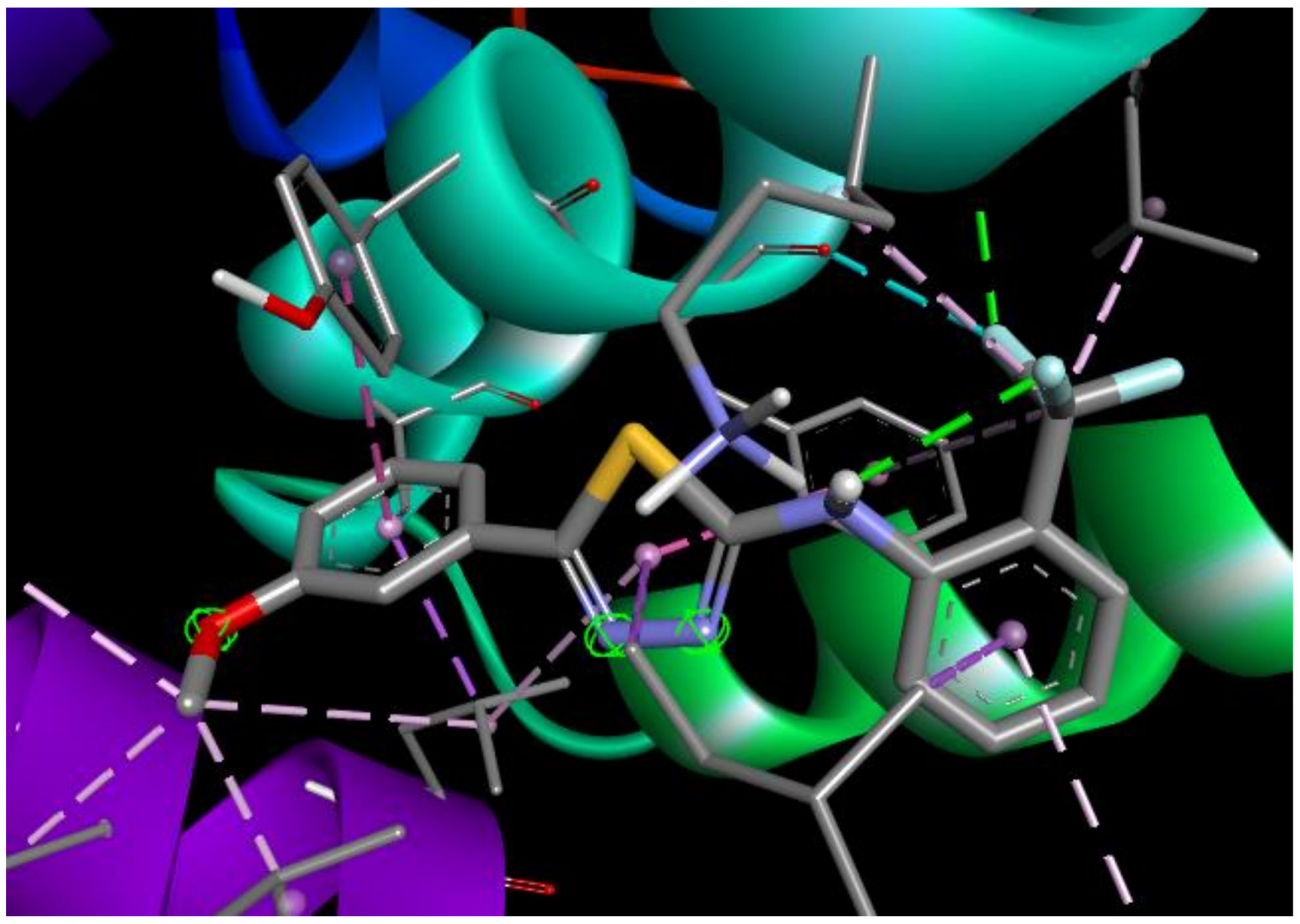

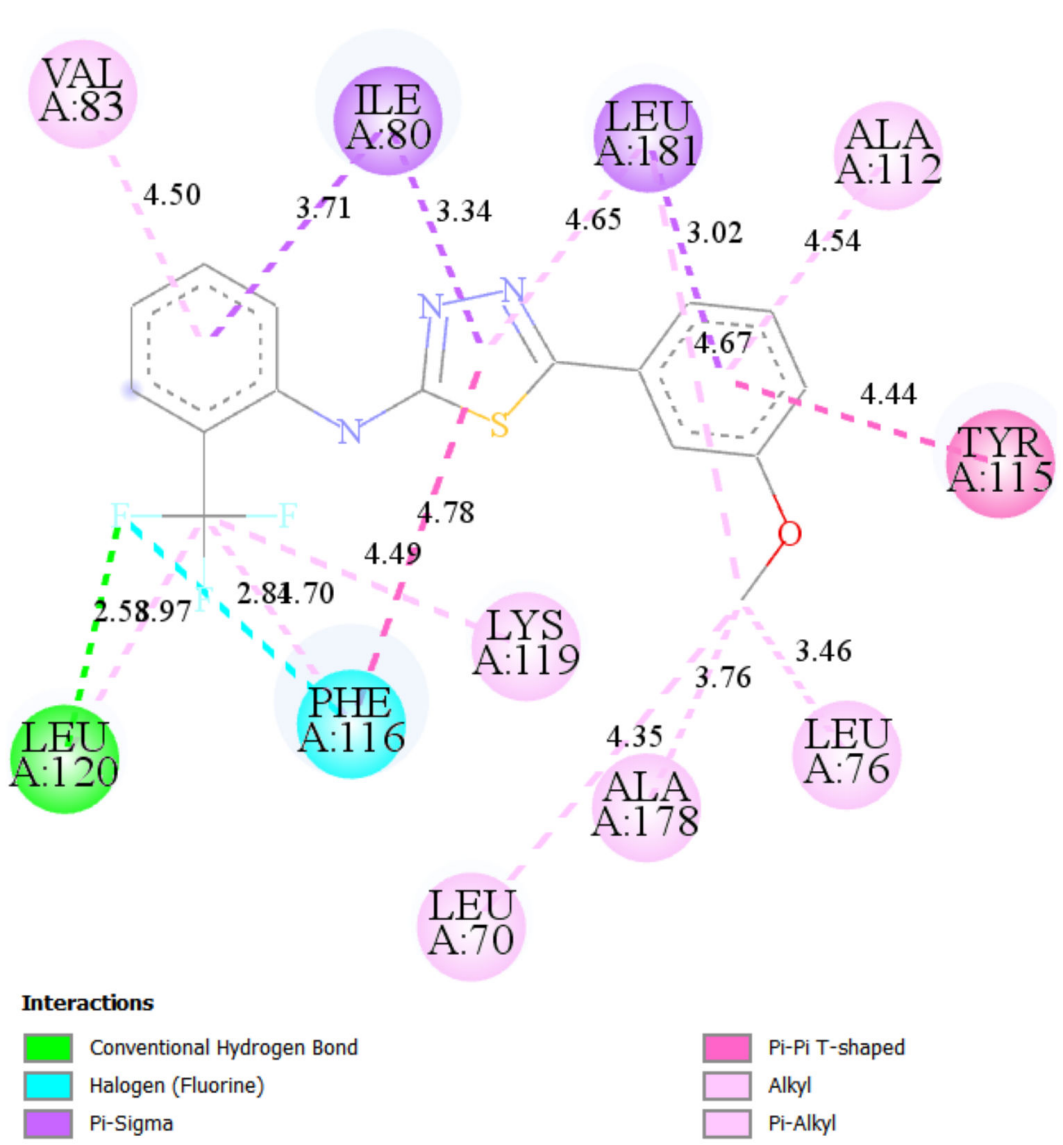
2D and 3D interaction maps of **ST10** with Bax.

Docking simulations of ST10–Bax interaction proposed possible the molecule position within the channel, made by lipophilic amino acids from α2, α3, α5, and α9 helixes of Bax (Figure 7). Additionally, LEU120 forms the hydrogen bond with fluor with a length of 2.58 Å. Such a position of **ST10** inside Bax would stabilize the whole protein and increase its apoptotic activity.

From the in silico docking simulations, it is quite evident that 2-(phenylamino)-5-(3-methoxyphenyl)-1,3,4-thiadiazole scaffold is an interesting core for building new molecules with the affinity to Caspase 3 and Caspase 8 as potent anticancer agents.

### 2.4. ADMET

The drug-likeness calculation is a prediction that determines whether a particular pharmacological agent has properties consistent with its ability for using as an orally active drug. This prediction is based on an already established concept by Lipinski et al., called the Lipinski rule of five. Lipinski’s rules predict the molecular properties related to the pharmacokinetics of molecules. According to this rule, the compound that has MLogP <5, molecular weight <500, the number of hydrogen bond acceptors <10, and the number of hydrogen bond donors <5 possesses good oral bioavailability [33]. The SwissADME computed results demonstrated that **ST1**–**ST15** satisfies Lipinski’s rule of five with zero violations. The predicted logP values revealed that they have optimal lipophilicity (ranging from 1.6 to 3.16) (Table 3). According to obtained data, all compounds are suited to Lipinski’s rule of five, possess high human intestinal absorption but do not get through the blood–brain barrier.

According to obtained data, all compounds are suited to Lipinski’s rule of five, which allows supposing **ST1**–**ST15** as drug-like molecules.

Predictions of ADMET properties for **ST1**–**ST15** also were made using the admetSAR portal (http://lmmd.ecust.edu.cn/admetsar2/ accessed on 20 November 2021), and the calculated ADMET parameters were highlighted in Table 4. The results indicated that all the synthesized compounds **ST1**–**ST15** could be administrated orally. In addition, all the compounds demonstrate high CYP inhibitory promiscuity. Additionally, **ST11**–**ST15** belongs to the III category of toxicity, which is characterized as slightly toxic. Category III includes compounds with LD_50_ values greater than 500 mg/kg but less than 5000 mg/kg, but **ST8**–**ST9** and **ST13**–**ST15** would possess carcinogenic activities according to in silico simulation results. Nevertheless, **ST10** does not have this violation.

## 3. Materials and Methods

### 3.1. Chemistry

All the substances were purchased from Sigma-Aldrich (Munich, Germany) and were used without further purification. The ^1^H and ^13^C NMR spectra were recorded on the BrukerAvance 300 (Bruker BioSpin GmbH, Rheinstetten, Germany) in DMSO-d6 with tetramethylsilane as the internal standard. IR spectra were recorded by Nicolet 6700 spectrometer (Thermo Scientific, Philadephia, PA, USA). The melting points were determined on the Stuart SMP50 melting point apparatus (Cole Parmer Ltd, Stone, UK) and are uncorrected. The purity of the compounds and the progress of the reaction were monitored by TLC (aluminum sheet 60 F254 plates (Merck Co., Kenilworth, NJ, USA). We used the solvent system CHCl3/EtOH (10:1, *v*/*v*). The elemental analyses were determined by a PerkinElmer 2400 series II CHNS/O analyzer (Waltham, MA, USA).

#### 3.1.1. Synthesis of Thiosemicarbazide Derivatives

##### Synthesis of **S1**, **S2**, **S3**, **S7**, **S8**, **S9**, **S11**, and **S13**

First, 0.001 mol of 3-methoxybenzhydrazide and 5 mL of ethanol were placed in a round bottom flask. It was heated under reflux until a clear solution was obtained. An equimolar amount of the appropriate aryl isothiocyanate * was then added to the mixture. It was heated at the boiling point for 1 h. The solution was then cooled until the product precipitated completely. The resulting solid was filtered off and washed with diethyl ether and water.

* 2-chlorophenyl isothiocyanate for **S1**, 3-chlorophenyl isothiocyanate for **S2**, 4-chlorophenyl isothiocyanate for **S3**, 2-methoxyphenyl isothiocyanate for **S7**, 3-methoxyphenyl isothiocyanate for **S8**, 4-methoxyphenyl isothiocyanate for **S9**, 3-trifluoromethylphenyl isothiocyanate for **S11**, 2-nitrophenyl isothiocyanate for **S13**.

##### Synthesis of **S4**, **S5**, **S6**, **S10**, **S12**, **S14**, and **S15**

First, 0.001 mole of 3-methoxybenzhydrazide was dissolved in 5 mL of ethanol by heating under reflux. An equimolar amount of the appropriate aryl isothiocyanate * was then added to the mixture. The reaction mixture was heated until the product precipitated. Compounds **S4**, **S14**, and **S15** immediately precipitated. Derivatives **S5**, **S6**, **S10**, and **S12** were heated for 30 min until a solid precipitated. The resulting precipitate was filtered off and washed with diethyl ether and water.

* 2-fluorophenyl isothiocyanate for **S4**, 3-fluorophenyl isothiocyanate for **S5**, 4-fluorophenyl isothiocyanate for **S6**, 2-trifluoromethylphenyl isothiocyanate for **S10**, 4-trifluoromethylphenyl isothiocyanate for **S12**, 3-nitrophenyl isothiocyanate for **S14**, 4-nitrophenyl isothiocyanate for **S15**.

4-(2-Chlorophenyl)-1-(3-methoxyphenyl)thiosemicarbazide (**S1**)

CAS 891376-78-6

Yield 77% (0.235 g), m.p. 168–170 °C. Spectral data were as follows: IR (cm^–1^) KBr: 3184 (NH), 2963 (CH aliph.), 1680 (C = O), 1583 (CH arom.), 1334 (C=S), 1267 (C-O-C). ^1^H NMR (DMSO-d6) δ (ppm): 3.82 (s, 3H, CH_3_), 7.16 (ddd, 1H, ArH, *J* = 8.2 Hz, *J* = 2.6 Hz, *J* = 1.0 Hz), 7.28 (t, 1H, ArH, *J* = 8.5 Hz), 7.35 (t, 1H, ArH, *J* = 5.8 Hz), 7.42 (t, 1H, ArH, *J* = 8.0 Hz), 7.45–7.56 (m, 4H, ArH), 9.66 (s, 1H, NH), 9.87 (s, 1H, NH), 10.61 (s, 1H, NH). ^13^C NMR (DMSO-d6) δ (ppm): 55.83, 113.69, 118.15, 120.72, 127.51, 128.42, 129.74, 129.88, 131.30, 131.30, 131.76, 134.27, 137.32, 159.51, 166.43, 182,37. Elemental analysis for C_15_H_14_ClN_3_O_2_S. Calculated: C 53.65; H 4.20; N 12.51. Found: C 53.35; H 4.10; N 12.40.

4-(3-Chlorophenyl)-1-(3-methoxyphenyl)thiosemicarbazide (**S2**)

CAS 903081-85-6

Yield 75% (0.252 g), m.p. 170–173 °C. Spectral data were as follows: IR (cm^–1^) KBr: 3181 (NH), 2969 (CH aliph.), 1664 (C = O), 1587 (CH arom.), 1336 (C=S), 1259 (C-O-C). ^1^H NMR (DMSO-d6) δ (ppm): 3.82 (s, 3H, CH_3_), 7.16 (dd, 1H, ArH, *J* = 8.3 Hz, *J* = 1.7 Hz), 7.32–7.34 (m, 1H, ArH), 7.36 (t, 2H, ArH, *J* = 8.1 Hz), 7.43 (t, 2H, ArH, *J* = 7.9 Hz), 7.47 (d, 1H, ArH, *J* = 8.0 Hz), 7.51–7.54 (m, 2H, ArH), 9.90 (s, 2H, 2NH), 10.57 (s, 1H, NH). ^13^C NMR (DMSO-d6) δ (ppm): 55.83, 113.53, 118.21, 120.64, 124.75, 125.22, 125.75, 129.94, 134.23, 159.56, 166.22, 181.45. Elemental analysis for C_15_H_14_ClN_3_O_2_S. Calculated: C 53.65; H 4.20; N 12.51. Found: C 53.40; H 4.10; N 12.43.

4-(4-Chlorophenyl)-1-(3-methoxyphenyl)thiosemicarbazide (**S3**)

CAS 891376-71-9

Yield 78% (0.262 g), m.p. 173–175 °C. Spectral data were as follows: IR (cm^–1^) KBr: 3151 (NH), 2963 (CH aliph.), 1680 (C = O), 1583 (CH arom.), 1334 (C=S), 1267 (C-O-C). ^1^H NMR (DMSO-d6) δ (ppm): 3.87 (s, 3H, CH_3_), 7.21 (dd, 1H, ArH, *J* = 8.2 Hz, *J* = 3.6 Hz), 7.44 (d, 2H, ArH, *J* = 8.8 Hz), 7.48 (t, 2H, ArH, *J* = 7.9 Hz), 7.53–7.60 (m, 3H, ArH), 9.89 (s, 2H, 2NH), 10.61 (s, 1H, NH). ^13^C NMR (DMSO-d6) δ (ppm): 55.83, 113.58, 118.18, 120.62, 128.32, 129.91, 134.27, 138.73, 159.55, 166.20, 181.58. Elemental analysis for C_15_H_14_ClN_3_O_2_S. Calculated: C 53.65; H 4.20; N 12.51. Found: C 53.52; H 4.20; N 12.45.

4-(2-Fluorophenyl)-1-(3-methoxyphenyl)thiosemicarbazide (**S4**)

CAS 891102-96-8

Yield 93% (0.297g), m.p. 182–185 °C. Spectral data were as follows: IR (cm^–1^) KBr: 3153 (NH), 2973 (CH aliph.), 1662 (C = O), 1588 (CH arom.), 1336 (C=S), 1241 (C-O-C). ^1^H NMR (DMSO-d6) δ (ppm): 3.81 (s, 3H, CH_3_), 7.15 (ddd, 1H, ArH, *J* = 8.2 Hz, *J* = 2.6 Hz), 7.19 (t, 1H, ArH, *J* = 8.0 Hz), 7.22–7.25 (m, 1H, ArH), 7.27–7.34 (m, 2H, ArH), 7.51 (t, 1H, ArH, *J* = 7.9 Hz), 7.66–7.71 (m, 2H, ArH), 9.58 (s, 1H, NH), 9.86 (s, 1H, NH),10.59 (s, 1H, NH). ^13^C NMR (DMSO-d6) δ (ppm): 55.82, 113.65, 116.15 (d, *J* = 19.9 Hz), 118.16, 120.70, 124.35, 127.73, 128.59, 129.87, 131.13, 134.26, 157.05, 159.51, 166.31, 182.68. Elemental analysis for C_15_H_14_FN_3_O_2_S. Calculated: C 56.41; H 4.42; N 13.16. Found: C 56.35; H 4.32; N 13.10.

4-(3-Fluorophenyl)-1-(3-methoxyphenyl)thiosemicarbazide (**S5**)

CAS 891103-04-1

Yield 65% (0.208 g), m.p. 180–182 °C. Spectral data were as follows: IR (cm^–1^) KBr: 3171 (NH), 2962 (CH aliph.), 1664 (C = O), 1571 (CH arom.), 1356 (C=S), 1261 (C-O-C). ^1^H NMR (DMSO-d6) δ (ppm): 3.83 (s, 3H, CH_3_), 6.98–7.01 (m, 1H, ArH), 7.16 (dd, 1H, ArH, *J* = 8.2 Hz, *J* = 2.6 Hz), 7.31–7.32 (m, 1H, ArH), 7.34–7.38 (m, 1H, ArH), 7.43 (t, 1H, ArH, *J* = 7.9 Hz), 7.52–7.55 (m, 2H, ArH), 9.88 (s, 2H, NH), 10.56 (s, 1H, NH). ^13^C NMR (DMSO-d6) δ (ppm): 55.82, 113.31, 113.54, 118.20, 120.59, 129.93, 134.23, 141.43, 141.57, 159,54, 166.18, 181.09. Elemental analysis for C_15_H_14_FN_3_O_2_S. Calculated: C 56.41; H 4.42; N 13.16. Found: C 56.30; H 4.35; N 13.15.

4-(4-Fluorophenyl)-1-(3-methoxyphenyl)thiosemicarbazide (**S6**)

CAS 905231-63-2

Yield 95% (0.303 g), m.p. 175–177 °C. Spectral data were as follows: IR (cm^–1^) KBr: 3173 (NH), 2829 (CH aliph.), 1667 (C = O), 1582 (CH arom.), 1316 (C=S), 1280 (C-O-C). ^1^H NMR (DMSO-d6) δ (ppm): 3.81 (s, 3H, CH_3_), 7.15–7.18 (m, 3H, ArH), 7.42 (t, 3H, ArH, *J* = 7.9 Hz), 7.52–7.55 (m, 2H, ArH), 9.77 (s, 2H, NH),10.54 (s, 1H, NH). ^13^C NMR (DMSO-d6) δ (ppm): 57.40, 113.58, 115.15, 117.73, 120.63, 128.66, 129.89, 134.31, 136.05, 159.53, 160.75, 166.22, 181.88. Elemental analysis for C_15_H_14_FN_3_O_2_S. Calculated: C 56.41; H 4.42; N 13.16. Found: C 56.40; H 4.35; N 13.15.

1-(3-Methoxyphenyl)-4-(2-methoxyphenyl)thiosemicarbazide (**S7**)

CAS 891370-81-3

Yield 89% (0.295 g), m.p. 135–137 °C. Spectral data were as follows: IR (cm^–1^) KBr: 3166 (NH), 2967 (CH aliph.), 1688 (C = O), 1593 (CH arom.), 1356 (C=S), 1279 (C-O-C). ^1^H NMR (DMSO-d6) δ (ppm): 3.83 (s, 3H, CH_3_), 3.88 (s, 3H, CH_3_), 6.98 (d, 1H, ArH; *J* = 8.4 Hz), 9.22 (s, 1H, NH), 7.04–7.08 (m, 1H, ArH), 7.28 (dd, 1H, ArH, *J* = 8.3 Hz, *J* = 2.0 Hz), 7.35–7.45 (m, 4H, ArH), 8.58 (d, 1H, NH, *J* = 2.0 Hz), 9.90 (s, 1H, NH). ^13^C NMR (DMSO-d6) δ (ppm): 55.81, 56.20, 107.20, 113.37, 118.25, 119.74, 126.16, 128.26, 130.05, 133.29, 151.84, 158.87, 166.48. Elemental analysis for C_16_H_17_N_3_O_3_S. Calculated: C 57.99; H 5.17; N 12.68. Found: C 57.75; H 5.12; N 12.50.

1-(3-Methoxyphenyl)-4-(3-methoxyphenyl)thiosemicarbazide (**S8**)

CAS 891369-83-8

Yield 87% (0.288 g), m.p. 172–175 °C. Spectral data were as follows: IR (cm^–1^) KBr: 3173 (NH), 2944 (CH aliph.), 1667 (C = O), 1575 (CH arom.), 1357 (C=S), 1251 (C-O-C). ^1^H NMR (DMSO-d6) δ (ppm): 3.73 (s, 3H, CH_3_), 3.81 (s, 3H, CH_3_), 6.73 (dd, 1H, ArH, *J* = 8.1 Hz, *J* = 2.5 Hz), 7.02–7.05 (m, 1H, ArH), 7.15 (ddd, 1H, ArH, *J* = 8.2 Hz, *J* = 2.7 Hz), 7.22 (t, 1H, ArH, *J* = 8.1 Hz), 7.41 (t, 1H, ArH, *J* = 7.9 Hz), 7.50–7.54 (m, 2H, ArH), 9.72 (s, 2H, 2NH), 10.51 (s, 1H, NH). ^13^C NMR (DMSO-d6) δ (ppm): 54.92, 55.78, 107.20, 113.01, 116.27, 121.37, 127.79, 129.89, 133.29, 140.81, 158.87, 167.54. Elemental analysis for C_16_H_17_N_3_O_3_S. Calculated: C 57.99; H 5.17; N 12.68. Found: C 57.78; H 5.10; N 12.60.

1-(3-methoxyphenyl)-4-(4-methoxyphenyl)thiosemicarbazide (**S9**) [34]

All data are the same as in our previous work.

4-(2-Trifluoromethylphenyl)1-(3-methoxyphenyl)thiosemicarbazide (**S10**)

CAS 891611-85-1

Yield 43% (0.159 g), m.p. 153–155 °C. Spectral data were as follows: IR (cm^–1^) KBr: 3152 (NH), 2962 (CH aliph.), 1667 (C = O), 1583 (CH arom.), 1368 (C=S), 1266 (C-O-C). ^1^H NMR (DMSO-d6) δ (ppm): 3.82 (s, 3H, CH_3_), 7.15 (dd, 1H, ArH, *J* = 8.2 Hz, *J* = 2.6 Hz), 7.42 (t, 1H, ArH, *J* = 7.9 Hz), 7.44–7.46 (m, 1H, ArH), 7.49 (t, 1H, ArH, *J* = 7.7 Hz), 7.52–7.55 (m, 2H, ArH), 7.68–7.72 (m, 2H, ArH), 9.58 (s, 1H, NH), 9.86 (s, 1H, NH), 10.57 (s, 1H, NH). ^13^C NMR (DMSO-d6) δ (ppm): 55.80, 113.69, 118.11, 120.71, 126.58,127.77, 129.86, 133.00, 134.30, 137.78, 138.27, 159.50, 166.53, 183.19. Elemental analysis for C_16_H_14_F_3_N_3_O_2_S. Calculated: C 52.03; H 3.82; N 11.38. Found: C 51.89; H 3.66; N 11.29.

4-(3-Trifluoromethylphenyl)-1-(3-methoxyphenyl)thiosemicarbazide (**S11**)

CAS 903815-86-1

Yield 95% (0.351 g), m.p. 178–180 °C. Spectral data were as follows: IR (cm^–1^) KBr: 3170 (NH), 2838 (CH aliph.), 1660 (C = O), 1579 (CH arom.), 1333 (C=S), 1287 (C-O-C). ^1^H NMR (DMSO-d6) δ (ppm): 3.83 (s, 3H, CH_3_), 7.17 (dd, 1H, ArH, *J* = 8.3 Hz, *J* = 2.6 Hz), 7.44 (t, 2H, ArH, *J* = 7.9 Hz), 7.59 (m, 3H, ArH), 7.85 (d, 2H, ArH, *J* = 7.8 Hz) 9.99 (s, 2H, 2NH), 10.61 (s, 1H, NH). ^13^C NMR (DMSO-d6) δ (ppm): 55.83, 113.60, 118.23, 120.63, 121.85, 122.37, 123.65, 125.45, 129.50, 129.96, 134.15, 140.57, 159.58, 166.22, 181.54. Elemental analysis for C_16_H_14_F_3_N_3_O_2_S. Calculated: C 52.03; H 3.82; N 11.38. Found: C 51.93; H 3.75; N 11.32.

4-(4-Trifluoromethylphenyl)-1-(3-methoxyphenyl)thiosemicarbazide (**S12**)

Yield 65% (0.240 g), m.p. 195–197 °C. Spectral data were as follows: IR (cm^–1^) KBr: 3161 (NH), 2969 (CH aliph.), 1669 (C = O), 1579 (CH arom.), 1322 (C=S), 1289 (C-O-C). ^1^H NMR (DMSO-d6) δ (ppm): 3.83 (s, 3H, CH_3_), 7.17 (dd, 1H, ArH, *J* = 8.5 Hz, *J* = 2.9 Hz), 7.43 (t, 1H, ArH, *J* = 7.9 Hz), 7.52–7.55 (m, 2H, ArH), 7.70 (d, 2H, ArH, *J* = 7.9 Hz), 7.76 (bs, 2H, ArH), 9.98 (s, 2H, 2NH), 10.60 (s, 1H, NH). ^13^C NMR (DMSO-d6) δ (ppm): 55.83, 113.59, 118.24, 120.63, 123.91, 125.60 (d, *J* = 34.8 Hz), 126.24, 129.95, 134.20, 143.56, 159.58, 166.22, 181.46. Elemental analysis for C_16_H_14_F_3_N_3_O_2_S. Calculated: C 52.03; H 3.82; N 11.38. Found: C 51.98; H 3.79; N 11.27.

1-(3-Methoxyphenyl)-4-(2-nitrophenyl)thiosemicarbazide (**S13**)

Yield 88% (0.305 g), m.p. 150–153 °C. Spectral data were as follows: IR (cm^–1^) KBr: 3177 (NH), 2836 (CH aliph.), 1661 (C = O), 1581 (CH arom.), 1337 (C=S), 1265 (C-O-C). ^1^H NMR (DMSO-d6) δ (ppm): 3.83 (s, 3H, CH_3_), 7.18 (d, 1H, ArH, *J* = 8.3 Hz), 7.44 (t, 2H, ArH, *J* = 7.9 Hz), 7.54–7.57 (m, 2H, ArH), 7.72–7.75 (m, 1H, ArH), 7.95 (d, 1H, ArH, *J* = 8.2 Hz), 8.04 (dd, 1H, ArH, *J* = 8.2 Hz, *J* = 1.5 Hz), 10.15 (s, 1H, NH), 10.19 (s, 1H, NH), 10.73 (s, 1H, NH). ^13^C NMR (DMSO-d6) δ (ppm): 55.83, 113.60, 118.36, 120.66, 125.26, 126.87, 129.70, 130.02, 134.00, 144.20, 159.58, 166.55, 181.51. Elemental analysis for C_15_H_14_N_4_O_4_S. Calculated: C 42.02; H 4.07; N 16.18. Found: C 41.98; H 4.02; N 16.10.

1-(3-Methoxyphenyl)-4-(3-nitrophenyl)thiosemicarbazide (**S14**)

CAS 891561-77-6

Yield 96% (0.333 g), m.p. 159–162 °C. Spectral data were as follows: IR (cm^–1^) KBr: 3159 (NH), 2845 (CH aliph.), 1667 (C = O), 1579 (CH arom.), 1335 (C=S), 1261 (C-O-C). ^1^H NMR (DMSO-d6) δ (ppm): 3.82 (s, 3H, CH_3_), 7.17 (dd, 1H, ArH, *J* = 8.2 Hz, *J* = 2.9 Hz), 7.43 (t, 1H, ArH, *J* = 7.9 Hz), 7.52–7.56 (m, 2H, ArH), 7.61 (t, 1H, ArH, *J* = 8.2 Hz), 8.01 (d, 2H, ArH, *J* = 8.2 Hz), 8.41–8.45 (m, 1H, ArH), 10.08 (s, 2H, 2NH), 10.63 (s, 1H, NH). ^13^C NMR (DMSO-d6) δ (ppm): 55.85, 113.59, 118.29, 119.97, 120.64, 129.22, 132.27, 134.13, 141.01, 147.12, 159.60, 166.22, 181.44. Elemental analysis for C_15_H_14_N_4_O_4_S. Calculated: C 42.02; H 4.07; N 16.18. Found: C 41.95; H 4.00; N 16.18.

1-(3-Methoxyphenyl)-4-(4-nitrophenyl)thiosemicarbazide (**S15**)

CAS 891561-93-6

Yield 92% (0.319 g), m.p. 165–167 °C. Spectral data were as follows: IR (cm^–1^) KBr: 3159 (NH), 2842 (CH aliph.), 1651 (C = O), 1563 (CH arom.), 1328 (C=S), 1258 (C-O-C). ^1^H NMR (DMSO-d6) δ (ppm): 3.82 (s, 3H, CH_3_), 7.16 (dd, 1H, ArH, *J* = 8.7 Hz, *J* = 3.2 Hz), 7.43 (t, 1H, ArH, *J* = 7.9 Hz), 7.50–7.55 (m, 2H, ArH), 7.90 (d, 2H, ArH, *J* = 8.4 Hz), 8.21 (d, 2H, ArH, *J* = 9.1 Hz), 9.98 (s, 2H, 2NH), 10.60 (s, 1H, NH). ^13^C NMR (DMSO-d6) δ (ppm): 53.13, 113.59, 118.29, 119.74, 120.23, 120.64, 124.06, 125,37, 128.90, 130.00, 132.28, 134.11, 141.01, 158.48, 165.67, 180.15. Elemental analysis for C_15_H_14_N_4_O_4_S. Calculated: C 42.02; H 4.07; N 16.18. Found: C 41.95; H 4.00; N 16.15.

#### 3.1.2. Synthesis of 1,3,4-thiadiazoles ST1–ST15

First, 0.2 g of the thiosemicarbazide derivatives obtained in stage I was weighed and placed in conical flasks. Then, 0.5 mL of concentrated sulfuric acid (VI) was added dropwise with thorough stirring. The reaction mixture was stirred until the precipitate dissolved, and 2 h were given for cooling at room temperature. Then, finely crushed ice was added to the reaction flasks and mixed intensively. A solid precipitated. After the complete dissolution of the ice, the solid product was filtered off. The product was dried thoroughly with filter paper. Then, crystallization from butanol was performed to obtain pure **ST1**–**ST15** compounds.

2-(2-Chlorophenylamino)-5-(3-methoxyphenyl)-1,3,4-thiadiazole (**ST1**)

Yield 35% (0.078 g), m.p. 115–118 °C. Spectral data were as follows: IR (cm^–1^) KBr: 3151 (NH), 2883 (CH aliph.), 1665 (C=N), 1584 (CH arom.), 1261 (C-O-C), 715 (C-S). ^1^H NMR (DMSO-d6) δ (ppm): 3.84 (s, 3H, CH_3_), 7.08 (d, 1H, ArH, *J* = 4.0 Hz), 7.12–7.15 (m, 1H, ArH), 7.40–7.45 (m, 4H, ArH), 7.53 (d, 1H, ArH, *J* = 4.0 Hz), 8.30 (bs, 1H, ArH), 10.03 (s, 1H, NH). ^13^C NMR (DMSO-d6) δ (ppm): 55.77, 111.85, 116.59, 119.88, 122.64, 123.86, 125.01, 128.41, 130.23, 130.23, 130.95, 131.99, 137.77, 160.10, 165.36. Elemental analysis for C_15_H_12_ClN_3_OS. Calculated: C 56.69; H 3.81; N 13.22. Found: C 56.55; H 3.75; N 13.20.

2-(3-Chlorophenylamino)-5-(3-methoxyphenyl)-1,3,4-thiadiazole (**ST2**)

Yield 53% (0.127 g), m.p. 192–194 °C. Spectral data were as follows: IR (cm^–1^) KBr: 3059 (NH), 2808 (CH aliph.), 1625 (C=N), 1564 (CH arom.), 1268 (C-O-C), 760 (C-S). ^1^H NMR (DMSO-d6) δ (ppm): 3.84 (s, 3H, CH_3_), 7.06–7.11 (m, 2H, ArH), 7.36–7.47 (m, 5H, ArH), 7.95 (t, 1H, ArH, *J* = 2.1 Hz), 10.78 (s, 1H, NH). ^13^C NMR (DMSO-d6) δ (ppm): 55.81, 111.98, 116.44, 116.75, 117.40, 120.03, 122.08, 131.01, 131.22, 131.82, 134.01, 142.23, 158.61, 160.14, 164.18. Elemental analysis for C_15_H_12_ClN_3_OS. Calculated: C 56.69; H 3.81; N 13.22. Found: C 56.65; H 3.78; N 13.15.

2-(4-Chlorophenylamino)-5-(3-methoxyphenyl)-1,3,4-thiadiazole (**ST3**)

CAS 1202973-36-1

Yield 70% (0.174 g), m.p. 205–207 °C. Spectral data were as follows: IR (cm^–1^) KBr: 3148 (NH), 2833 (CH aliph.), 1582 (CH arom.), 1283 (C-O-C), 757 (C-S). ^1^H NMR (DMSO-d6) δ (ppm): 3.84 (s, 3H, CH_3_), 7.07–7.09 (m, 1H, ArH), 7.39 (s, 3H, ArH), 7.42 (t, 2H, ArH, *J* = 3.9 Hz), 7.73–7.67 (m, 2H, ArH), 10.70 (s, 1H, NH). ^13^C NMR (DMSO-d6) δ (ppm): 55.79, 111.95, 116.16, 119.52 120.91, 124.86, 129.42, 130.56, 131.89, 139.86, 158.71, 160.11, 164.29. Elemental analysis for C_15_H_12_ClN_3_OS. Calculated: C 56.69; H 3.81; N 13.22. Found: C 56.59; H 3.55; N 13.18.

2-(2-Fluorophenylamino)-5-(3-methoxyphenyl)-1,3,4-thiadiazole (**ST4**)

Yield 45% (0.126 g), m.p. 125–128 °C. Spectral data were as follows: IR (cm^–1^) KBr: 3205 (NH), 2836 (CH aliph.), 1619 (C=N), 1581 (CH arom.), 1289 (C-O-C), 749 (C-S). ^1^H NMR (DMSO-d6) δ (ppm): 3.84 (s, 3H, CH_3_), 7.08–7.10 (m, 2H, ArH), 7.24 (t, 1H, ArH, *J* = 7.8 Hz), 7.31 (t, 1H, ArH, *J* = 9.8 Hz), 7.41–7.45 (m, 3H, ArH), 8.38–8.40 (m, 1H, ArH), 10.32 (s, 1H, NH). ^13^C NMR (DMSO-d6) δ (ppm): 55.80, 111.87, 115.74, 115.86, 116.65, 119.94, 121.19, 123.72, 123.77, 125.28, 128.83, 128.90, 130.98, 131.97, 158.98, 160.12, 164.75. Elemental analysis for C_15_H_12_FN_3_OS. Calculated: C 59.79; H 4.01; N 13.95. Found: C 59.56; H 3.87; N 13.65.

2-(3-Fluorophenylamino)-5-(3-methoxyphenyl)-1,3,4-thiadiazole (**ST5**)

Yield 66% (0.129 g), m.p. 197–199 °C. Spectral data were as follows: IR (cm^–1^) KBr: 3168 (NH), 2837 (CH aliph.), 1620 (C=N), 1599 (CH arom.), 1217 (C-O-C), 747 (C-S). ^1^H NMR (DMSO-d6) δ (ppm): 3.84 (s, 3H, CH_3_), 6.81–6.87 (m, 1H, ArH), 7.08 (dt, 1H, ArH, *J* = 6.8 Hz, *J* = 2.5 Hz), 7.30–7.35 (m, 1H, ArH), 7.37–7.46 (m, 4H, ArH), 7.71 (dt, 1H, ArH, *J* = 11.8 Hz, *J* = 2.3 Hz), 10.80 (s, 1H, NH). ^13^C NMR (DMSO-d6) δ (ppm): 54.79, 104.87 (d, *J* = 27.0 Hz), 108.82 (d, *J* = 21.1 Hz), 111.31, 113.44, 116.31, 120.02, 131.19 (d, *J* = 9.8 Hz), 142.54 (d, *J* = 11.5 Hz), 158.51, 160.13, 162.23, 163.83, 164.23. Elemental analysis for C_15_H_12_FN_3_OS. Calculated: C 59.79; H 4.01; N 13.95. Found: C 59.68; H 3.99; N 13.78.

2-(4-Fluorophenylamino)-5-(3-methoxyphenyl)-1,3,4-thiadiazole (**ST6**)

Yield 46% (0.132 g), m.p. 172–174 °C. Spectral data were as follows: IR (cm^–1^) KBr: 3177 (NH), 2831 (CH aliph.), 1628 (C=N), 1579 (CH arom.), 1280 (C-O-C), 774 (C-S). ^1^H NMR (DMSO-d6) δ (ppm): 3.83 (s, 3H, CH_3_), 7.07 (dt, 1H, ArH, *J* = 7.5 Hz, *J* = 2.0 Hz), 7.21 (t, 2H, ArH, *J* = 8.9 Hz), 7.38–7.45 (m, 3H, ArH), 7.65–7.70 (m, 2H, ArH), 10.57 (s, 1H, NH). ^13^C NMR (DMSO-d6) δ (ppm): 55.80, 111.88, 116.17 (d, *J* = 22.2 Hz), 116.58, 119.71, 119.76, 119.92, 130.96, 131.99, 137.49, 157.04, 157.84, 158.63, 160.12, 164.74. Elemental analysis for C_15_H_12_FN_3_OS. Calculated: C 59.79; H 4.01; N 13.95. Found: C 59.69; H 3.99; N 13.69.

5-(3-Methoxyphenyl)-2-(2-methoxyphenylamino)-1,3,4-thiadiazole (**ST7**)

CAS 1203294-57-8

Yield 59% (0.165 g), m.p. 185–188 °C Spectral data were as follows: IR (cm^–1^) KBr: 3264 (NH), 2941 (CH aliph.), 1598 (C=N), 1560 (CH arom.), 1285 (C-O-C), 767 (C-S). ^1^H NMR (DMSO-d6) δ (ppm): 3.83 (s, 3H, CH_3_), 3.88 (s, 3H, CH_3_), 6.70 (d, 1H, ArH, *J* = 4.0 Hz), 7.06 (dd, 1H, ArH, *J* = 0.6 Hz, *J* = 4.0 Hz), 7.28 (dd, 1H, ArH, *J* = 0.6 Hz, *J* = 4.0 Hz), 7.35–7.45 (m, 2H, ArH), 7.43 (t, 1H, ArH, *J* = 4.0 Hz), 8.59 (d, 1H, ArH, *J* = 1.0 Hz)), 9.90 (s, 1H, NH). ^13^C NMR (DMSO-d6) δ (ppm): 55.78, 56.41, 110.32, 111.80, 116.48, 117.59, 119.84, 121.08, 130.95, 132.13, 141.25, 149.15, 158.49, 160.09, 165.02. Elemental analysis for C_16_H_15_N_3_O_2_S. Calculated: C 61.32; H 4.82; N 13.41. Found: C 61.24; H 4.59; N 13.29.

5-(3-Methoxyphenyl)-2-(3-methoxyphenylamino)-1,3,4-thiadiazole (**ST8**)

CAS 1203389-12-1

Yield 47% (0.128 g), m.p. 153–155 °C.Spectral data were as follows: IR (cm^–1^) KBr: 3261 (NH), 2939 (CH aliph.), 1600 (C=N), 1560 (CH arom.), 1288 (C-O-C), 773 (C-S). ^1^H NMR (DMSO-d6) δ (ppm): 3.76 (s, 3H, CH_3_), 3.84 (s, 3H, CH_3_), 7.00 (dd, 1H, ArH, *J* = 8.4 Hz, *J* = 1.9 Hz), 7.07 (dt, 1H, ArH, *J* = 7.3 Hz, *J* = 2.4 Hz), 7.41–7.43 (m, 4H, ArH,), 7.62 (d, 1H, ArH, *J* = 8.4 Hz), 10.63 (s, 1H, NH). 1^3^C NMR (DMSO-d6) δ (ppm): 55.80, 55.86, 101.79, 108.03, 111.75, 116.71, 119.99, 129.71, 130.15, 130.96, 131.98, 142.70, 157.30, 158.08, 160.12, 164.50. Elemental analysis for C_16_H_15_N_3_O_2_S. Calculated: C 61.32; H 4.82; N 13.41. Found: C 61.34; H 4.79; N 13.49.

5-(3-Methoxyphenyl)-2-(4-methoxyphenylamino)-1,3,4-thiadiazole (**ST9**)

Yield 32% (0.078 g), m.p. 145–148 °C. Spectral data were as follows: IR (cm^–1^) KBr: 3077 (NH), 2838 (CH aliph.), 1624 (C=N), 1583 (CH arom.), 1283 (C-O-C), 779 (C-S). ^1^H NMR (DMSO-d6) δ (ppm):) δ (ppm): 3.83 (s, 6H, CH_3_), 7.08 (dt, 1H, ArH, *J* = 7.2 Hz, *J* = 2.4 Hz), 7.40–7.43 (m, 5H, Ar Hz), 7.70 (d, 2H, ArH, *J* = 8.9 Hz), 10.70 (s, 1H, NH). ^13^C NMR (DMSO-d6) δ (ppm): 52.89, 60.83, 109.46, 110.71, 114.19, 116.49, 119.41, 120.11, 131.35, 132.07, 134.83, 154.52, 157.03, 159.27, 165.46. Elemental analysis for C1_6_H_15_N_3_O_2_S. Calculated: C 61.32; H 4.82; N 13.41. Found: C 61.28; H 4.79; N 13.20.

2-(2-Trifluorometylophenylamino)-5-(3-methoxyphenyl)-1,3,4-thiadiazole (**ST10**)

Yield 22% (0.033 g), m.p. 167–169 °C. Spectral data were as follows: IR (cm^–1^) KBr: 3130 (NH), 2834 (CH aliph.), 1618 (C=N), 1587 (CH arom.), 1270 (C-O-C), 746 (C-S). ^1^H NMR (DMSO-d6) δ (ppm): 3.81 (s, 3H, CH_3_), 7.03–7.07 (m, 1H, ArH), 7.32–7.43 (m, 4H, ArH), 7.69–7.77 (m, 2H, ArH), 7.89 (bs, 1H, ArH), 9.92 (s, 1H, NH).^13^C NMR (DMSO-d6) δ (ppm): 53.92, 112.45, 115.93, 119.60, 123.33, 125.65, 127.06, 130.93, 130.61, 133.33, 161.25. Elemental analysis for C_16_H_12_N_3_F_3_OS. Calculated: C 54.70; H 3.44; N 11.96. Found: C 54.56; H 3.37; N 11.68.

2-(3-Trifluorometylophenylamino)-5-(3-methoxyphenyl)-1,3,4-thiadiazole (**ST11**)

Yield 63% (0.210 g), m.p. 183–185 °C. Spectral data were as follows: IR (cm^–1^) KBr: 3051 (NH), 2826 (CH aliph.), 1625 (C=N), 1582 (CH arom.), 1272 (C-O-C), 769 (C-S). ^1^H NMR (DMSO-d6) δ (ppm): 3.84 (s, 3H, CH_3_), 7.07–7.11 (m, 1H, ArH), 7.37 (d, 1H, ArH, *J* = 7.7 Hz), 7.42–7.44 (m, 3H, ArH), 7.60 (t, 1H, ArH, *J* = 8.0 Hz), 7.77 (d, 1H, ArH, *J* = 7.8 Hz), 8.27 (bs, 1H, ArH), 10.93 (s, 1H, NH). ^13^C NMR (DMSO-d6) δ (ppm): 55.10, 111.97, 113.94, 116.82, 118.66, 120.07, 121.53, 123.74, 125.54, 130.34 (d, *J* = 31.4 Hz), 130.88 (d, *J* = 38.1 Hz), 131.78, 141.54, 158.77, 160.14, 164.17. Elemental analysis for C_16_H_12_N_3_F_3_OS. Calculated: C 54.70; H 3.44; N 11.96. Found: C 54.68; H 3.47; N 11.98.

2-(4-Trifluorometylophenylamino)-5-(3-methoxyphenyl)-1,3,4-thiadiazole (**ST12**)

CAS 1203413-88-0

Yield 34% (0.078 g), m.p. 145–147 °C. Spectral data were as follows:IR (cm^–1^) KBr: 3060 (NH), 2874 (CH aliph.), 1614 (C=N), 1581 (CH arom.), 1258 (C-O-C), 747 (C-S). ^1^H NMR (DMSO-d6) δ (ppm): 3.78 (s, 3H, CH_3_), 7.07–7.12 (m, 1H, ArH), 7.42–7.47 (m, 3H, ArH), 7.73 (d, 2H, ArH, *J* = 8.8 Hz), 7.87 (d, 2H, ArH, *J* = 8.5 Hz), 10.79 (s, 1H, NH). ^13^C NMR (DMSO-d6) δ (ppm): 55.81, 112.07, 117.76, 120.06, 122.26 (d, *J* = 32.0 Hz), 124.11, 125.91, 126.95, 131.00, 131.77, 144.18, 159.04, 160.14, 163.98. Elemental analysis for C_16_H_12_N_3_F_3_OS. Calculated: C 54.70; H 3.44; N 11.96. Found: C 54.66; H 3.47; N 11.98.

5-(3-Methoxyphenyl)-2-(2-nitrophenylamino)-1,3,4-thiadiazole (**ST13**)

Yield 18% (0.052 g), m.p. 155–158 °C. Spectral data were as follows: IR (cm^–1^) KBr: 3270 (NH), 2864 (CH aliph.), 1602 (C=N), 1587 (CH arom.), 1271 (C-O-C), 751 (C-S). ^1^H NMR (DMSO-d6) δ (ppm): 3.83 (s, 3H, CH_3_), 7.09 (dd, 1H, ArH, *J* = 7.4 Hz, *J* = 2.5 Hz), 7.26 (t, 1H, ArH, *J* = 7.4 Hz), 7.42–7.47 (m, 3H, ArH), 7.75 (t, 1H, ArH, *J* = 7.6 Hz), 8.07 (d, 1H, ArH, *J* = 8.2 Hz), 8.26 (bs, 1H, ArH), 10.62 (s, 1H, NH). ^13^C NMR (DMSO-d6) δ (ppm): 55.82, 111.99, 116.94, 120.05, 122.93, 123.73, 126.15, 131.06, 131.74, 135.08, 135.56, 138.58, 160.16. Elemental analysis for: C_15_H_12_N_4_O_3_S. Calculated: C 54.88; H 3.68; N 17.06. Found: C 54.67; H 3.49; N 17.00.

5-(3-Methoxyphenyl)-2-(3-nitrophenylamino)-1,3,4-thiadiazole (**ST14**) [35]

Yield 60% (0.189 g), m.p. 160–163 °C. Spectral data were as follows: IR (cm^–1^) KBr: 3265 (NH), 2889 (CH aliph.), 1624 (C=N), 1594 (CH arom.), 1272 (C-O-C), 781 (C-S). ^1^H NMR (DMSO-d6) δ (ppm): 3.84 (s, 3H, CH_3_), 7.08–7.12 (m, 1H, ArH), 7.44–7.45 (m, 3H, ArH), 7.65 (t, 1H, ArH, *J* = 8.2 Hz), 7.86–7.91 (m, 2H, ArH), 8.84 (t, 1H, ArH, *J* = 2.2 Hz), 11.15 (s, 1H, NH). ^13^C NMR (DMSO-d6) δ (ppm): 56.37, 111.93, 112.05, 116.88, 120.11, 123.25, 130.92, 131.03, 131.71, 141.86, 148.86, 159.10, 160.15, 164.02. Elemental analysis for: C_15_H_12_N_4_O_3_S. Calculated: C 54.88; H 3.68; N 17.06. Found: C 54.77; H 3.55; N 17.02.

5-(3-Methoxyphenyl)-2-(4-nitrophenylamino)-1,3,4-thiadiazole (**ST15**)

CAS 1203176-20-8

Yield 42% (0.127 g), m.p. 162–164 °C. Spectral data were as follows: IR (cm^–1^) KBr: 3217 (NH), 2838 (CH aliph.), 1622 (C=N), 1507 (CH arom.), 1219 (C-O-C), 747 (C-S). ^1^H NMR (DMSO-d6) δ (ppm): 3.89 (s, 3H, CH_3_), 7.10–7.12 (m, 1H, ArH), 7.44–7.46 (m, 3H, ArH), 7.88 (d, 2H, ArH, *J* = 9.4 Hz), 8.28 (d, 2H, ArH, *J* = 9.4 Hz), 11.32 (s, 1H, NH). ^13^C NMR (DMSO-d6) δ (ppm): 55.84, 112.20, 116.94, 117.53, 120.15, 126.02, 131.05, 131.59, 141.41, 146.63, 159.96, 160.15, 163.53. Elemental analysis for: C_15_H_12_N_4_O_3_S. Calculated: C 54.88; H 3.68; N 17.06. Found: C 54.87; H 3.48; N 17.02.

### 3.2. Cell Culture

Two breast cancer cell lines (MCF-7 and MDA-MB-231) and fibroblasts as normal cell lines, which were purchased from American Type Culture Collection (ATCC, Manassas, VA, USA), were used to investigate the effects of the novel synthesized compounds. The cells were cultured in DMEM (Corning, Kennebunk, ME, USA), supplemented with FetalBovine Serum (Eurx, Gdansk, Poland) at concentration 10% and penicillin/streptomycin at 1%. Cells were cultured on 100 mm plates (Sarstedt, Newton, NC, USA) and then kept in an incubator that provided optimal growth conditions (5% CO_2_, temperature: 37 °C, humidity at 90–95% level). After reaching 80% confluence, cells were detached from the bottom of the plate using 0.05% trypsin supplemented with 0.2% EDTA (Gibco, San Diego, CA, USA). Then, using a hemocytometer, the number of cells was quantified and seeded at density 5 × 105 cells per well in six-well plates (Sarstedt, Newton, NC, USA) in 2 mL of DMEM. In this research, cells that obtained 80% of confluency were used.

### 3.3. Cell Viability Assay

The effect of novel synthesized derivatives (**ST1**-**ST15**) on the survival of breast cancer cells (MCF-7 and MDA-MB-231) and normal human cells(fibroblasts) was performed using the MTT assay according to the method described previously [36]. In short, both cancer cell lines were incubated 24 h with tested compounds and etoposide (reference drug) at the following concentrations: 25, 50, 75, and 100 µM. Following incubation, the medium was removed, and cells were washed three times with PBS. Then cells were incubated for 4 h with 1 mL PBS and 0.05 mL of MTT (5 mg/mL). The obtained product was dissolved in DMSO. The absorbance of the solutions was determined spectrophotometrically at λ = 570 nm using UV-VIS Helios Gamma (Unicam/ThermoFisher Scientific Inc., Cleveland, OH, USA). The compounds **ST13**–**ST15** interfere with MTT and were tested only by [3H]-thymidine incorporation.

### 3.4. Proliferation [3H]-Thymidine

The anti-proliferative activity of novel synthesized compounds **ST1**–**ST15** against breast cancer cell lines was analyzed by the method described in the literature [37]. The cells (MCF-7 and MDA-MB-231) were cultured on six-well plates (Sarstedt, Newton, NC, USA) and incubated with tested compounds and etoposide at various concentrations and 0.5 µCi [3H]-thymidine for 24 h at 37 °C. Then, the cells were rinsed twice with 1 mL of 0.05 M Tris/HCl buffer with 0.11 M NaCl, twice with 1 mL of 5% trichloroacetic acid (Stanlab, Lublin, Polska). After washing, the cells were solubilized with 1 mL of 0.1 NaOH containing 1% SDS (Sigma-Aldrich, St Louis, MO, USA). The solubilized cells were transferred to scintillation vials and filled with 2 mL of Ultima Gold XR. Reading occurred in a scintillation counter (1900 TR, TRI-CARB, Packard, Perkin Elmer, Inc., San Jose, CA, USA).

### 3.5. Docking

**Figure 8:**
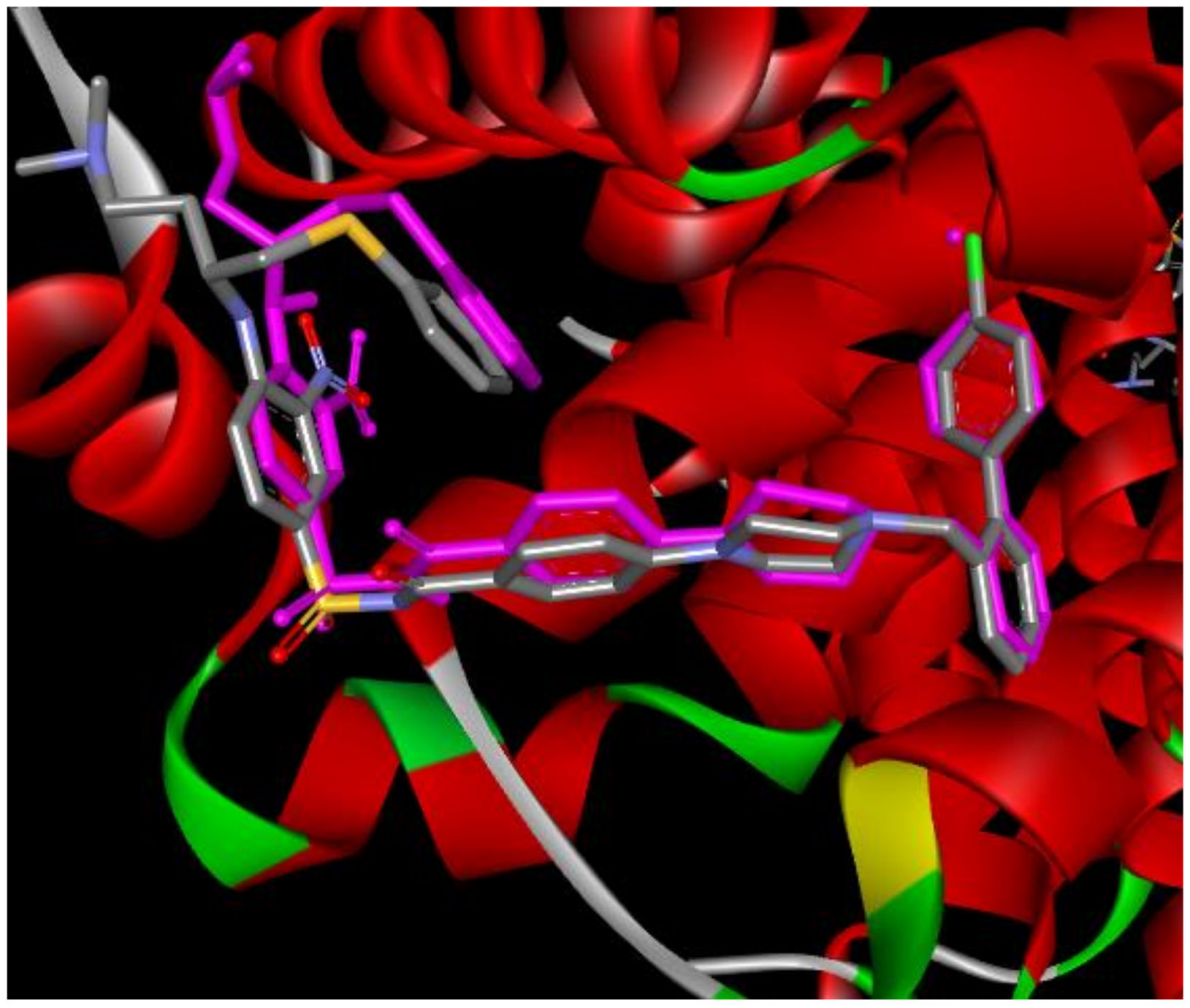
Real and predicted (pink-colored) position of N3C inside Bcl-xl protein (PDB: 2YXJ, RMSD = 1.29).

Aiming to investigate the docking scores of the bioactive conformations of the synthesized compounds and their specificity for different enzymes, which are potential anticancer targets, docking of compounds **ST1**–**ST15** were performed at the active sites of the topoisomerase IIb (PDB code 3QX3), Caspase 3 (PDB code 1GFW), Caspase 8 (PDB code 3KJN), Bcl-xl (PDB code 2YXJ), Bcl2 (PDB: 2W3L), and BAX (PDB: 1F16). All structures were downloaded from the Protein Data Bank. With the use of AutoDock Tools version 4.2.6, all bound ligands and water molecules were removed, polar hydrogen atoms were added, nonpolar hydrogen atoms were merged, and rotatable bonds were defined. Kollman charges were added. The chemical structures of the compounds were drawn by Biovia Draw, and their 3D structures were optimized by Hyperchem 7.5 using MM+ and PM3 quantum techniques, respectively. **ST1**–**ST15** were allowed with active rotatable bonds making them flexible, and the Gasteiger charges are assigned. The three-dimensional grid boxes were created, the spacing between grid points was 0.375 Å, and the grid maps representing the intact ligand in the actual docking target site were made by Auto Grid Tool. For more accurate results, we changed Lamarckian genetic algorithm (LGA) parameters from the default setting to enhanced, which includes 50 runs, 300 conformational possibilities, 50 populations, 2,500,000 energy evaluations, a maximum number of 106 energy evaluations, a mutation rate of 0.02, and a crossover rate of 0.80. Results differing by <2.0 Å in positional root-mean-square deviation (RMSD) were clustered together and represented by the result with the most favorable free energy of binding. To ensure that the ligand orientations and positions obtained from the docking studies were likely to represent valid and reasonable potential binding modes of the inhibitors, the docking methods and parameters used were validated by redocking experiments. Reference ligands were docked into the native proteins to determine the ability of the AutoDock program to reproduce the orientation and position of the ligand observed in the crystal structure Figure 8. Only for BAX protein, Zinc 14750348 was selected as reference ligands according to reported data [38].

### 3.6. In Silico Drug-Likeness Prediction

The drug-likeness of the synthesized compounds (**ST1**–**ST15**) were estimated by calculating molecular descriptors using freely accessible web server Swiss ADME (http://www.swissadme.ch/index.php accessed on 20 November 2021) [39]. ADMET properties were computed by the admetSAR portal (http://lmmd.ecust.edu.cn/admetsar2/ accesed on 20 November 2021).

## 4. Conclusions

New thiosemicarbazide and 1,3,4-thiadiazole derivatives were synthesized and evaluated for their in vitro anticancer activity. The results showed that in both breast cancer cell lines, the strongest anti-proliferative activity was exerted by compound **ST10**. The IC_50_ values of this compound against MCF-7 and MDA-MB-231 breast cancer cells were 49.6 µM and 53.4 µM, respectively. The moderate cytostatic activity against estrogen-dependent cell line (MCF-7) had compounds **ST8** and **ST9**. While against estrogen-independent cell line (MDA-MB-231), mild anti-proliferative activity had compound **ST3** and compound **ST8**. A significant problem with many anticancer compounds is no selectivity for cancer cells. New 1,3,4-thiadiazole derivatives had weaker cytotoxic activity on normal cell lines (fibroblasts) than on breast cancer cell lines. Docking simulations demonstrated a possible multitarget mode of action for the synthesized compounds. Nevertheless, anticancer properties of **ST1**–**ST15** probably are mainly connected with the activities of Caspase 3 and Caspase 8 and activation of BAX proteins.

## Figures and Tables

**Table 1:** Docking simulations for activities of the **ST1**–**ST15** to Topoisomerase IIb (PDB: 3qx3), Caspase 3 (PDB: 1GFW), and Caspase 8 (PDB: 3KJN).

	Topoisomerase IIb (PDB: 3qx3)		Caspase 3 (PDB: 1GFW)		Caspase 8 (PDB: 3KJN)	
Compound	Estimated Free Energy of Binding, Kcal/mol	Estimated Inhibition Constant, Ki μM	Estimated Free Energy of Binding, Kcal/mol	Estimated Inhibition Constant, Ki μM	Estimated Free Energy of Binding, Kcal/mol	Estimated Inhibition Constant, Ki μM
ST1	−8.56	0.532	−7.16	5.60	−7.76	2.05
ST2	−8.82	0.344	−7.19	5.35	−7.83	1.83
ST3	−8.25	0.902	−6.71	12.00	−7.52	3.07
ST4	−8.36	0.742	−6.95	8.07	−7.52	3.08
ST5	−8.11	1.13	−6.71	12.00	−7.14	5.83
ST6	−7.94	1.52	−6.68	12.78	−7.22	5.09
ST7	−8.34	0.767	−6.80	10.35	−7.60	2.70
ST8	−8.36	0.749	−7.01	7.31	−7.62	2.58
ST9	−8.05	1.25	−6.31	23.83	−7.38	3.92
ST10	−8.00	1.37	−6.85	9.56	−7.34	4.16
ST11	−7.99	1.40	−7.21	5.17	−7.41	3.68
ST12	−7.58	2.78	−6.27	25.34	−7.29	4.51
ST13	−7.23	5.04	−6.02	38.69	−6.70	12.30
ST14	−7.24	4.90	−5.85	51.55	−6.82	10.03
ST15	−8.61	0.484	−7.65	2.49	−8.28	0.852
Etoposide	−11.84	0.002				
MSI			−8.21	0.967		
B93					−8.17	1.03

**Table 2:** Docking simulations for activities of the **ST1**–**ST15** to Bcl-xl (PDB: 2YXJ), Bcl2 (PDB: 2W3L), and BAX (PDB: 1F16).

	Bcl-xl (PDB: 2YXJ)		Bcl2 (PDB: 2W3L)		BAX (PDB: 1F16)	
Compound	Estimated Free Energy of Binding, Kcal/mol	Estimated Inhibition Constant, Ki μM	Estimated Free Energy of Binding, Kcal/mol	Estimated Inhibition Constant, Ki μM	Estimated Free Energy of Binding, Kcal/mol	Estimated Inhibition Constant, Ki μM
ST1	−6.73	11.62	−6.20	28.77	−6.20	28.51
ST2	−6.71	12.14	−6.01	39.52	−5.91	46.80
ST3	−6.66	13.06	−5.88	49.04	−7.68	2.36
ST4	−6.32	23.44	−5.79	57.04	−7.18	5.41
ST5	−5.94	44.43	−5.66	71.13	−7.18	5.43
ST6	−6.08	34.90	−5.54	86.20	−7.13	5.91
ST7	−6.44	19.03	−5.95	43.85	−6.11	32.96
ST8	−7.09	6.37	−5.51	91.92	−7.68	2.34
ST9	−5.95	43.53	−5.34	121.27	−7.16	5.63
ST10	−6.04	37.33	−5.91	46.80	−7.43	3.56
ST11	−6.17	29.96	−5.90	46.99	−7.09	6.33
ST12	−5.73	63.25	−5.43	105.37	−6.78	10.73
ST13	−5.39	112.63	−5.78	57.96	−5.56	83.93
ST14	−6.05	36.67	−5.58	81.50	−5.29	132.02
ST15	−7.03	6.99	−6.39	20.77	−6.30	23.95
N3C	−13.62	103.7 × 10^−6^	−	−	−	−
DRO	−	−	−9.71	0.07598		
Zinc 14750348	−	−	−	−	−8.13	1.09

**Table 3:** Prediction of the molecular descriptors (Lipinski rules of five) for compounds **ST1**–**ST15** by Swissadme.

Compounds	MW ^a^ g/mol	MLogP ^b^	RB ^c^	HBA ^d^	HBD ^e^	TPSA ^f^ Å^2^	LogS (ESOL) ^g^	Violations ^h^
ST1	317.79	3.16	4	3	1	75.28	−4.98	0
ST2	317.79	3.16	4	3	1	75.28	−4.98	0
ST3	317.79	3.16	4	3	1	75.28	−4.98	0
ST4	301.34	3.03	4	4	1	75.28	−4.54	0
ST5	301.34	3.03	4	4	1	75.28	−4.54	0
ST6	301.34	3.03	4	4	1	75.28	−4.54	0
ST7	313.37	2.33	5	4	1	84.51	−4.45	0
ST8	313.37	2.33	5	4	1	84.51	−4.45	0
ST9	313.37	2.33	5	4	1	84.51	−4.45	0
ST10	351.35	3.25	5	6	1	75.28	−5.21	0
ST11	351.35	3.25	5	6	1	75.28	−5.21	0
ST12	351.35	3.25	5	6	1	75.28	−5.21	0
ST13	328.35	1.6	5	5	1	121.10	−4.77	0
ST14	328.35	1.6	5	5	1	121.10	−4.77	0
ST15	328.35	1.6	5	5	1	121.10	−4.77	0

^a^ Molecular weight; ^b^ lipophilicity (MLog Po/w); ^c^ number of rotatable bond; ^d^ number of hydrogen bond acceptor; ^e^ number of hydrogen bond donor; ^f^ topological polar surface area; ^g^ estimating aqueous solubility; ^h^ violations from Lipinski’s rule.

**Table 4:** ADMET profile calculations for **ST1**–**ST15**.

Compounds	Hum In Ab ^a^	Caco-2 P ^b^	CYP Inh Prom ^c^	Carcin ^d^	AOT ^e^	RAT ^f^
ST1	+	1.8519	High	Non	III	2.3535
ST2	+	1.6696	High	Non	III	2.3879
ST3	+	1.6696	High	Non	III	2.3879
ST4	+	1.7663	High	Non	III	2.4976
ST5	+	1.5970	High	Non	III	2.5158
ST6	+	1.5970	High	Non	III	2.5158
ST7	+	1.7935	High	Non	III	2.2412
ST8	+	1.6903	High	Danger	III	2.1570
ST9	+	1.6732	High	Danger	III	2.2011
ST10	+	1.6239	High	Non	III	2.6075
ST11	+	1.4616	High	Non	III	2.6445
ST12	+	1.4616	High	Non	III	2.6445
ST13	+	1.4822	High	Danger	III	2.1243
ST14	+	1.3275	High	Danger	III	2.3265
ST15	+	1.3275	High	Danger	III	2.3265

^a^ Human intestinal absorption, ^b^ Caco-2 permeability LogPapp, cm/s, ^c^ CYP inhibitory promiscuity, ^d^ carcinogenicity, ^e^ acute oral toxicity, ^f^ rat acute toxicity LD_50_ mol/kg.

## Data Availability

The details of the data supporting the report results in this research were included in the paper and Supplementary Materials.

## Supplementary Materials

The following supporting information can be downloaded at: https://www.mdpi.com/article/10.3390/molecules27061814/s1, ^1^H NMR, ^13^C NMR spectra.

## Sample Availability

Samples of the compounds are/ are not available from the authors upon request.
