# Supplementary material for: New 1,3,4-Thiadiazole Derivatives with Anticancer Activity
Source: Molecules. 2022 Mar 10;27(6):1814. doi: 10.3390/molecules27061814 (PMC8955053; doi:10.3390/molecules27061814)
Supplement: Supplementary file 1 [file molecules-27-01814-s001.zip › molecules-1613128-supplementary.pdf]

molecules

MDPI

Supplementary Information

New 1,3,4-thiadiazole derivatives with cytotoxic activity

New 1,3,4-Thiadiazole Derivatives with Anticancer Activity

Sara Janowska [1], Dmytro Khylyuk [1], Anna Bielawska [2], Anna Szymanowska [2], Agnieszka Gornowicz [2], Krzysztof Bielawski [3], Jarosław Noworól [4], Sławomir Mandziuk [5] and Monika Wujec [1,*]

[1] Department of Organic Chemistry, Faculty of Pharmacy, Medical University, 4a Chodzki Street, 20-093 Lublin, Poland; sarajanowska@umlub.pl (S.J.); dmytrokhylyuk@umlub.pl (D.K.)
[2] Department of Biotechnology, Faculty of Pharmacy, Medical University of Bialystok, Kilinskiego 1 Street, 15-089 Bialystok, Poland; anna.bielawska@umb.edu.pl (A.B.); anna.szymanowska@umb.edu.pl (A.S.); agnieszka.gornowicz@umb.edu.pl (A.G.)
[3] Department of Synthesis and Technology of Drugs, Faculty of Pharmacy, Medical University of Bialystok, Kilinskiego 1 Street, 15-089 Bialystok, Poland; krzysztof.bielawski@umb.edu.pl
[4] Health Care Institute, State Higher School of Technology and Economics in Jarosław, Czarnieckiego 16 Street, 37-500 Jarosław, Poland; jaroslaw.noworol@pwste.edu.pl
[5] Department of Pneumology, Oncology and Allergology, Medical University of Lublin, 8 Jaczewskiego Street, 20–090 Lublin, Poland; slawomir.mandziuk@umlub.pl
* Correspondence: monika.wujec@umlub.pl

Table of contents:

^{1}H NMR spectra of thiosemicarbazide derivatives……………………………………………..…………………………………2
^{1}H NMR spectra of 1,3,4-thiadiazole derivatives………………………………………………………………………………..…16
^{13}C NMR spectra of thiosemicarbazide derivatives……………………..………………………………………………………....31
^{13}C NMR spectra of 1,3,4-thiadiazole derivatives…………………………………………………………………………….......46

1. H NMR spectra of thiosemicarbazide derivatives:

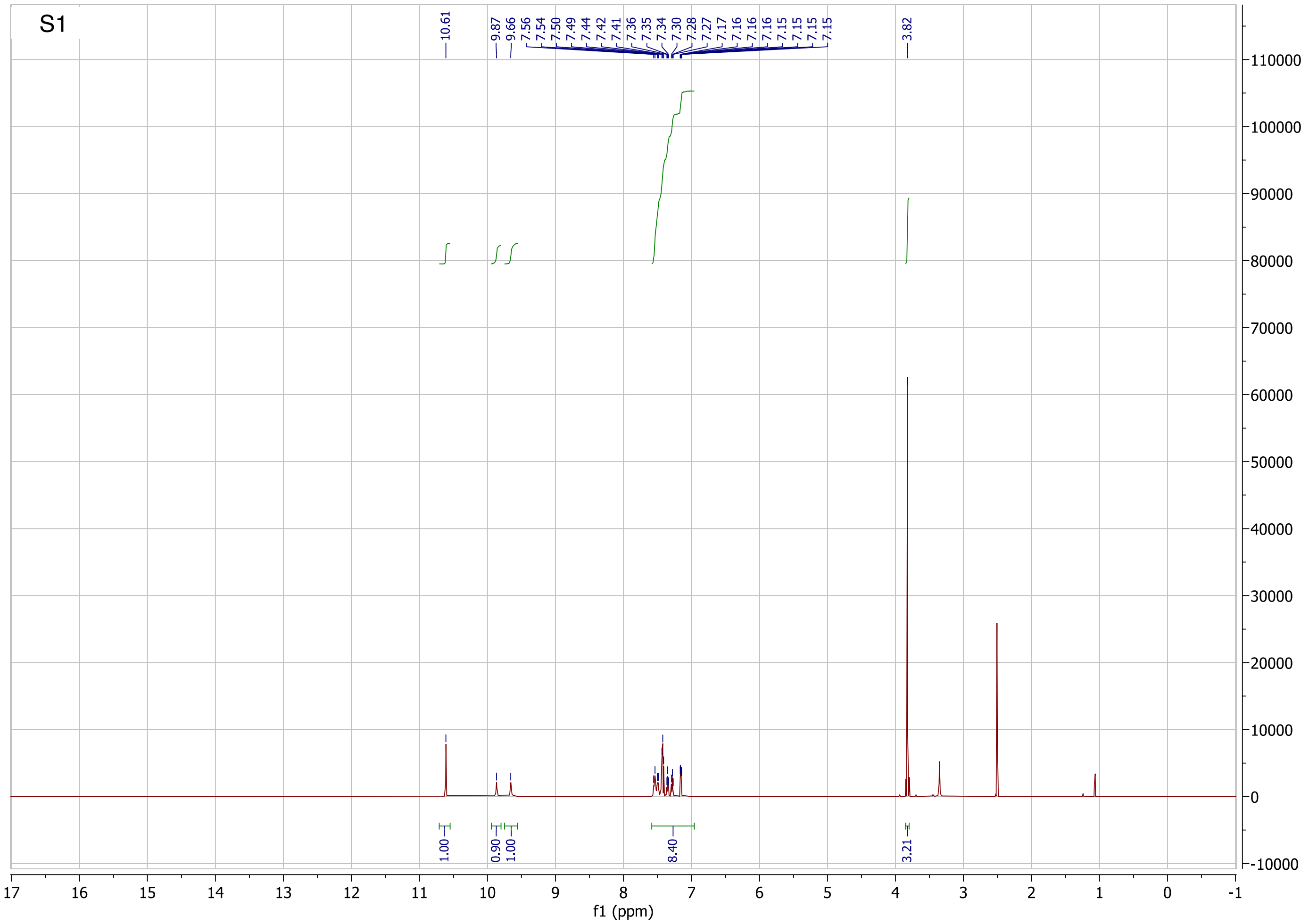

Figure S1. The ^{1}H NMR of compound S1.

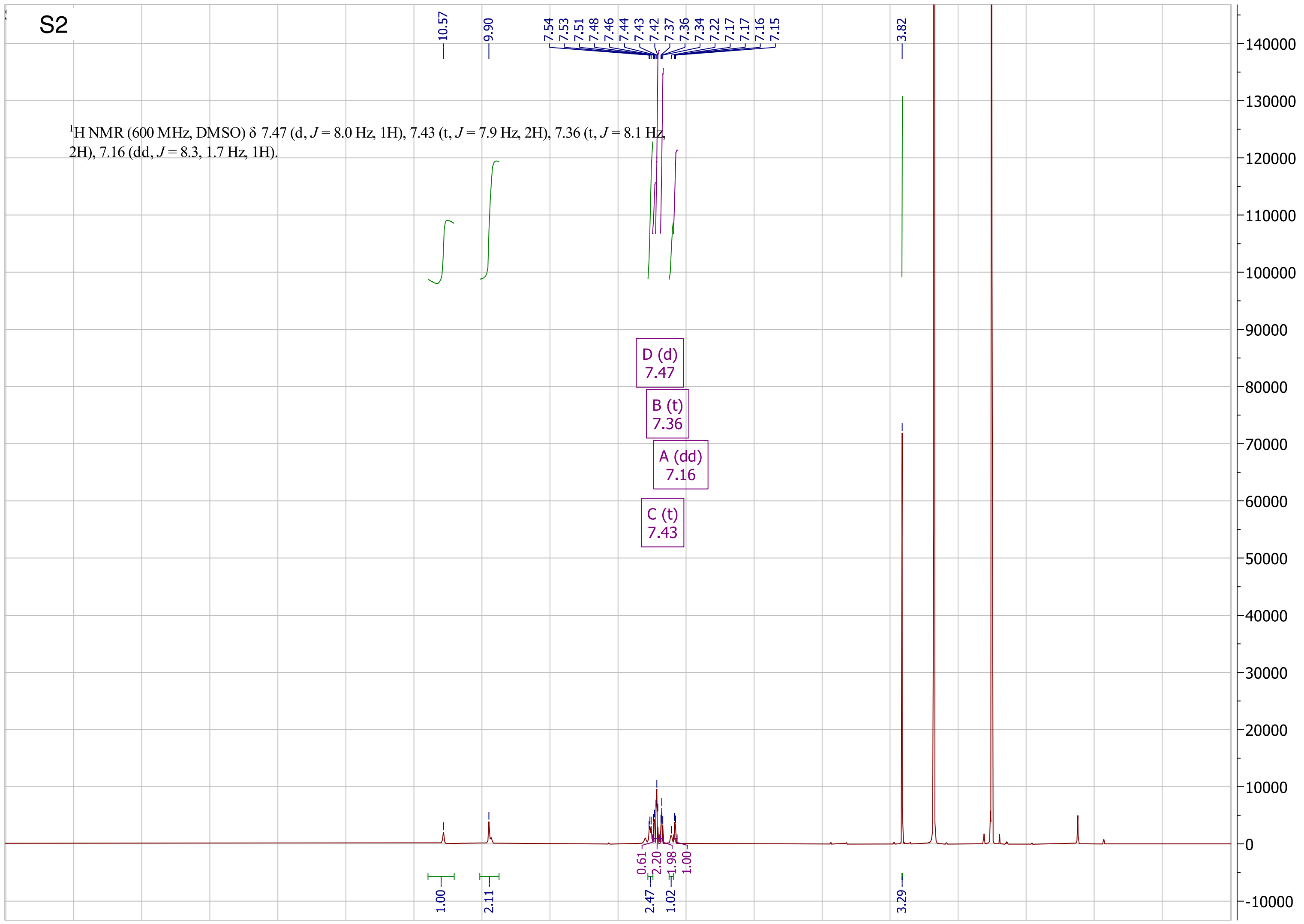

Figure S2. The ¹H NMR of compound S2.

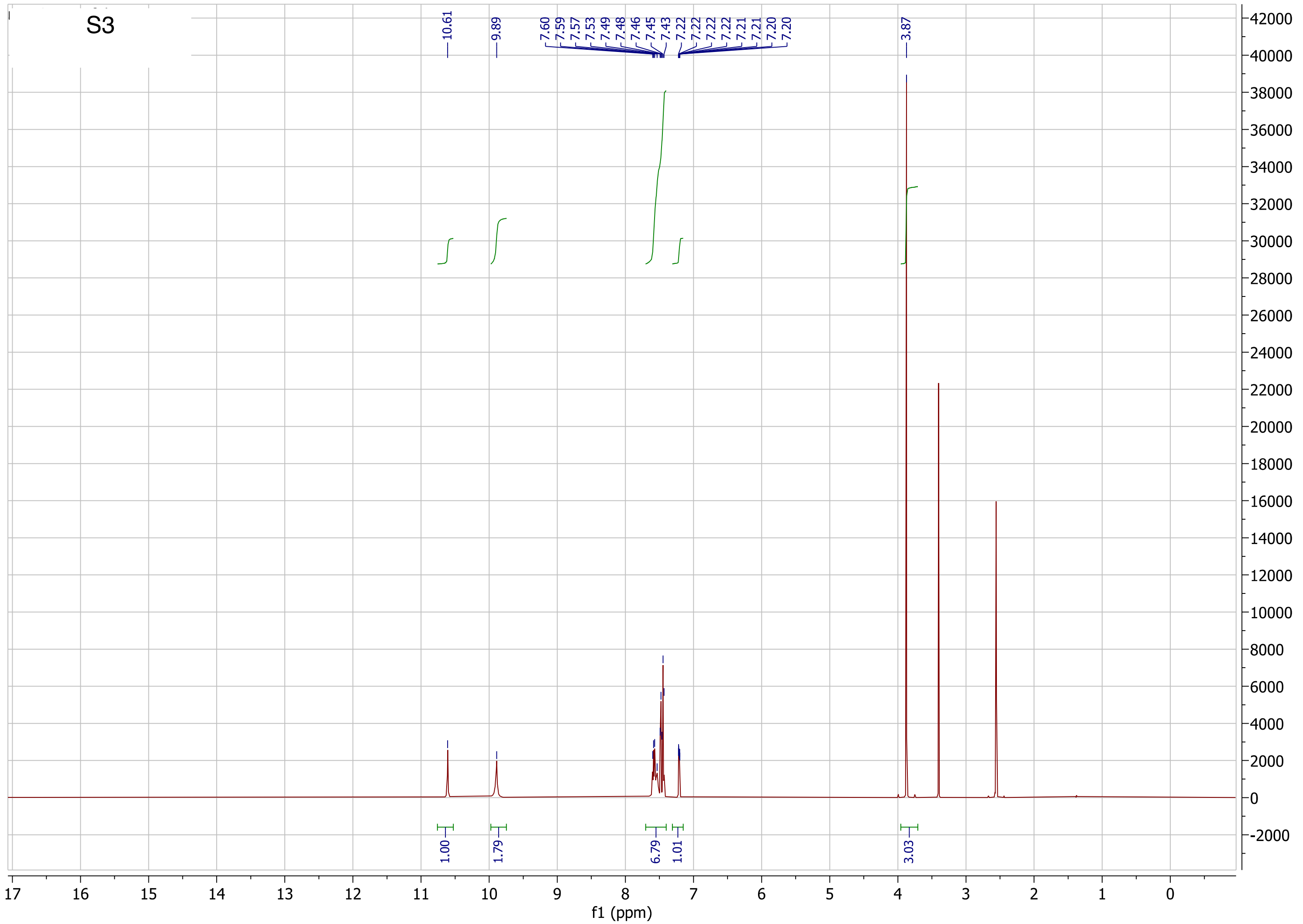

Figure S3. The ¹H NMR of compound S3.

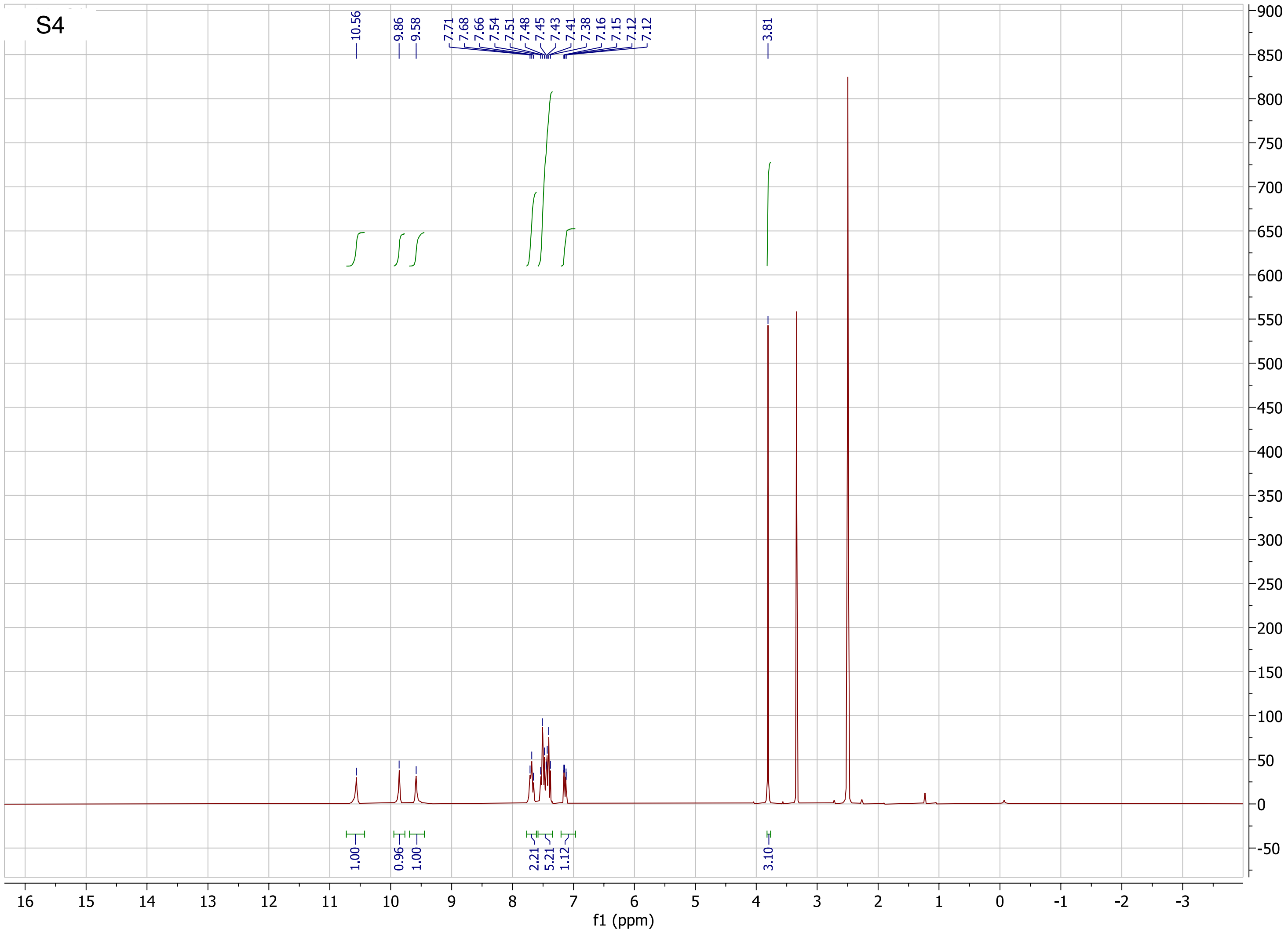

Figure S4. The ^{1}H NMR of compound S4.

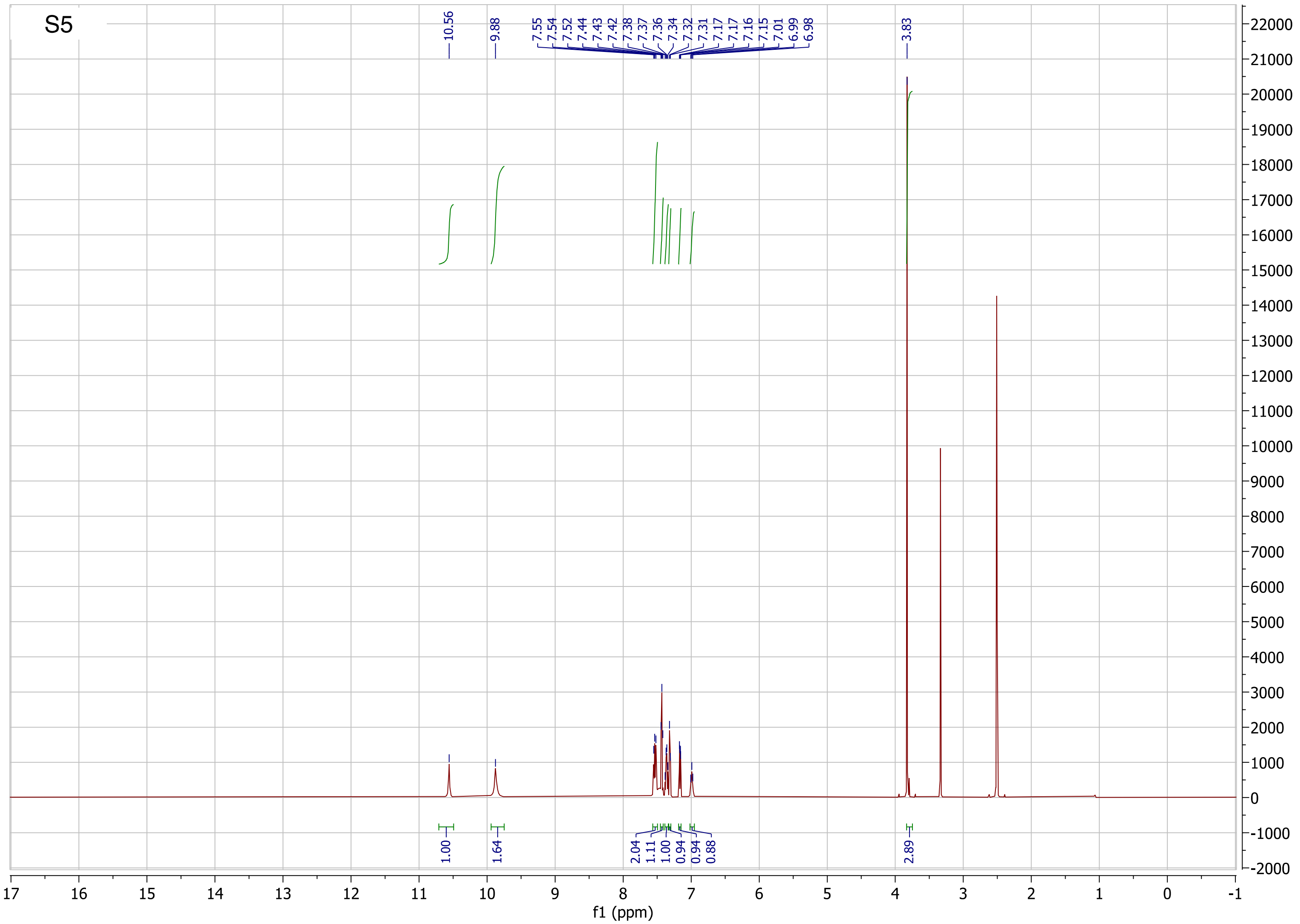

Figure S5. The ^{1}H NMR of compound S5.

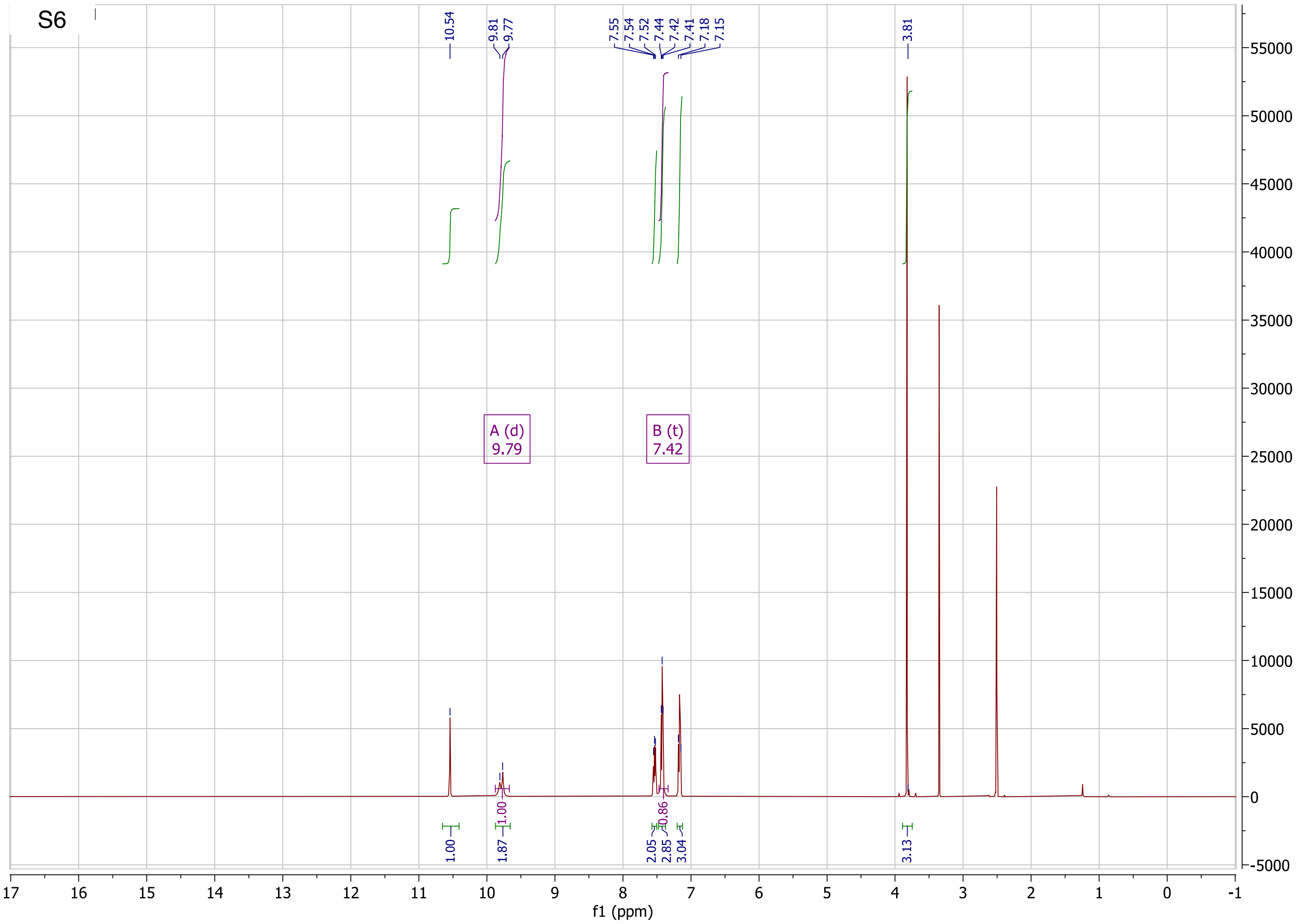

Figure S6. The ^{1}H NMR of compound S6.

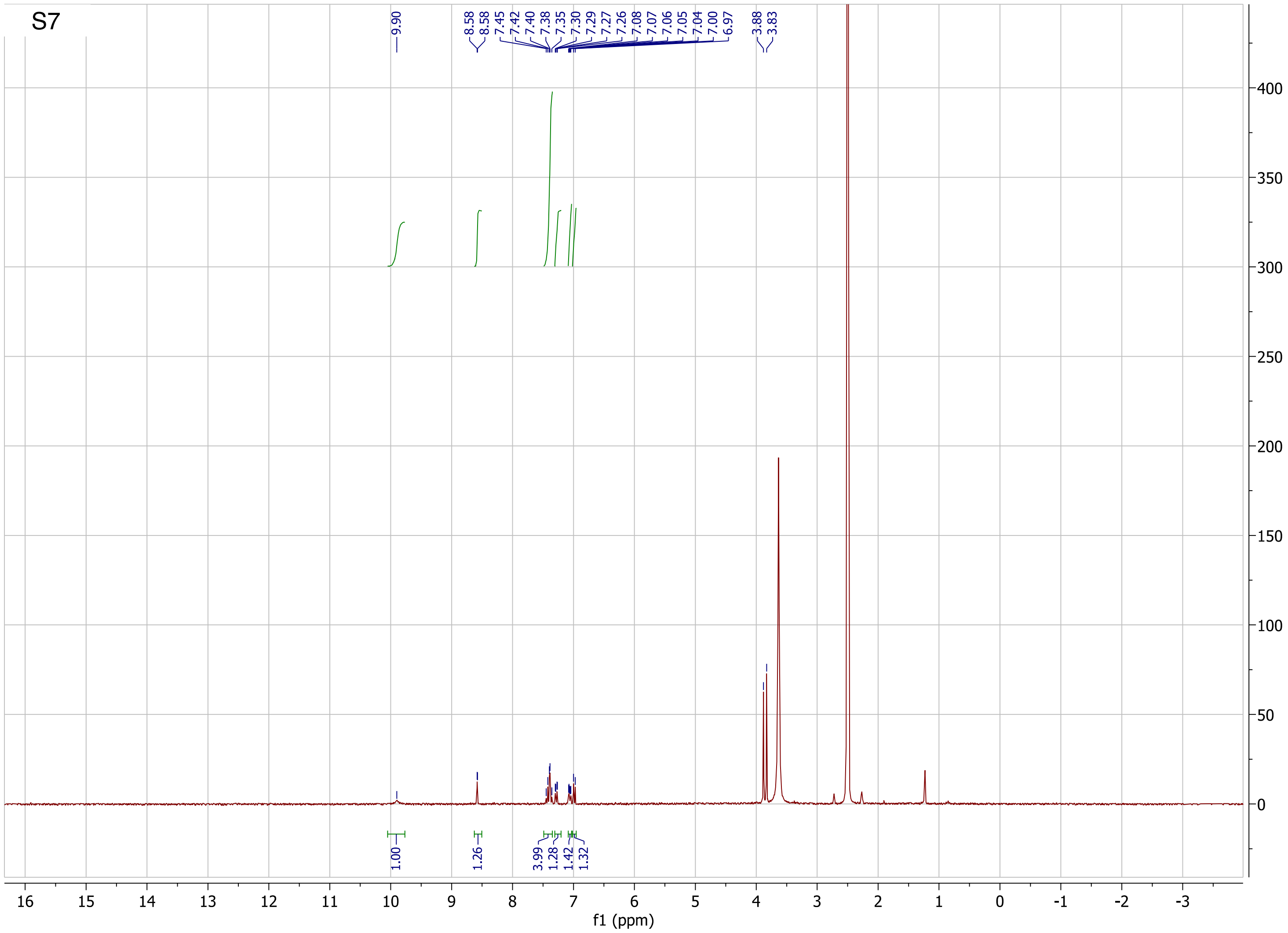

Figure S7. The ^{1}H NMR of compound S7.

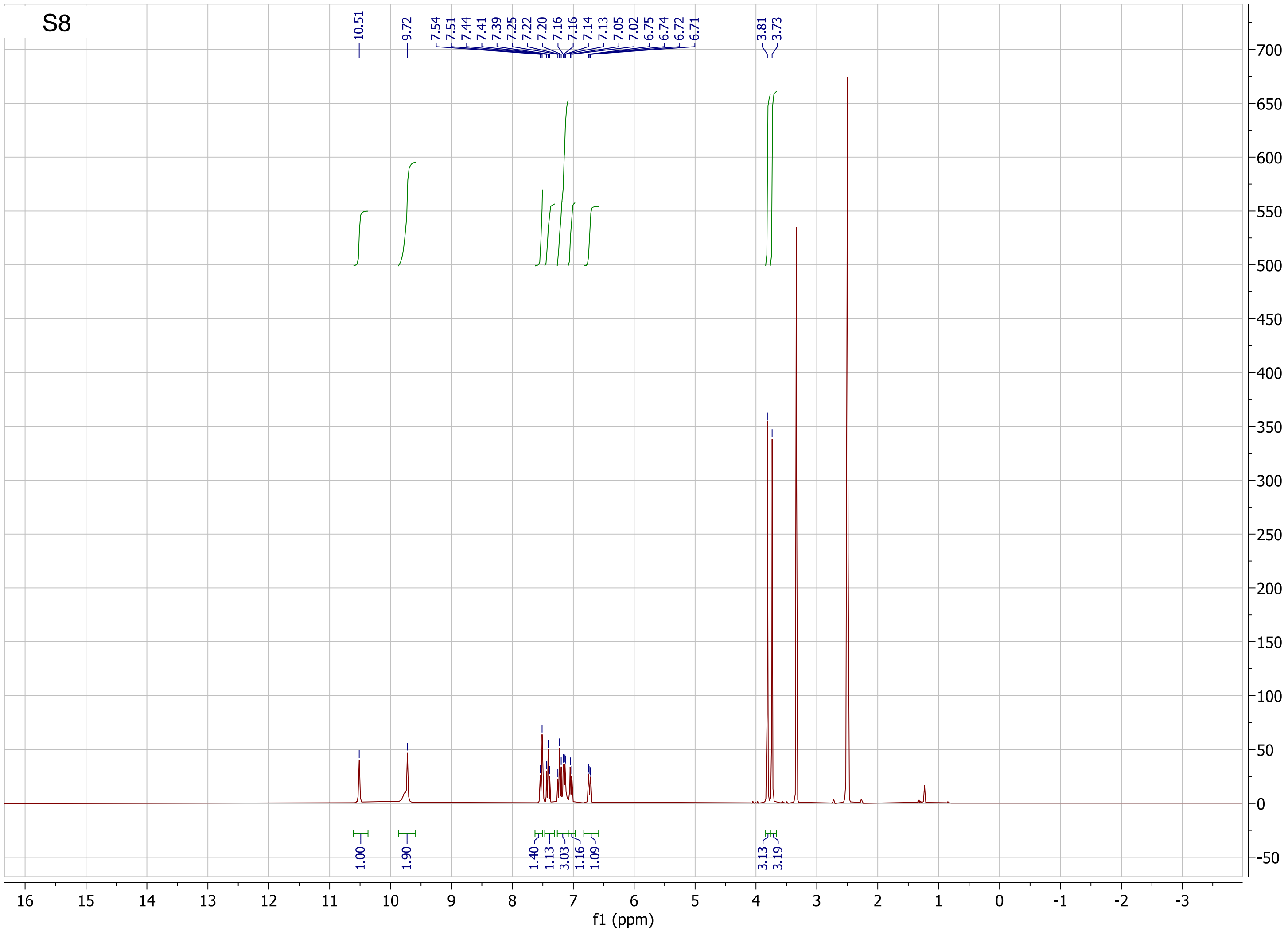

Figure S8. The ^{1}H NMR of compound S8.

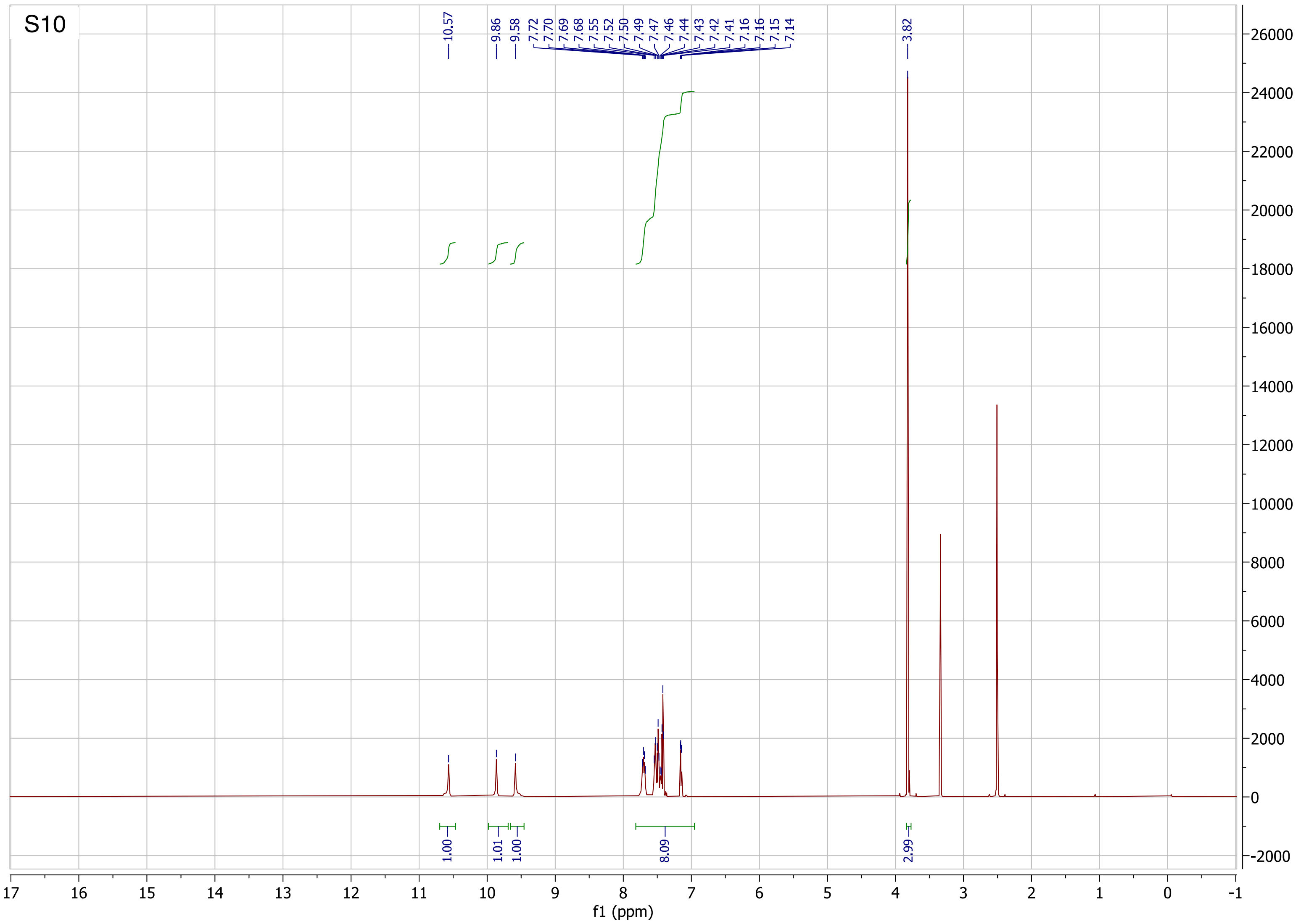

Figure S9. The ^{1}H NMR of compound S10.

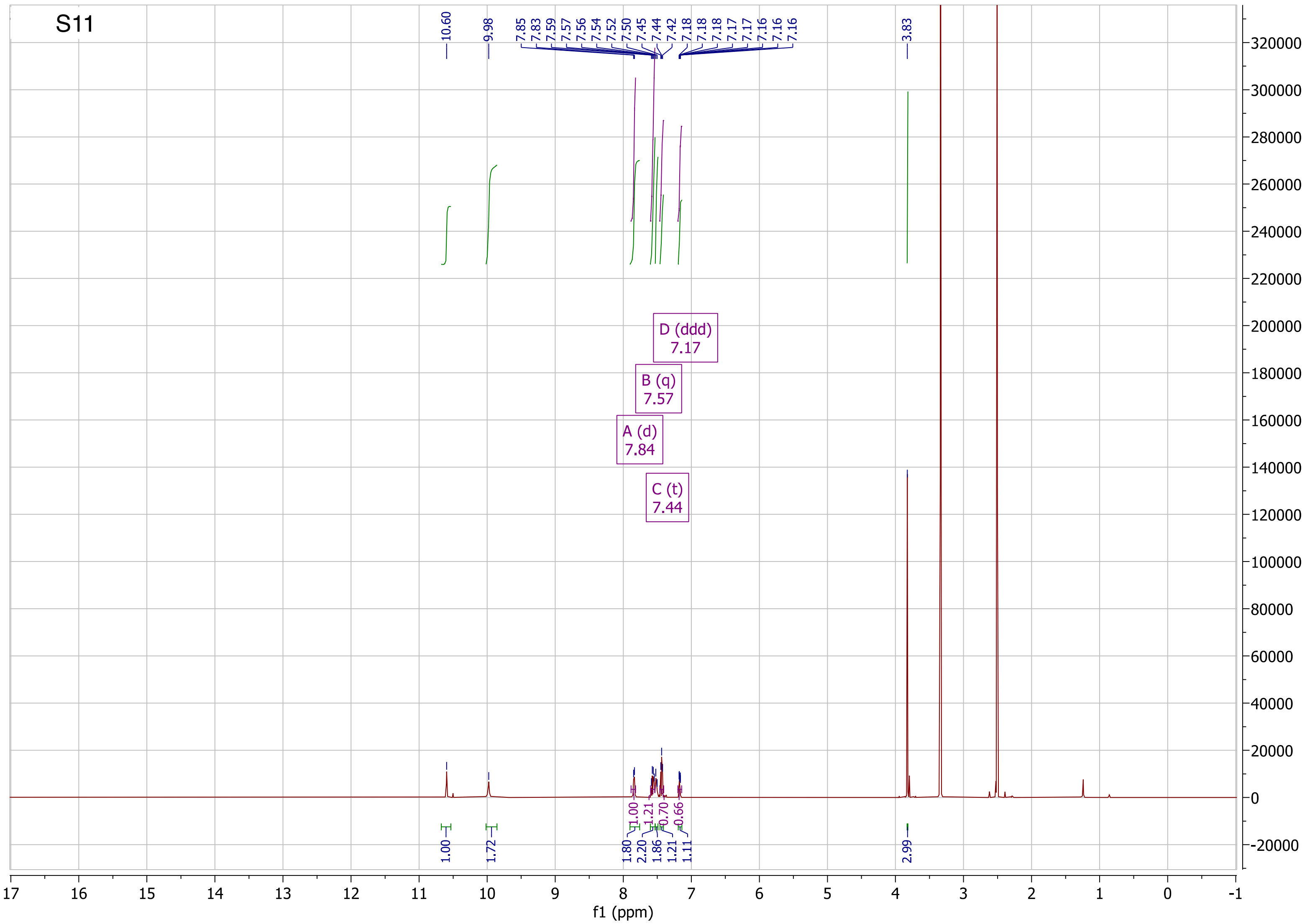

Figure S10. The ^{1}H NMR of compound S11.

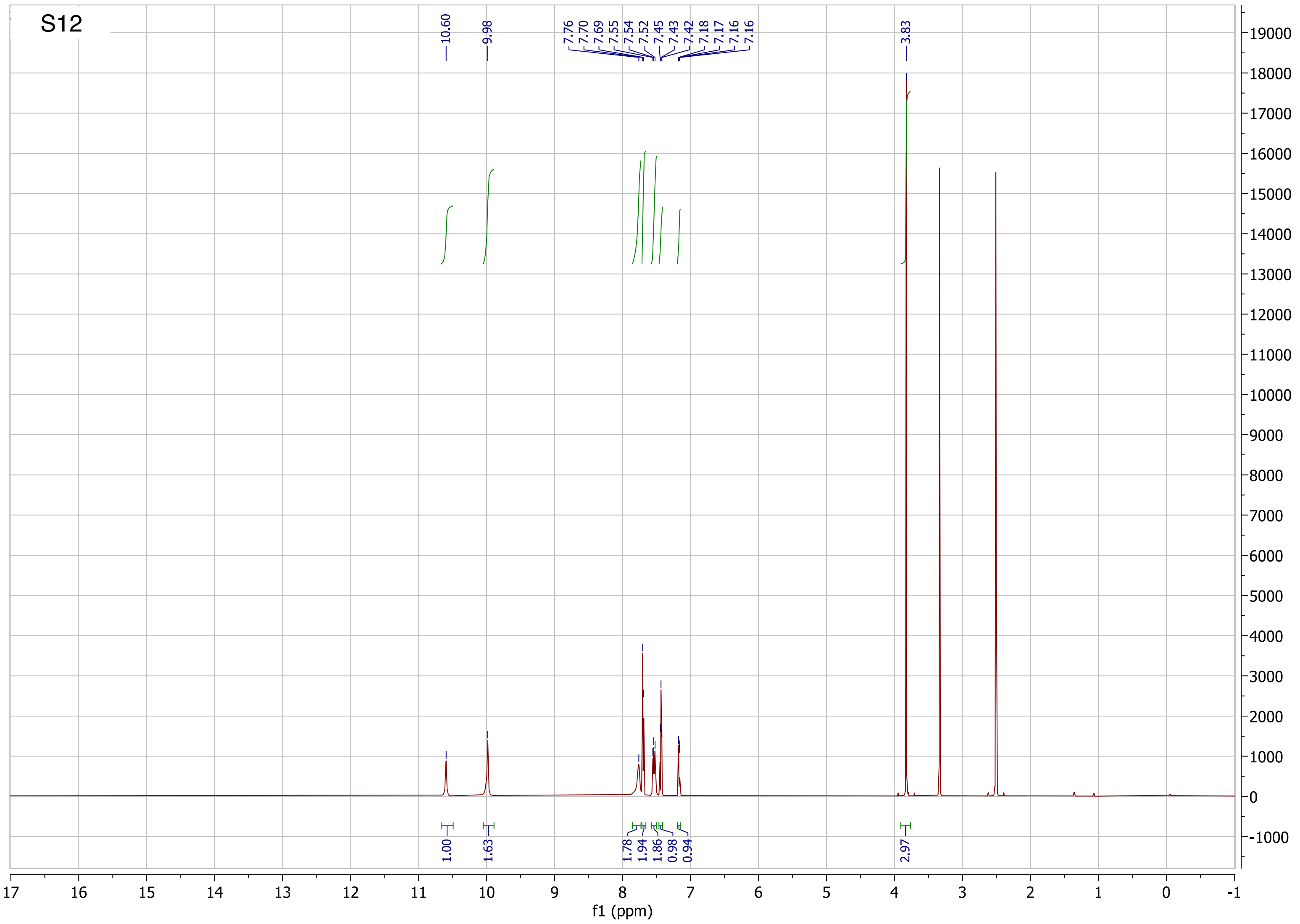

Figure S11. The ^{1}H NMR of compound S12.

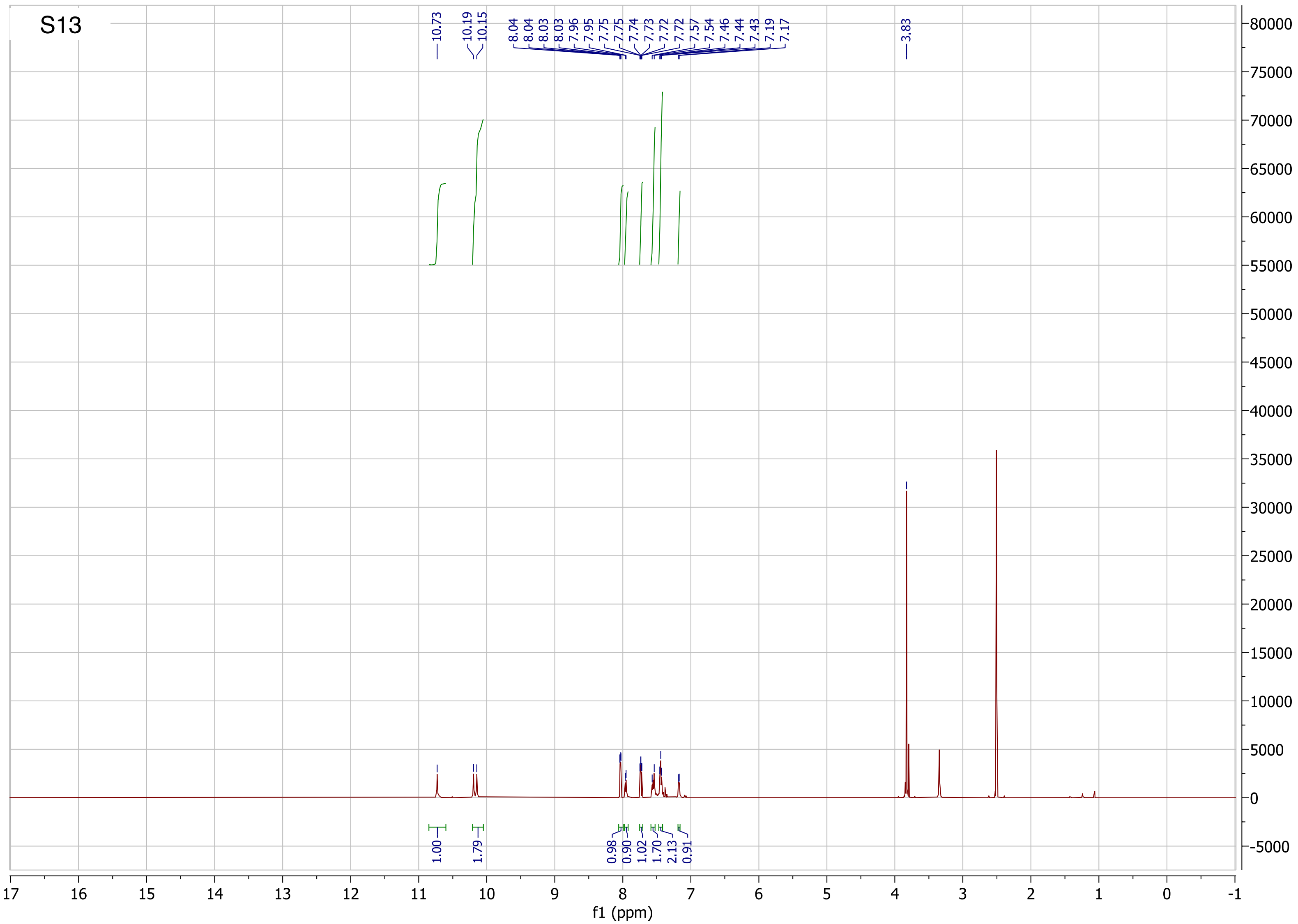

Figure S12. The ^{1}H NMR of compound S13.

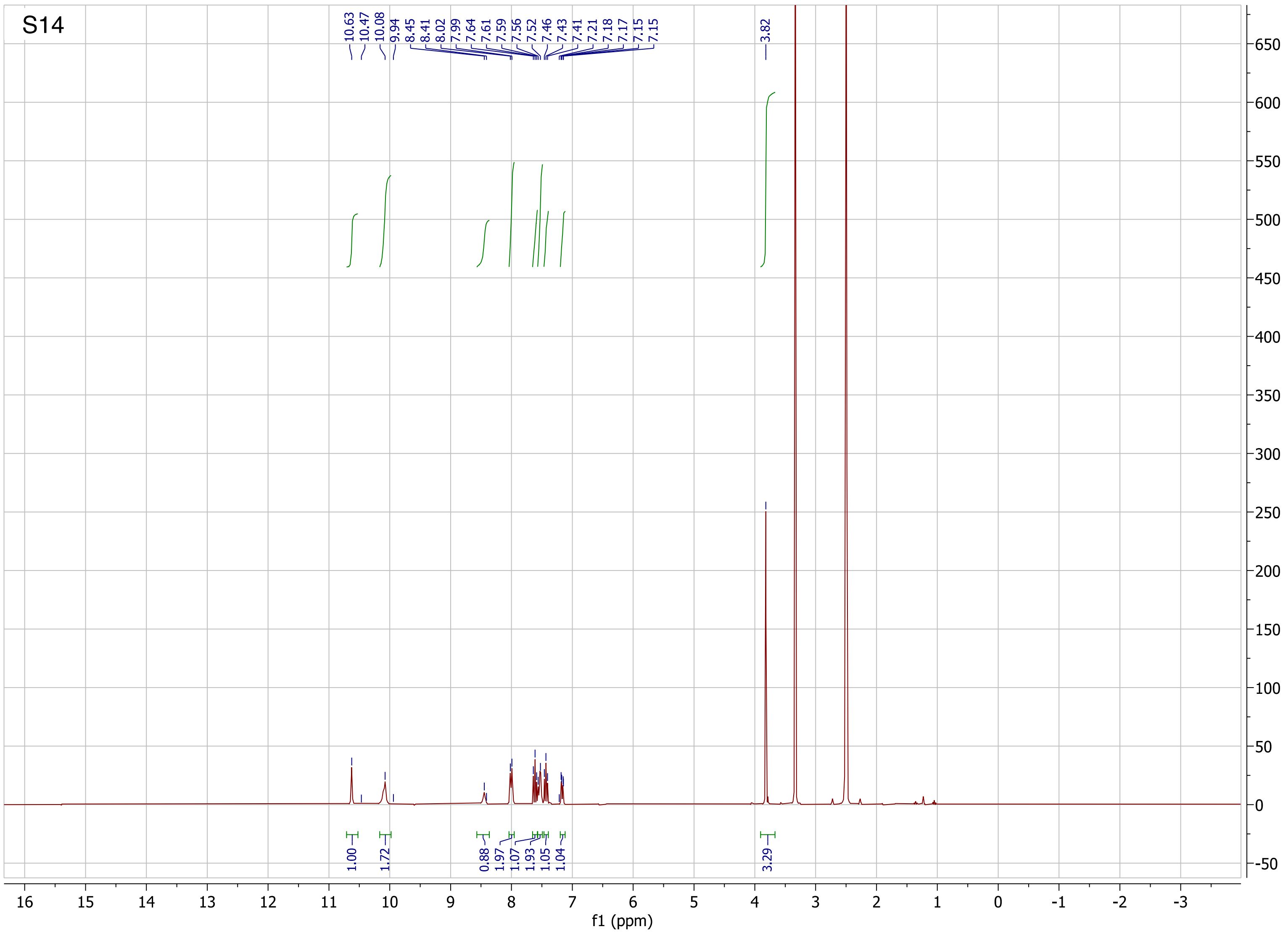

Figure S13. The ^{1}H NMR of compound S14.

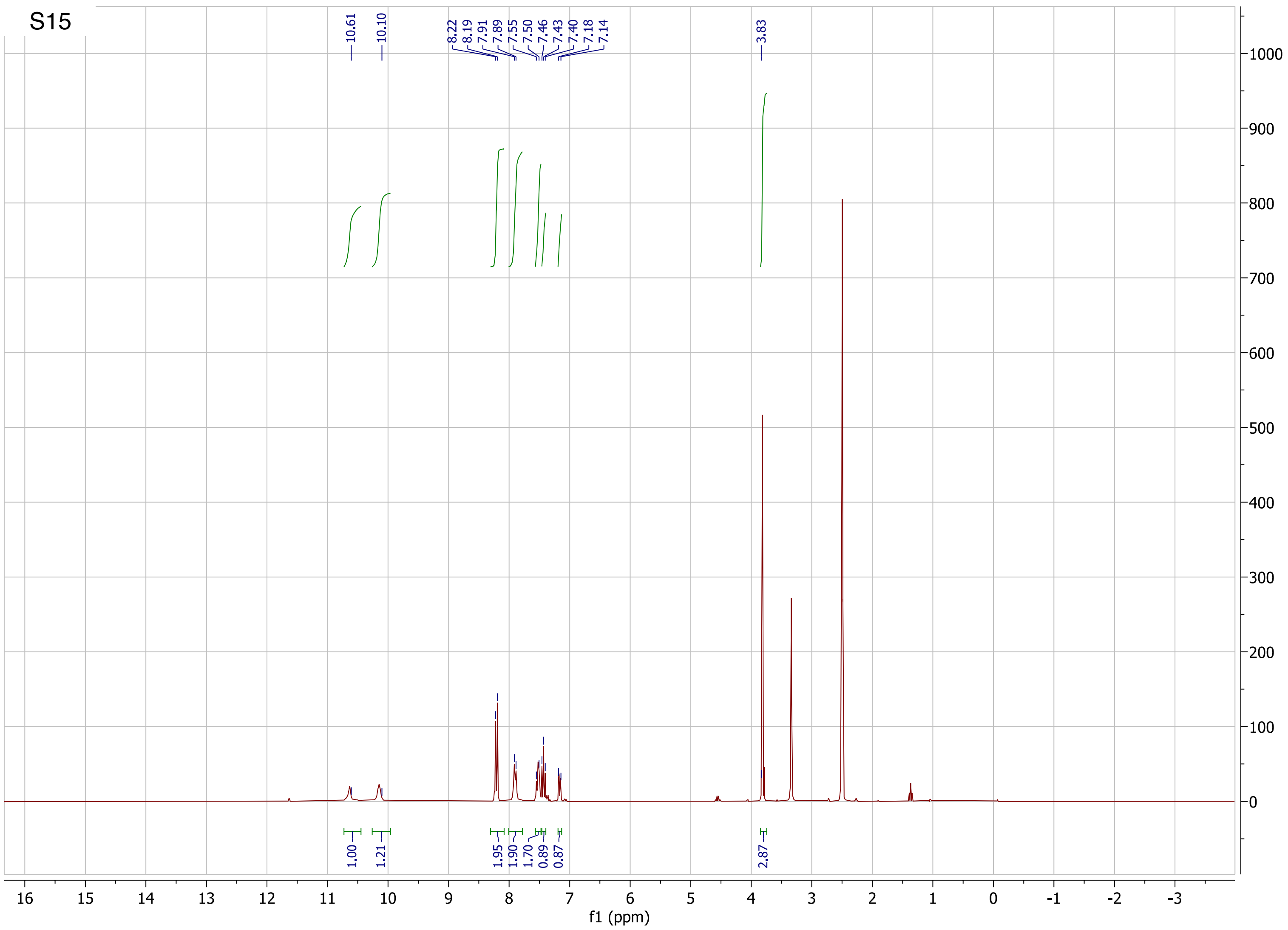

Figure S14. The ^{1}H NMR of compound S15.

$^{1.}$ H NMR spectra of 1,3,4-thiadiazole derivatives:

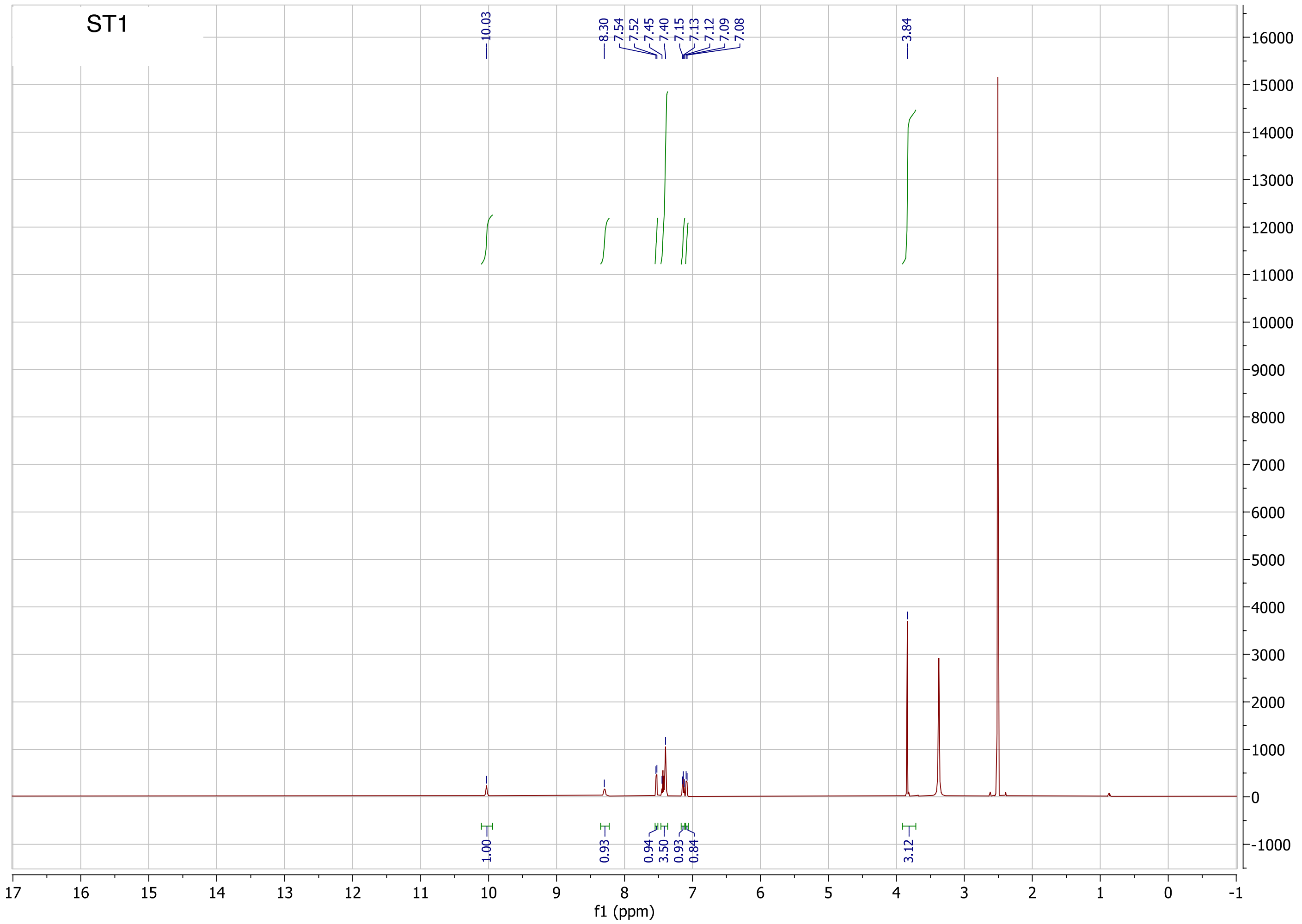

Figure S15. The ^{1}H NMR of compound ST1.

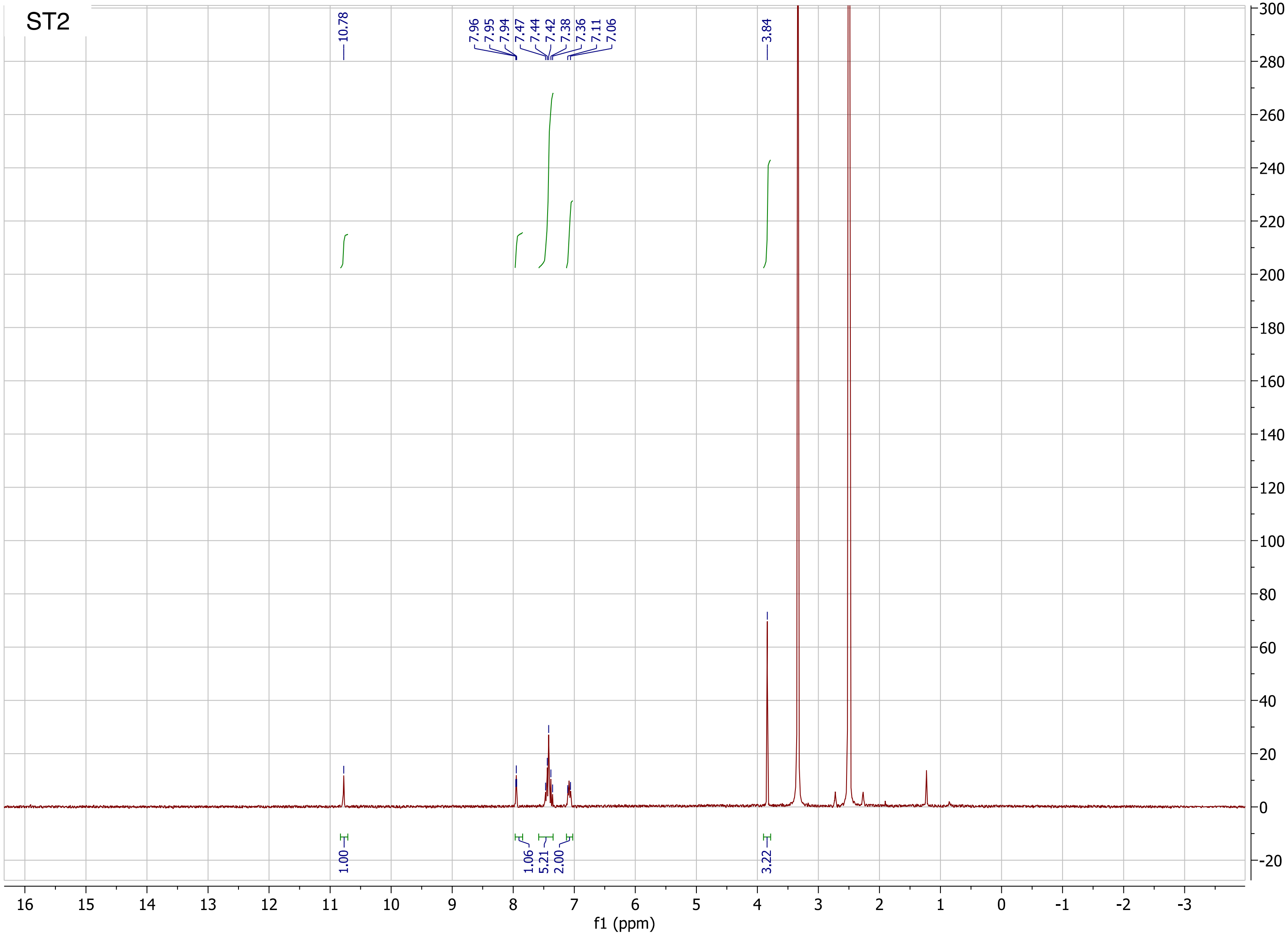

Figure S16. The ^{1}H NMR of compound ST2.

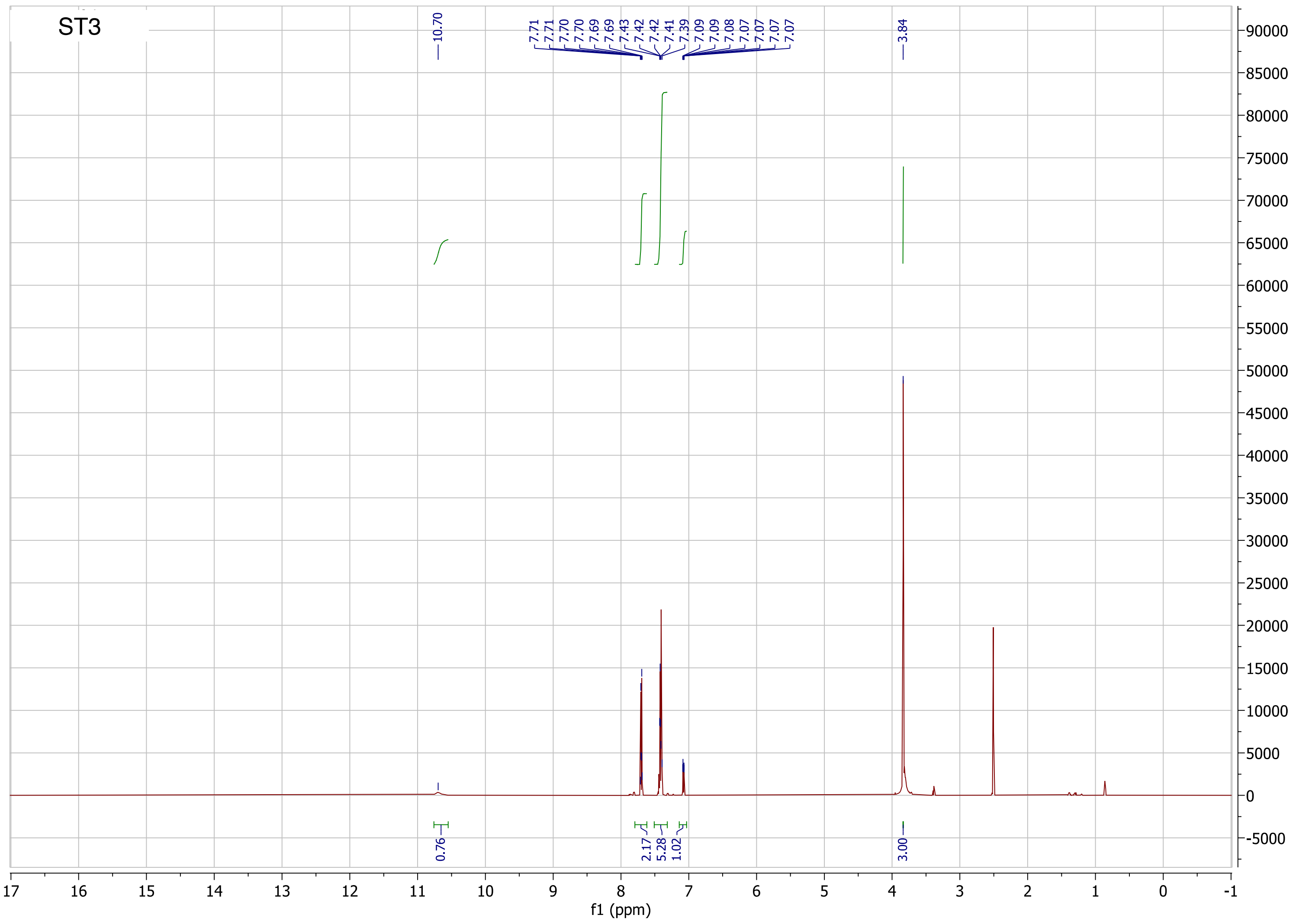

Figure S17. The ^{1}H NMR of compound ST3.

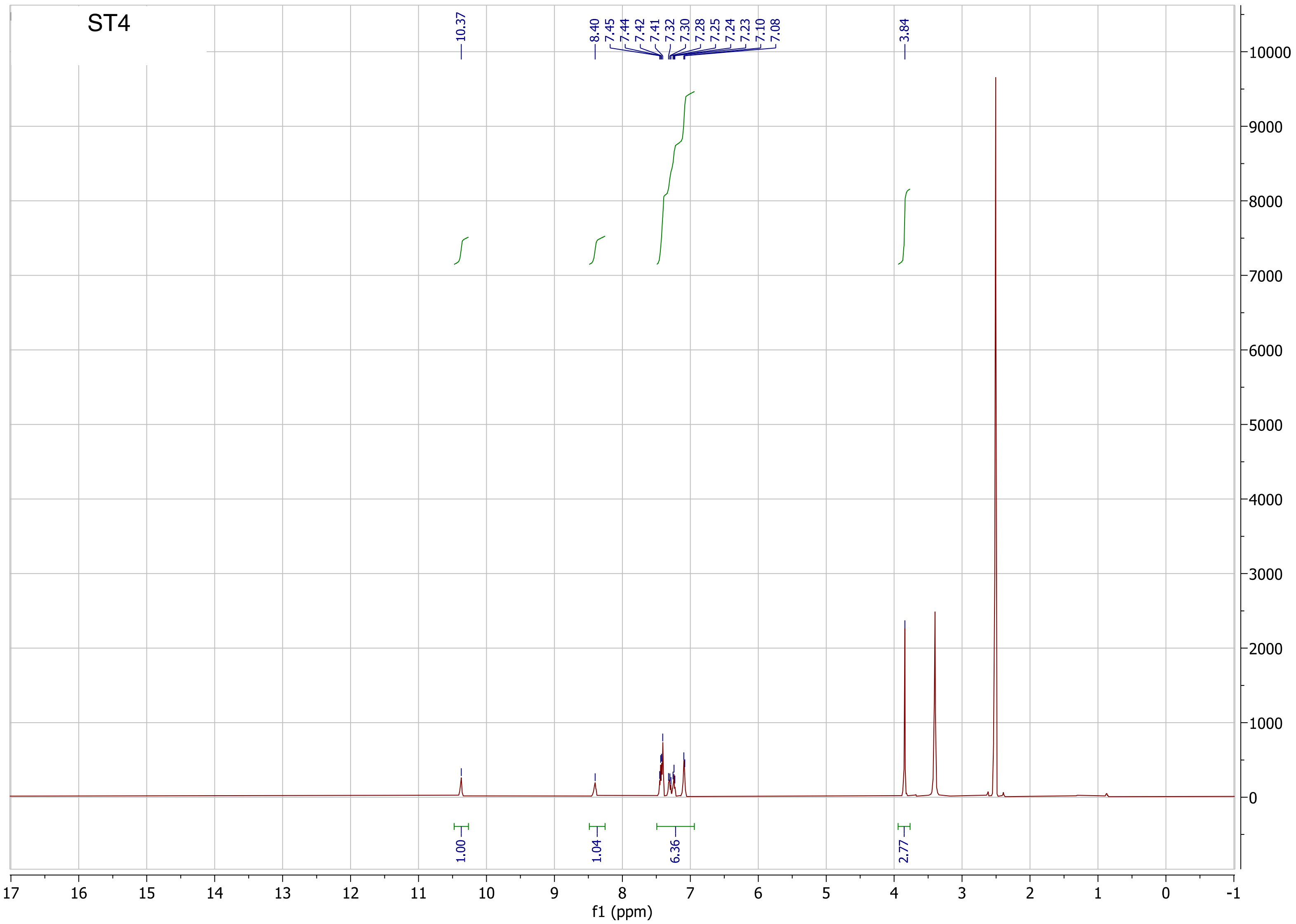

Figure S18. The ^{1}H NMR of compound ST4.

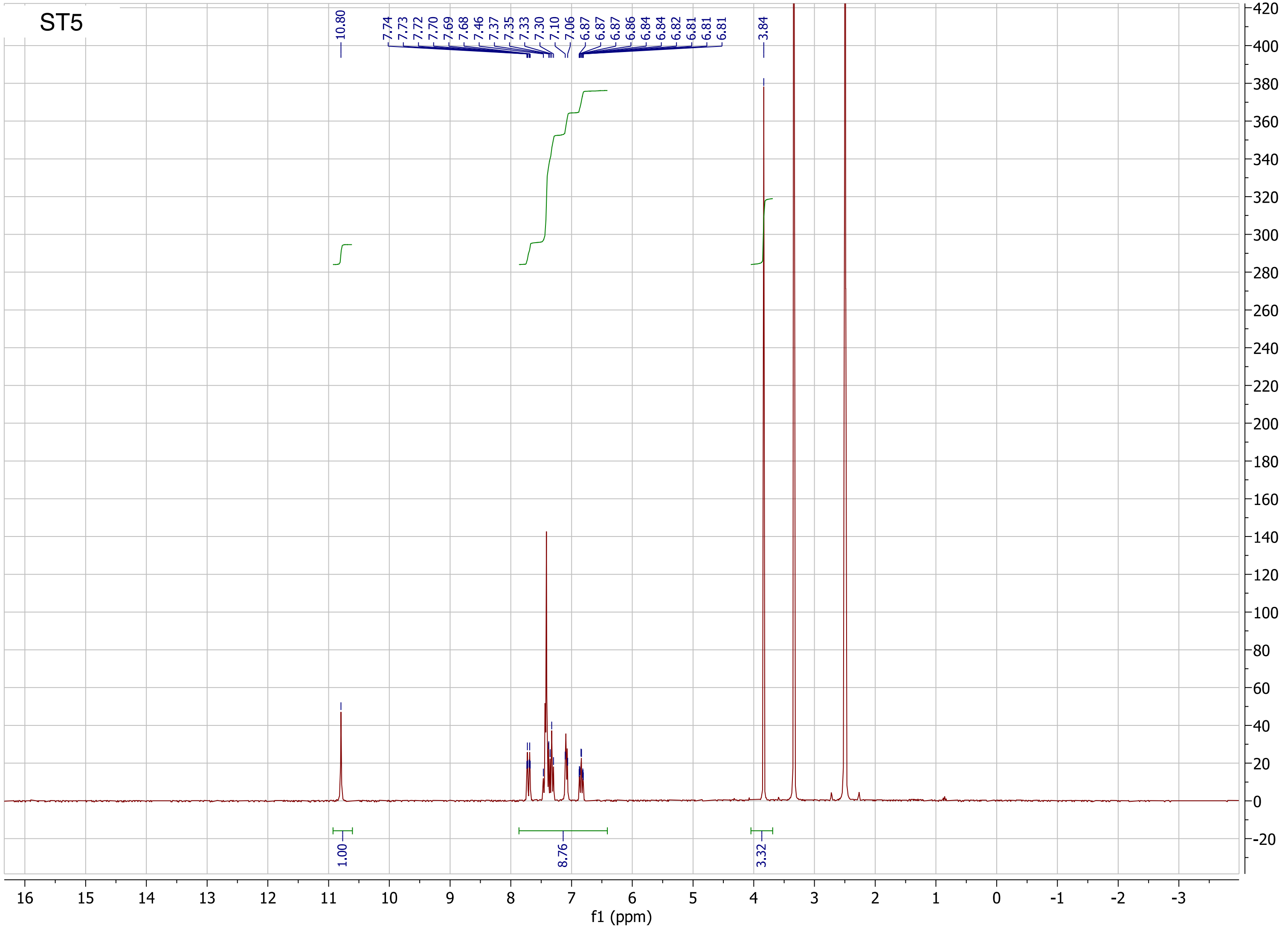

Figure S19. The ^{1}H NMR of compound ST5.

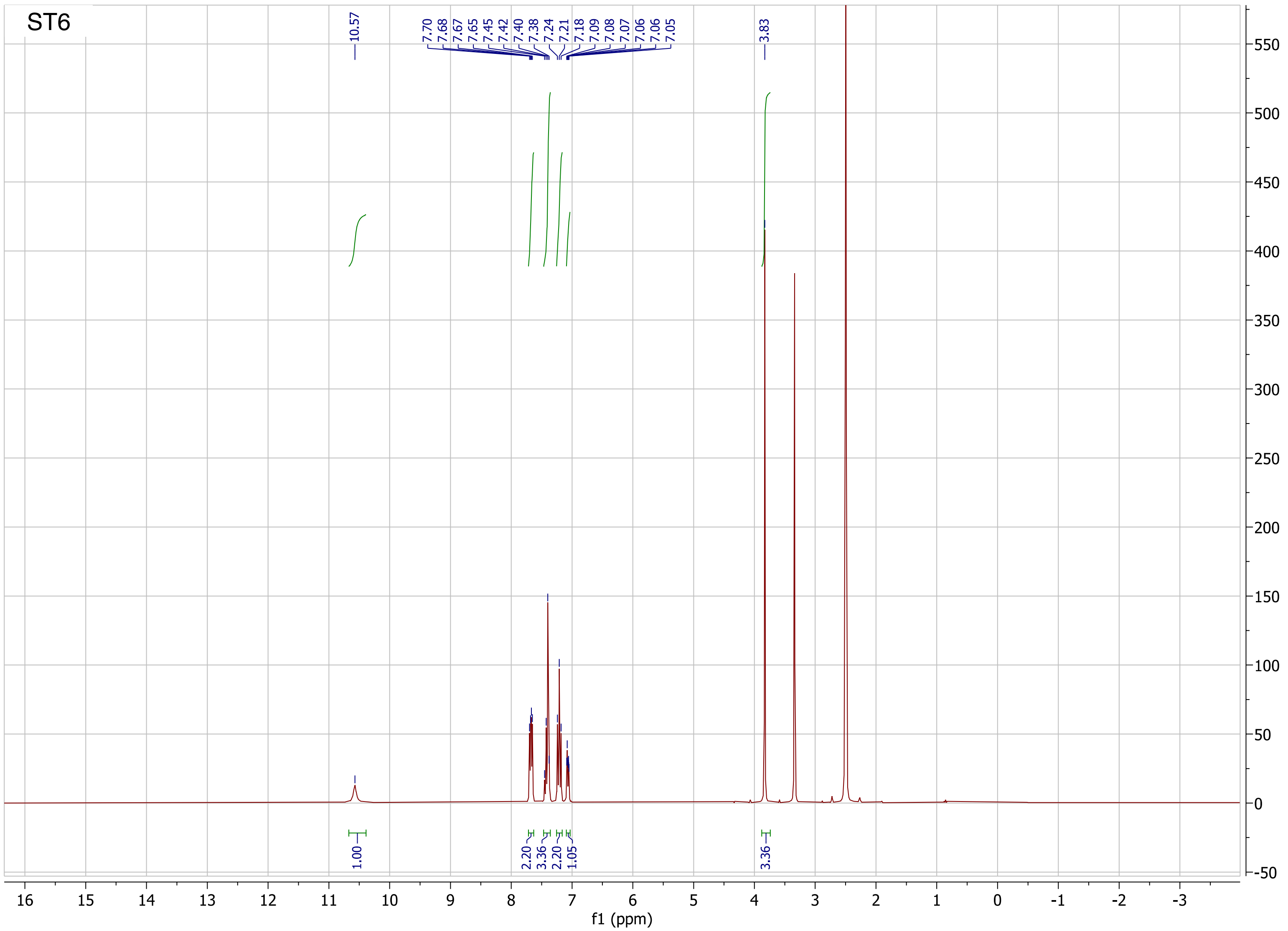

Figure S20. The ^{1}H NMR of compound ST6.

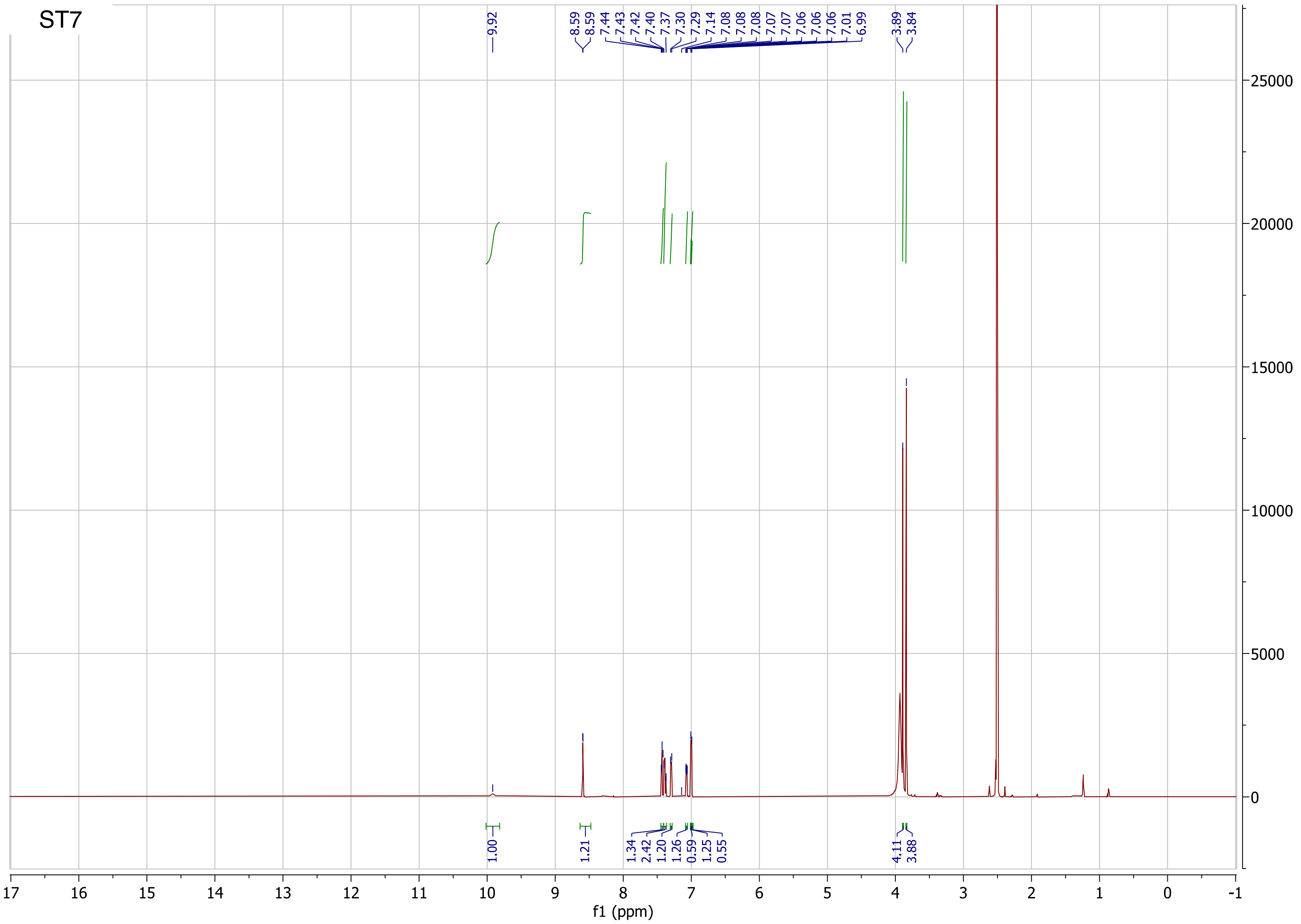

Figure S21. The ^{1}H NMR of compound ST7.

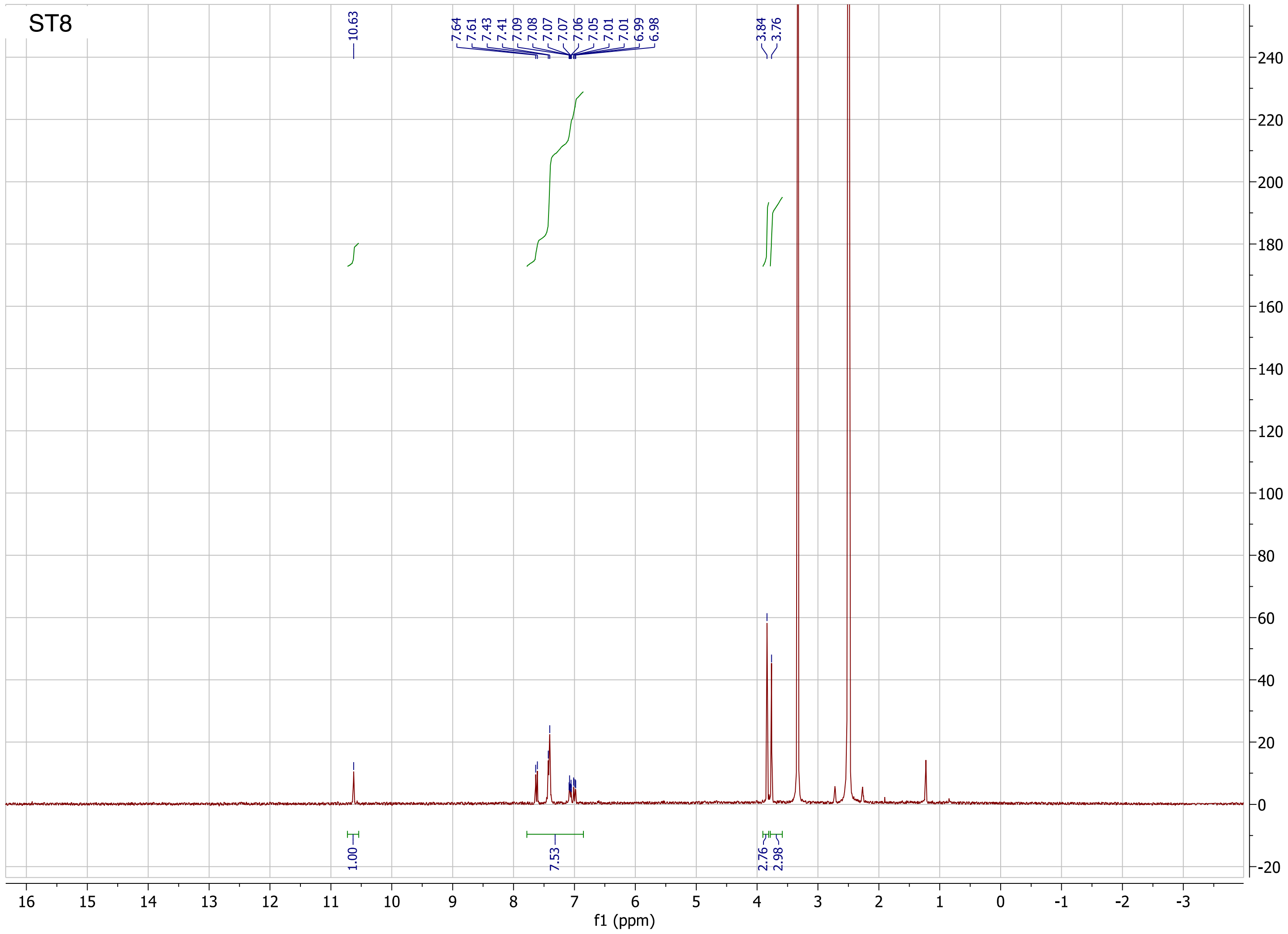

Figure S22. The ^{1}H NMR of compound ST8.

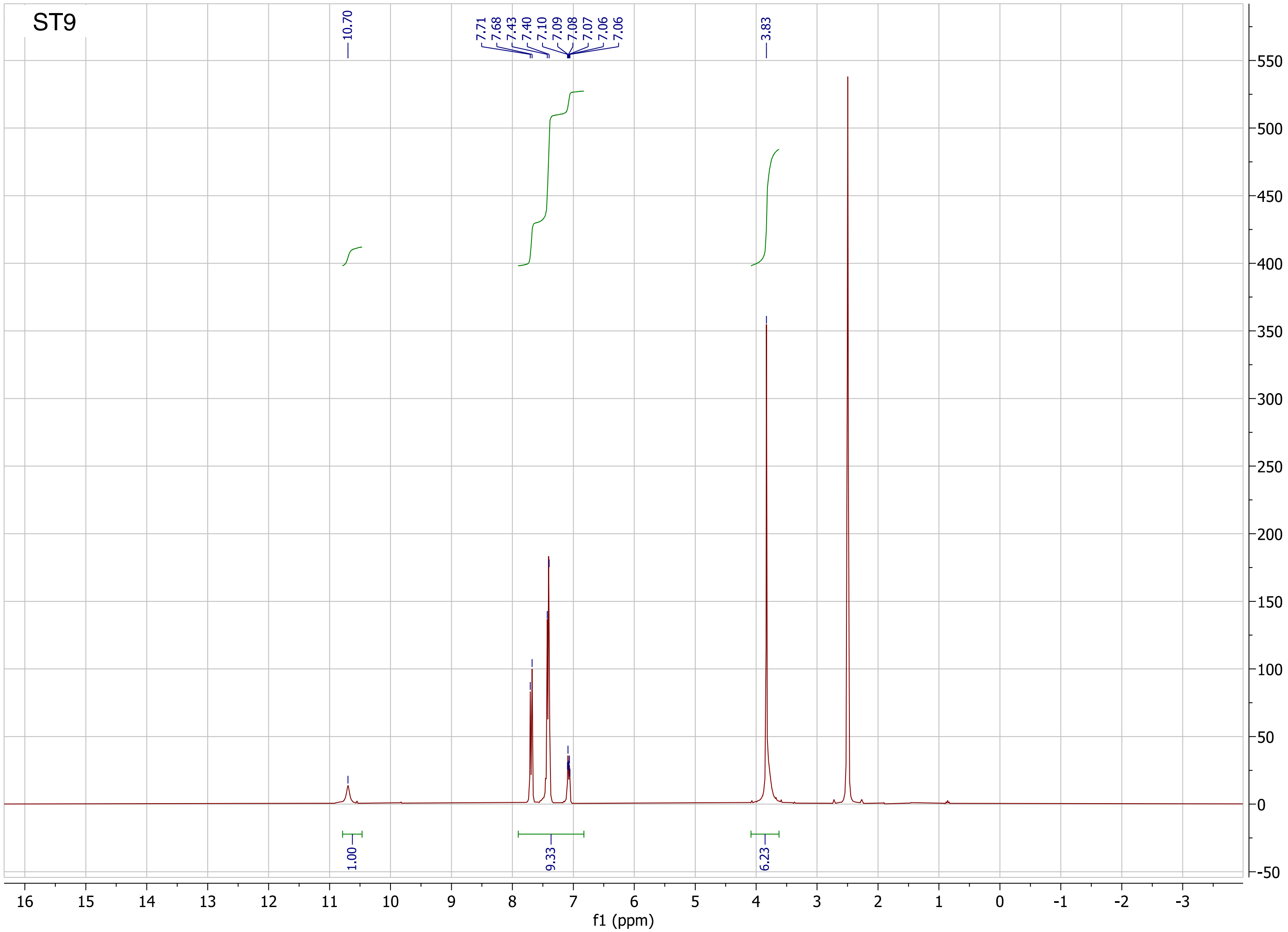

Figure S23. The ^{1}H NMR of compound ST9.

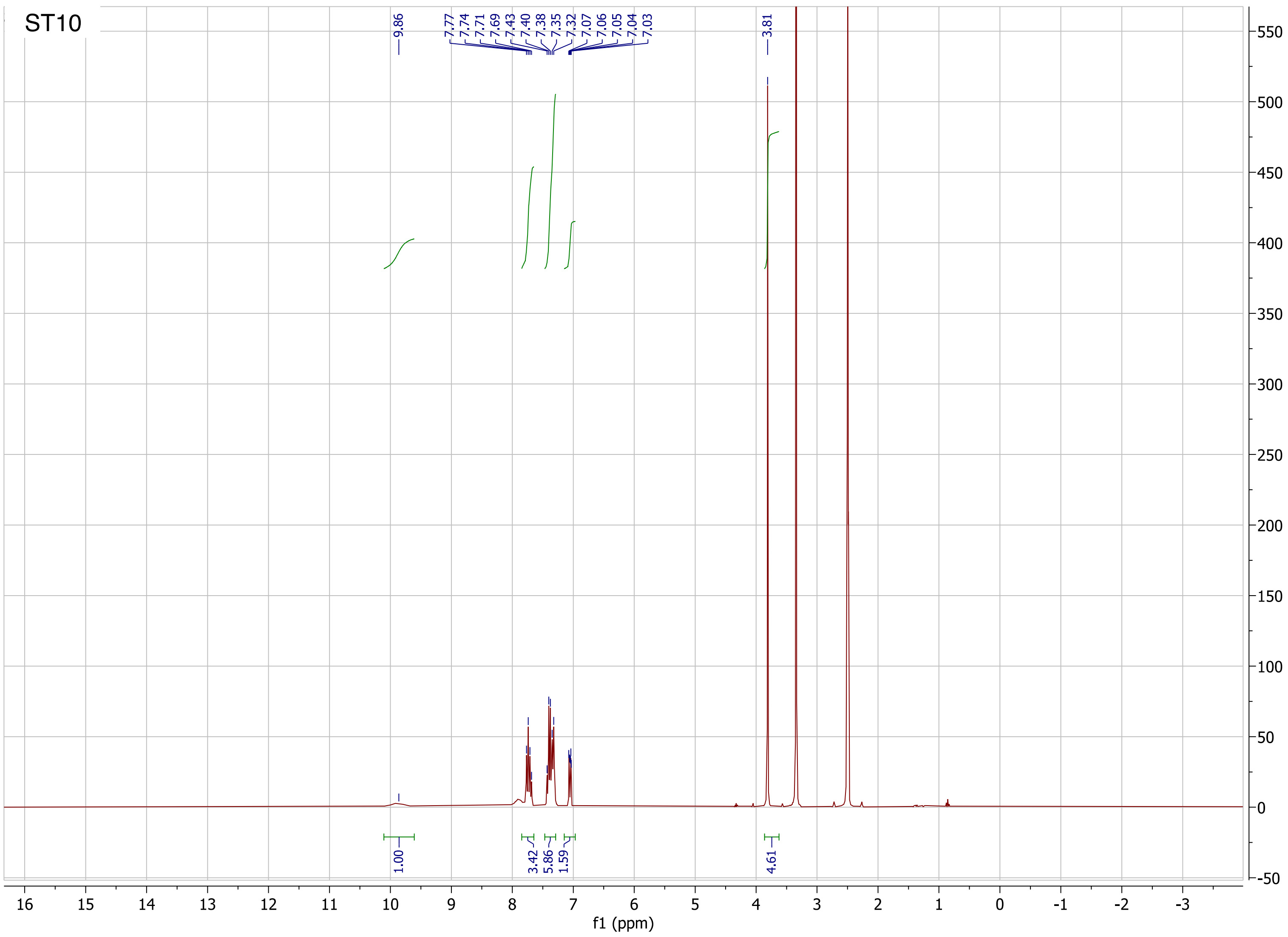

Figure S24. The ^{1}H NMR of compound ST10.

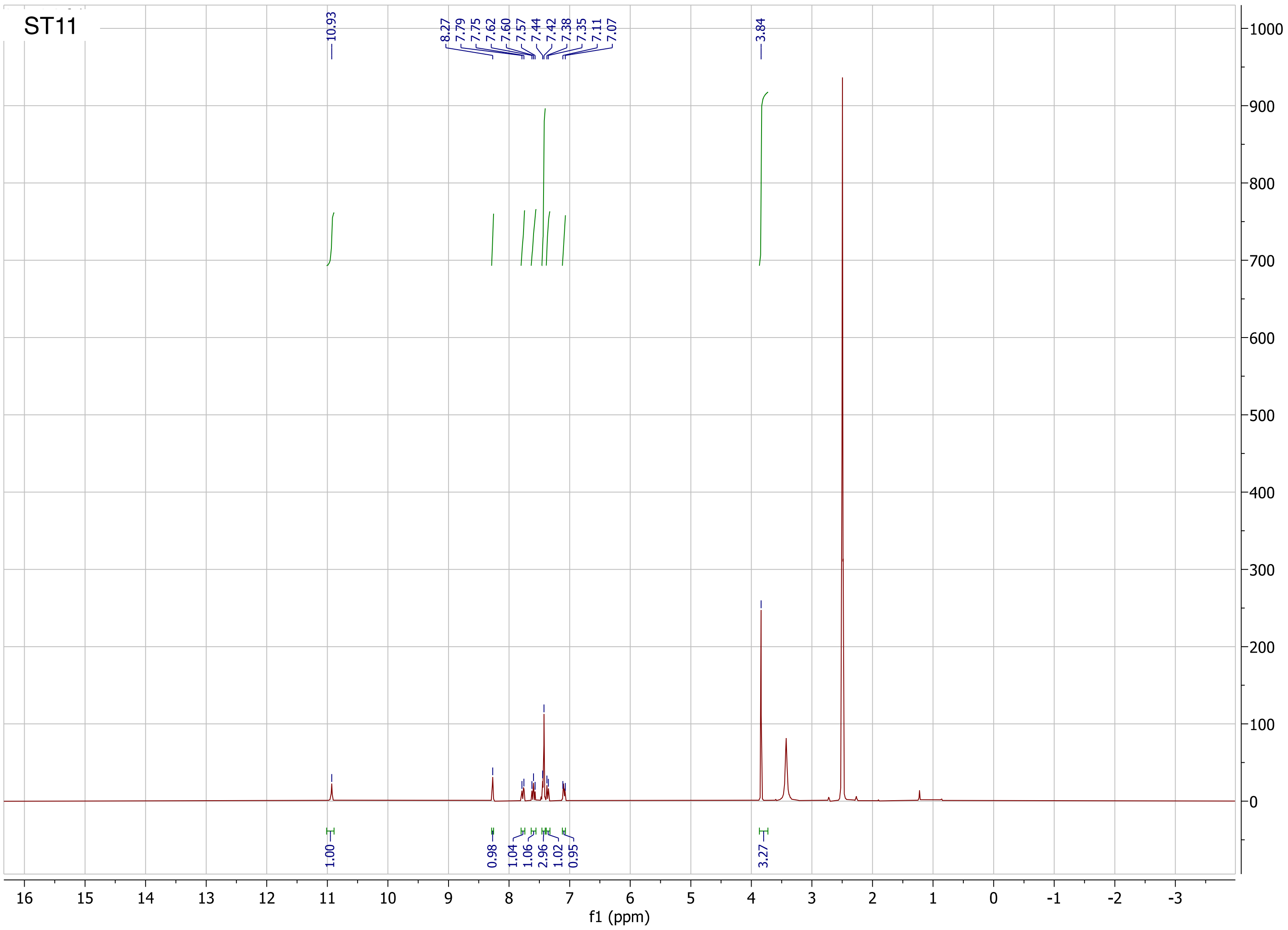

Figure S25. The ^{1}H NMR of compound ST11.

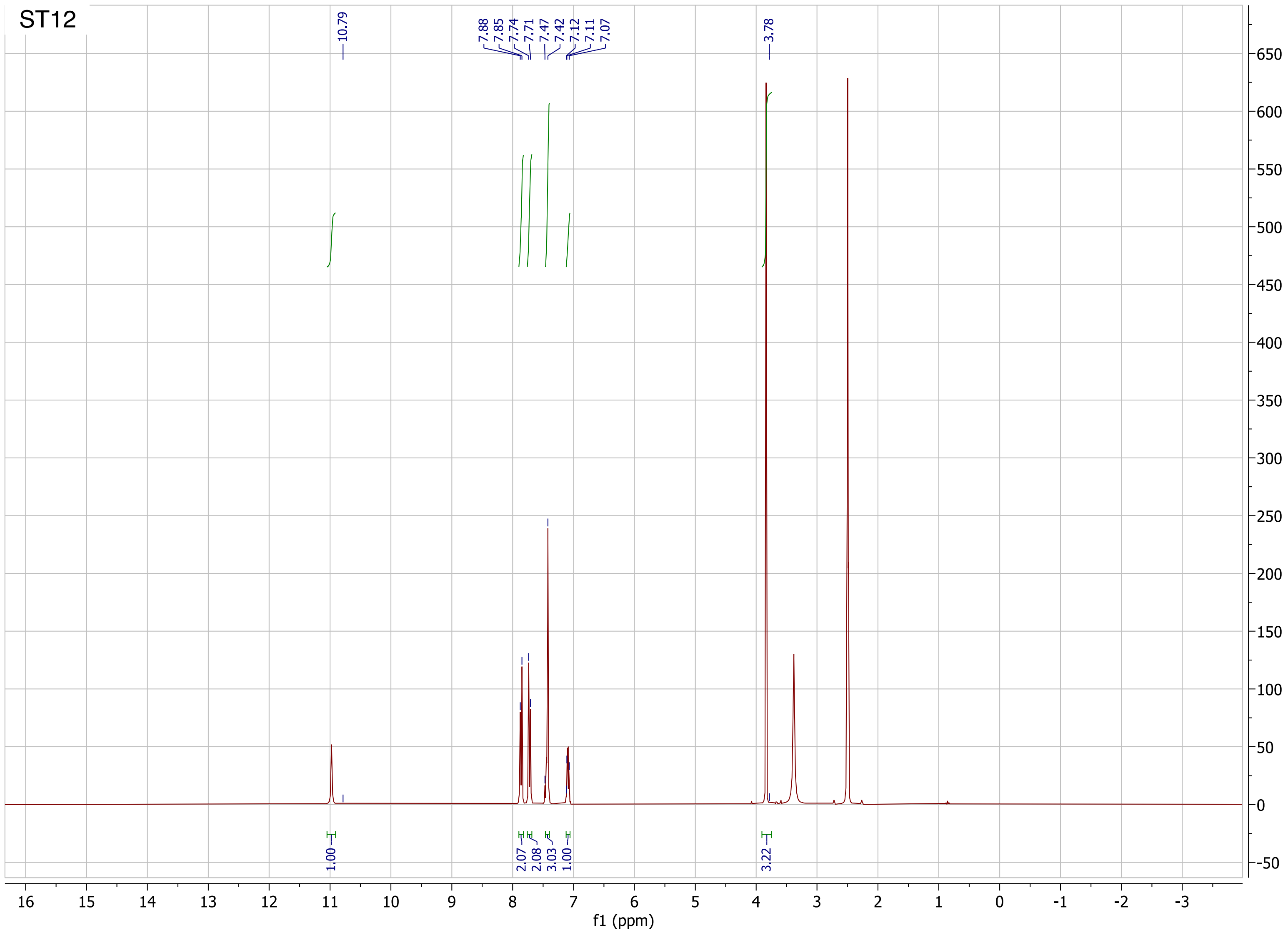

Figure S26. The ^{1}H NMR of compound ST12.

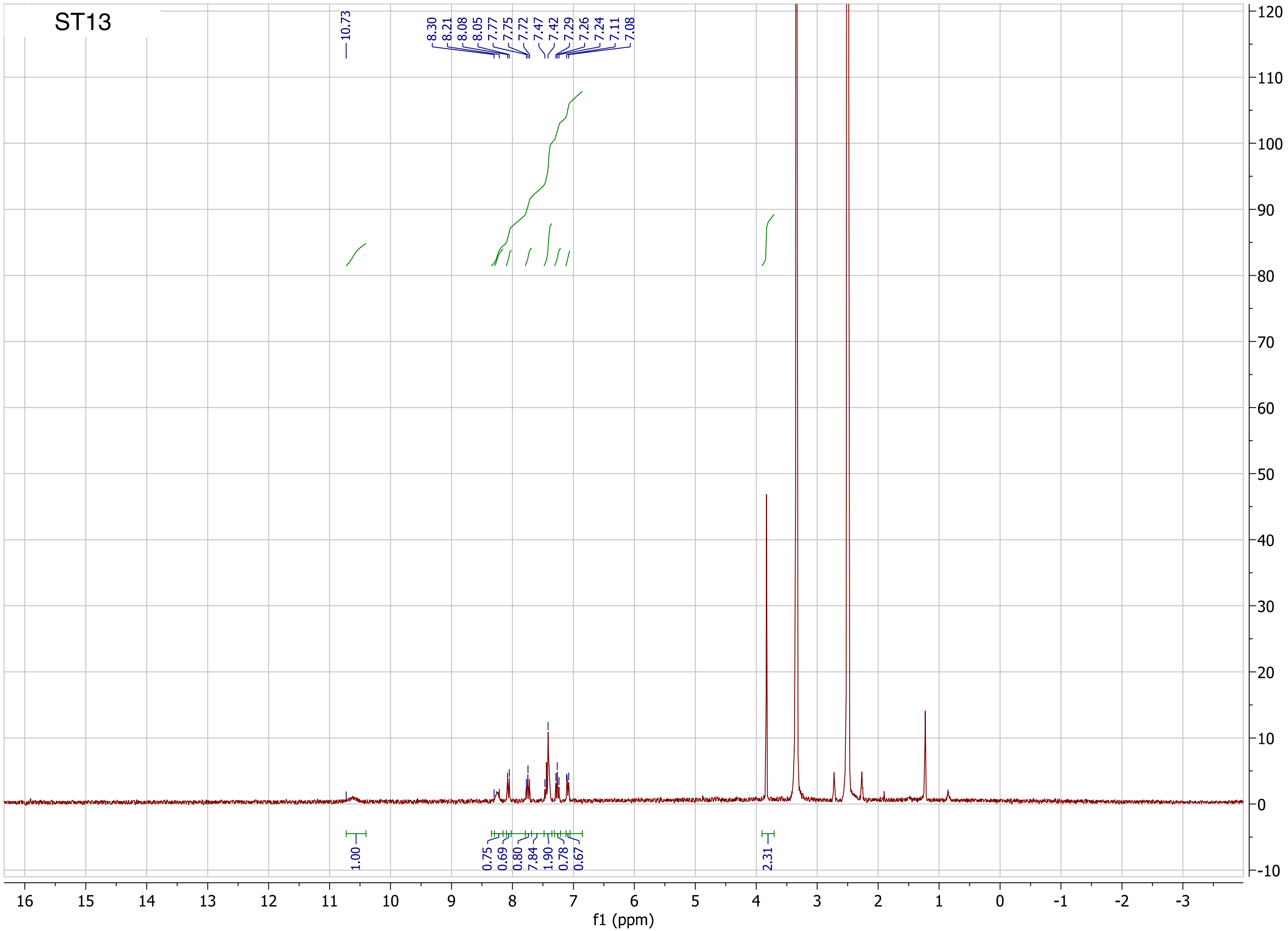

Figure S27. The ^{1}H NMR of compound ST13.

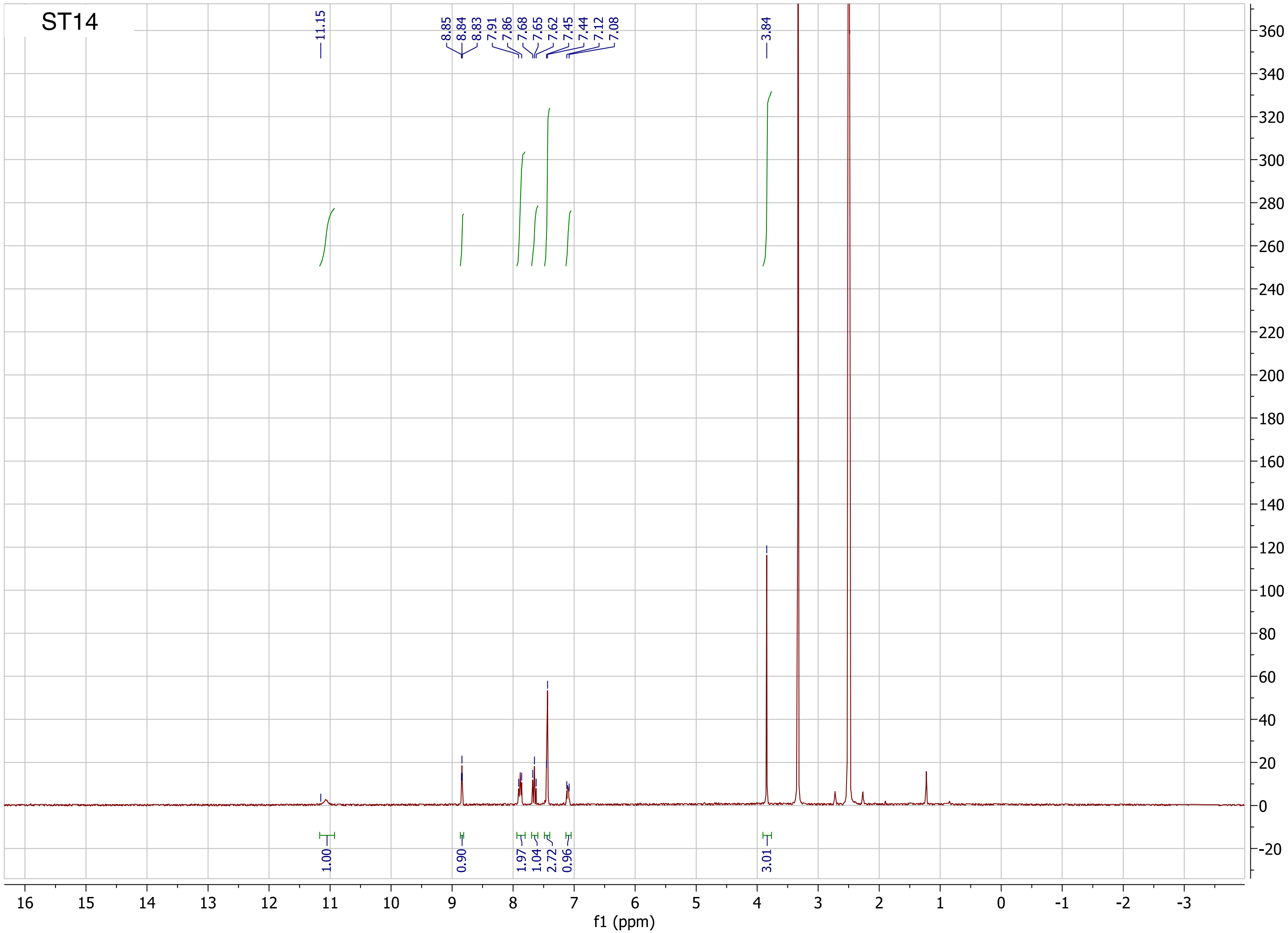

Figure S28. The ^{1}H NMR of compound ST14.

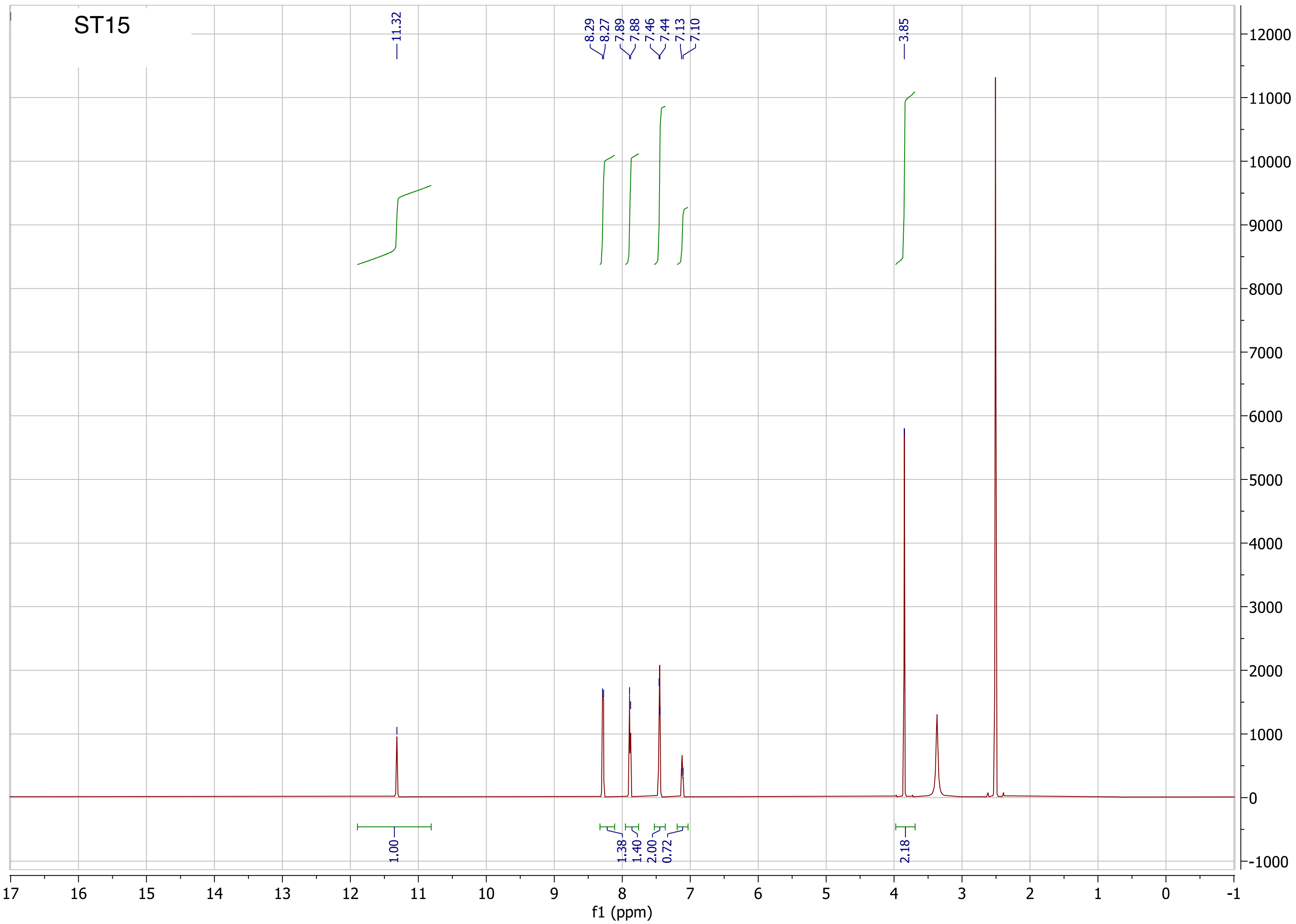

Figure S29. The ^{1}H NMR of compound ST15.

13. C NMR spectra of thiosemicarbazide derivatives:

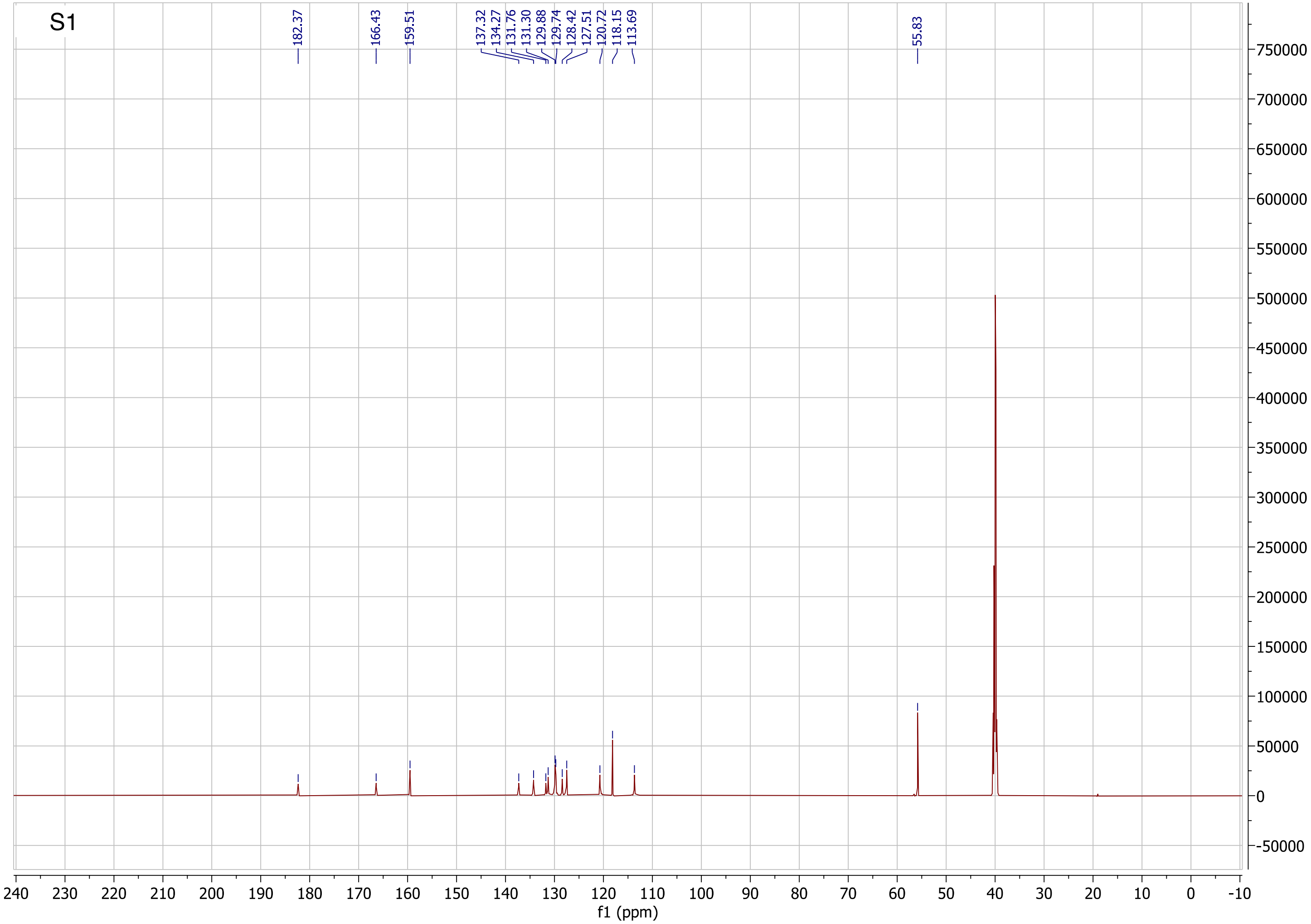

Figure S30. The ^{13}C NMR of compound S1.

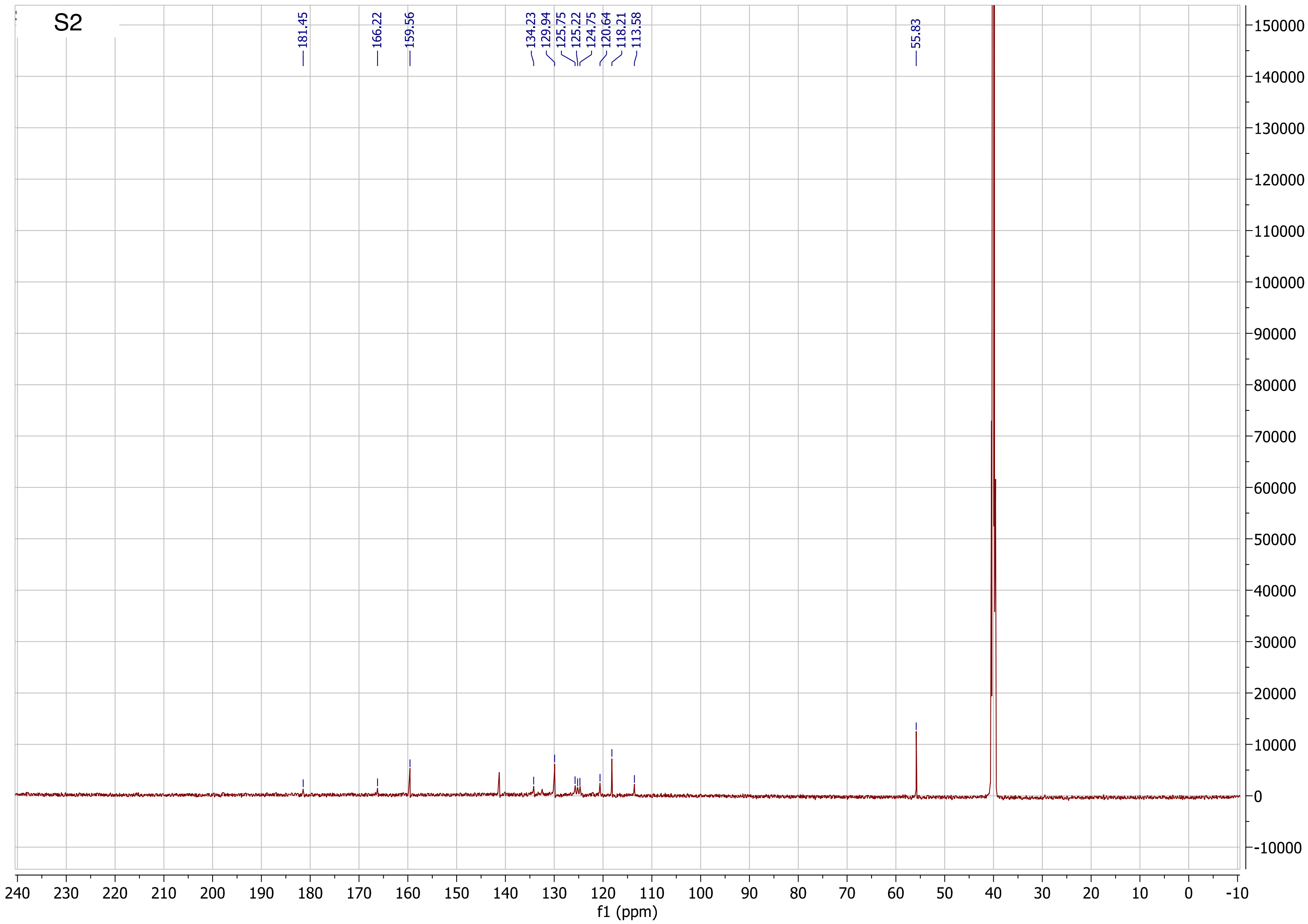

Figure S31. The ^{13}C NMR of compound S2.

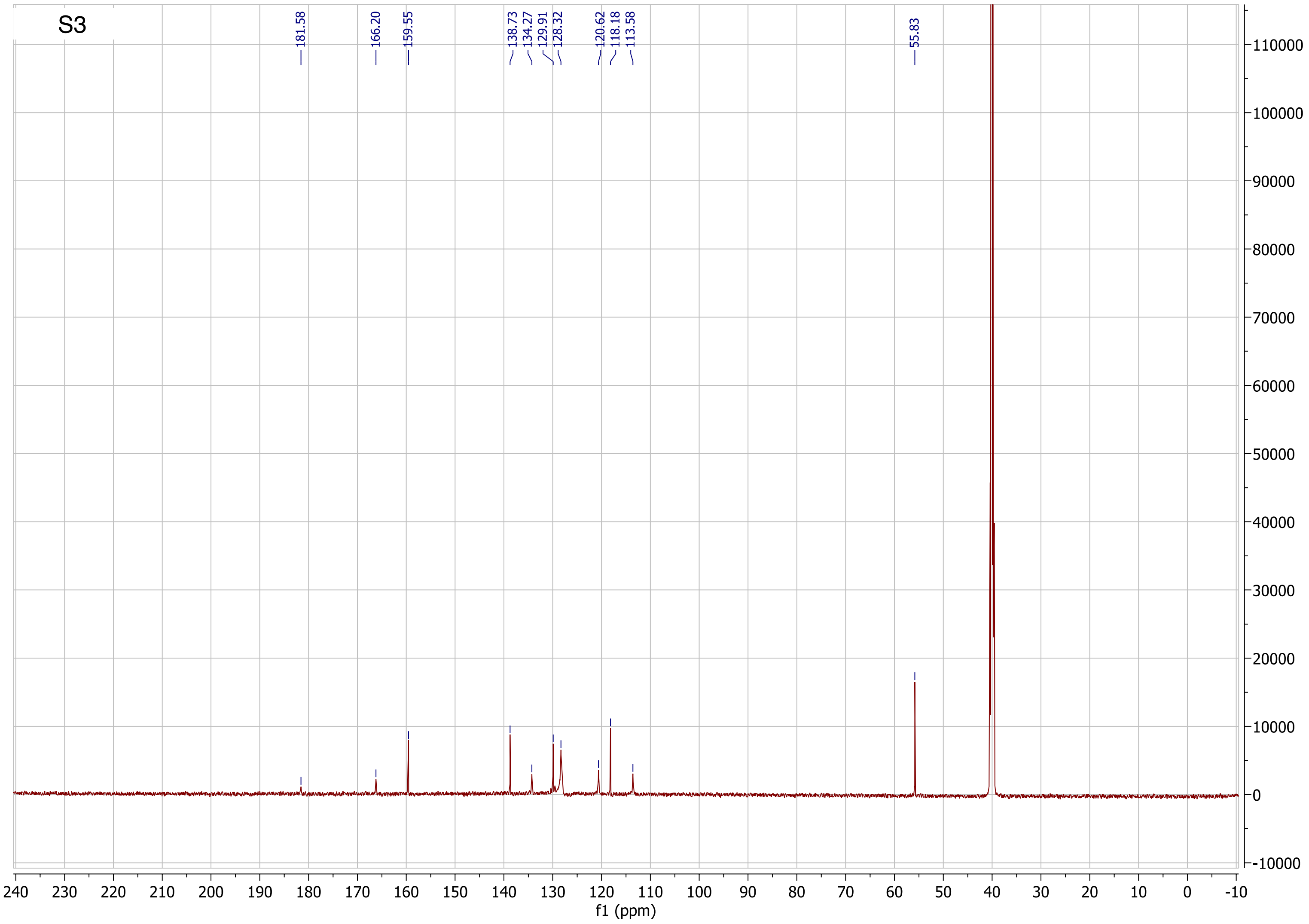

Figure S32. The ^{13}C NMR of compound S3.

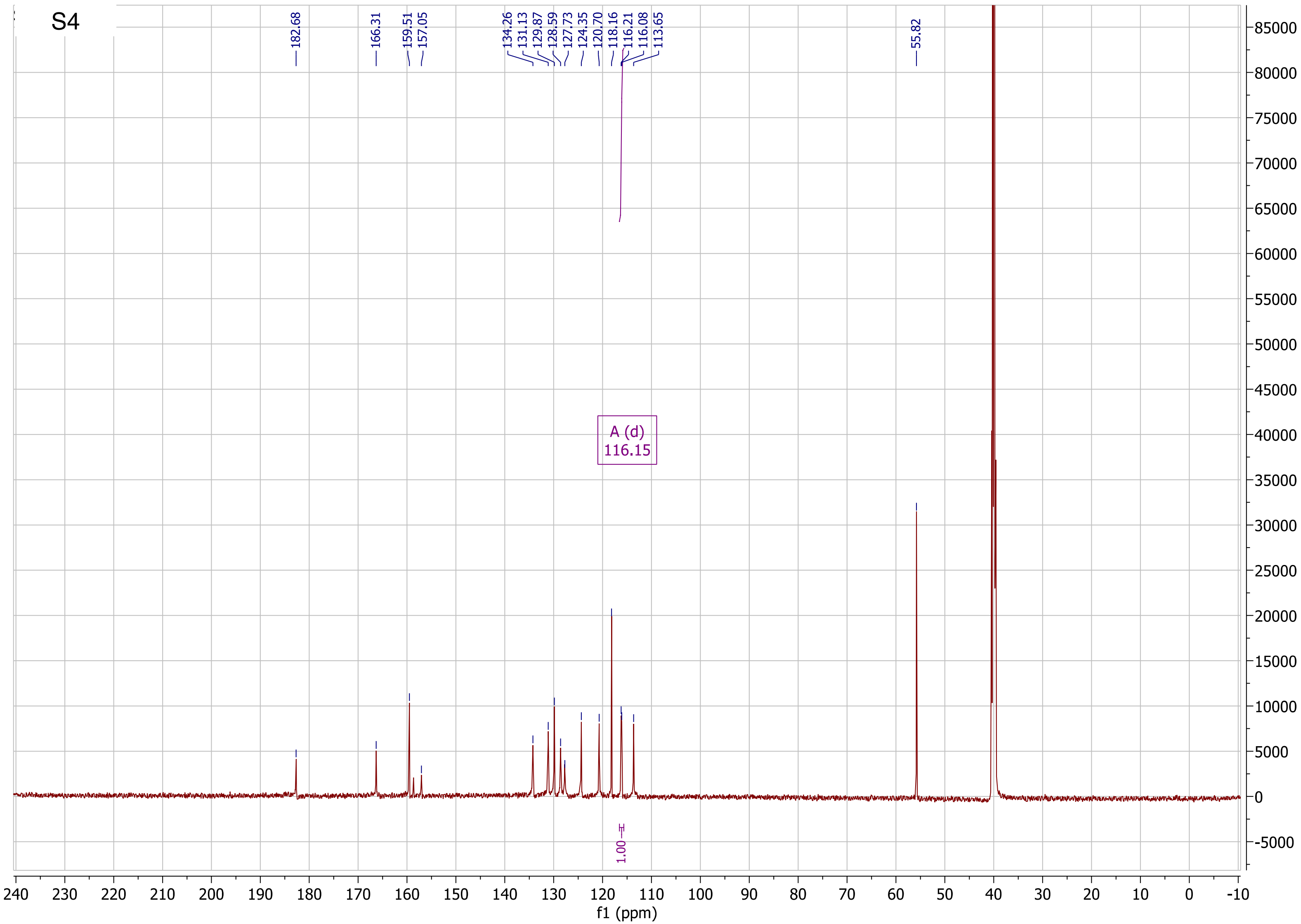

Figure S33. The ^{13}C NMR of compound S4.

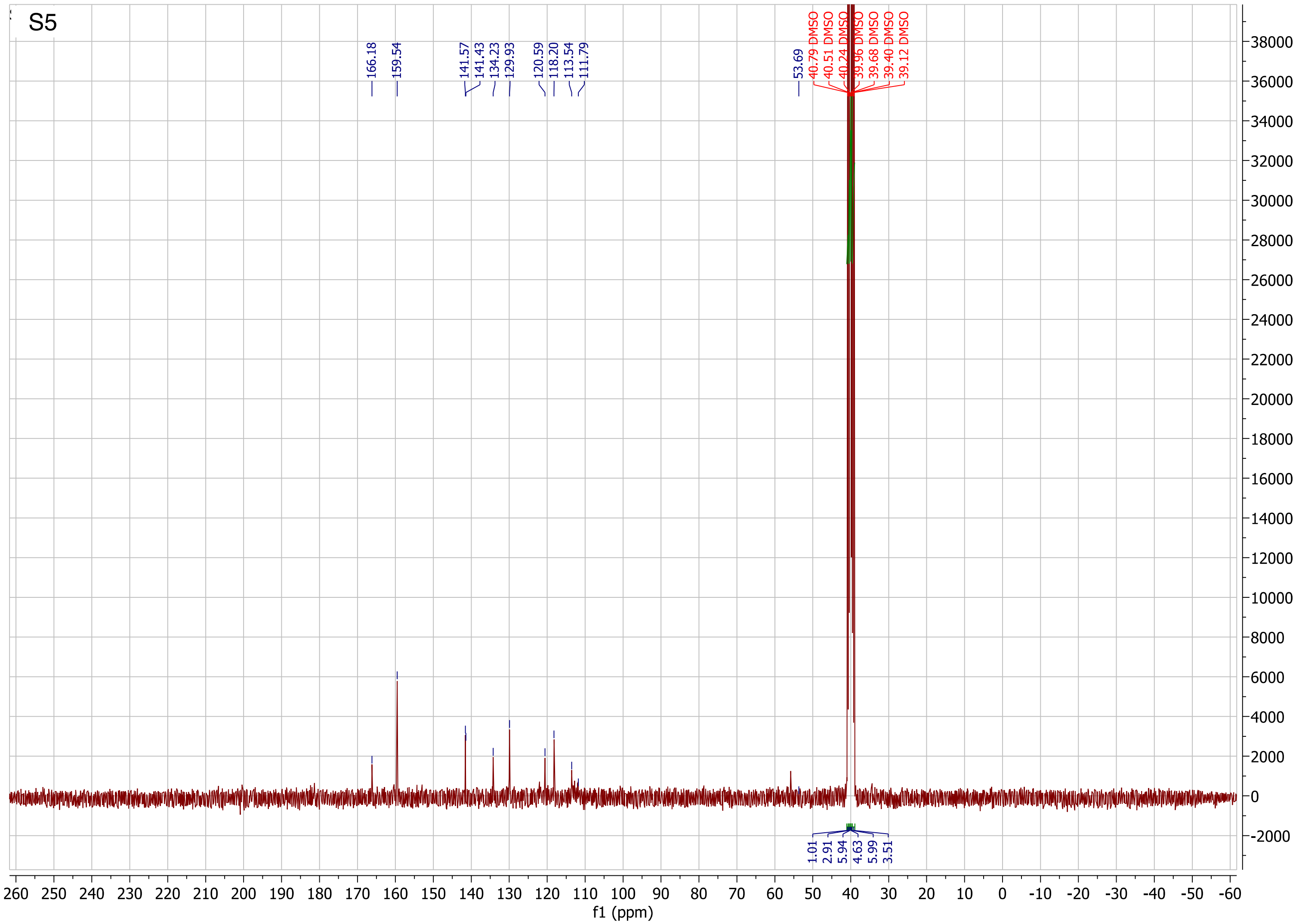

Figure S34. The ^{13}C NMR of compound S5.

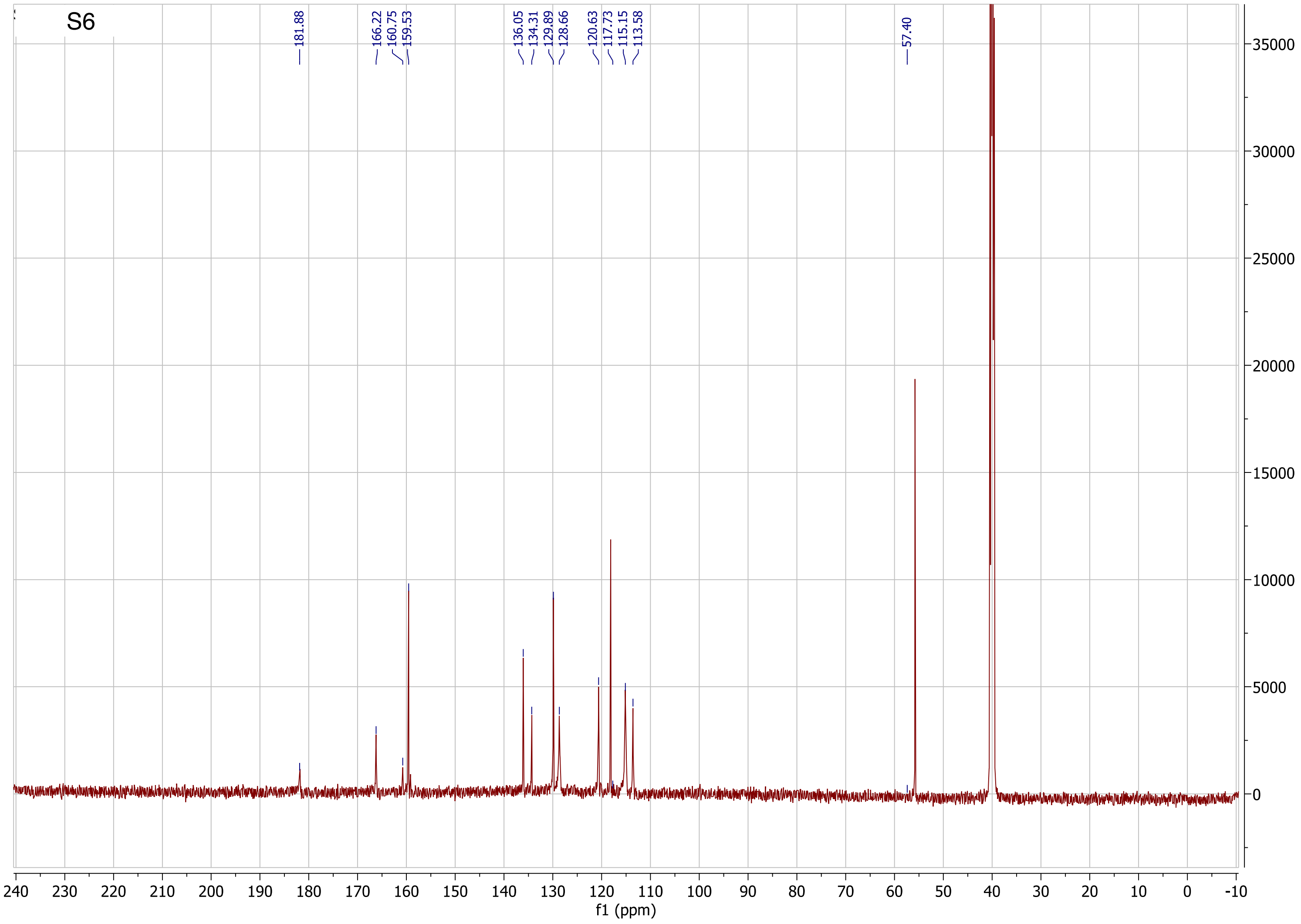

Figure S35. The ^{13}C NMR of compound S6.

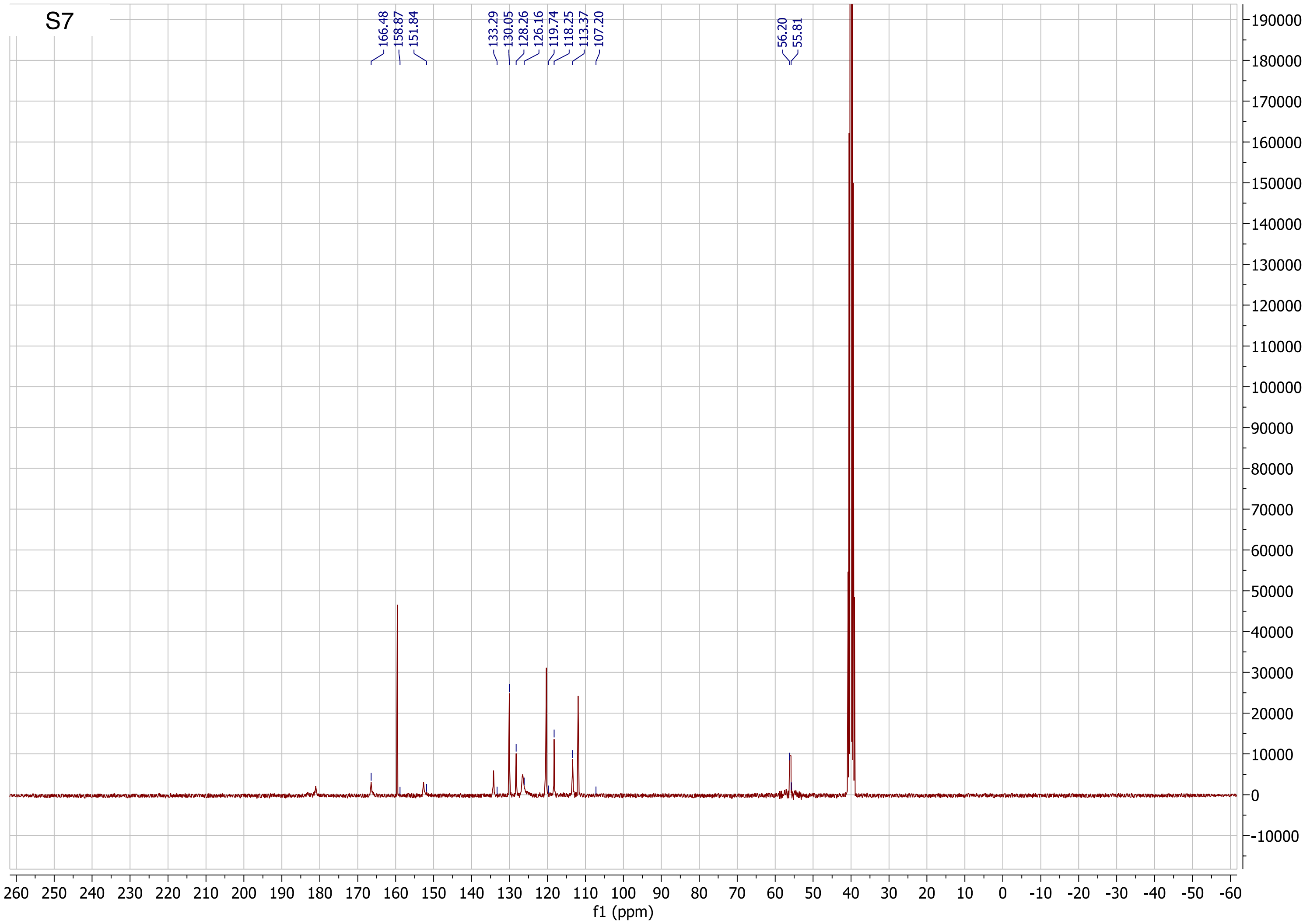

Figure S36. The ^{13}C NMR of compound S7.

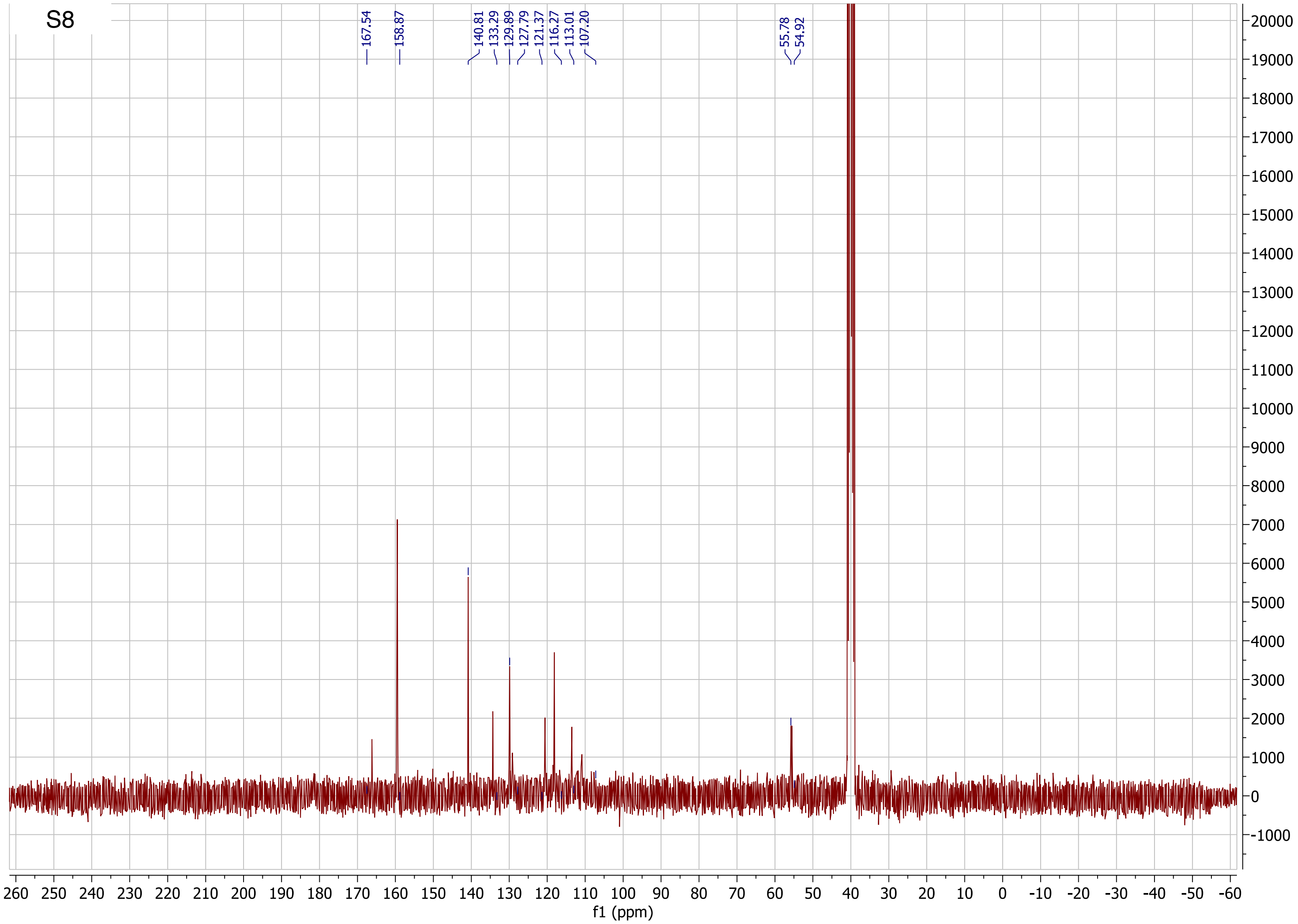

Figure S37. The ^{13}C NMR of compound S8.

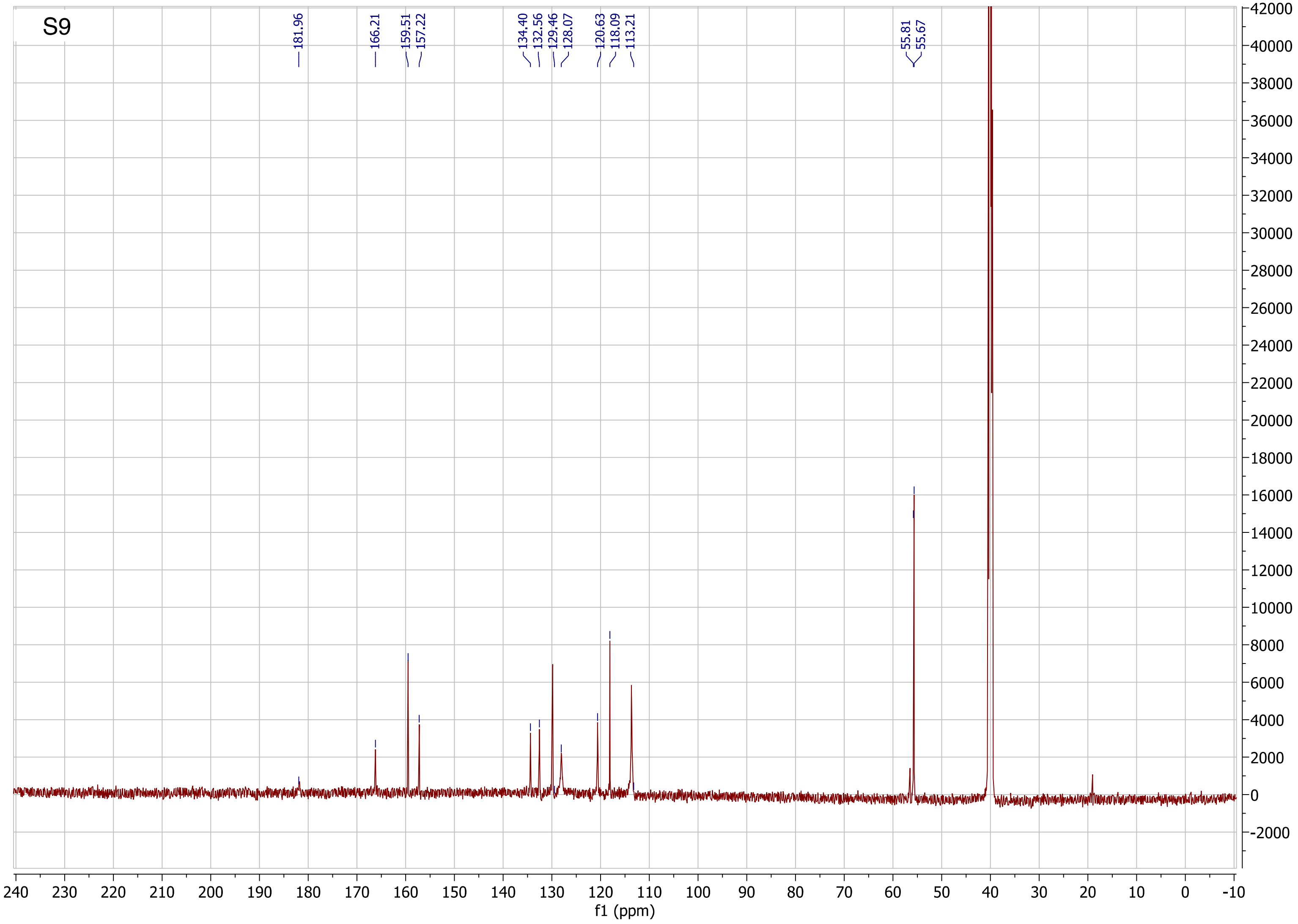

Figure S38. The ^{13}C NMR of compound S9.

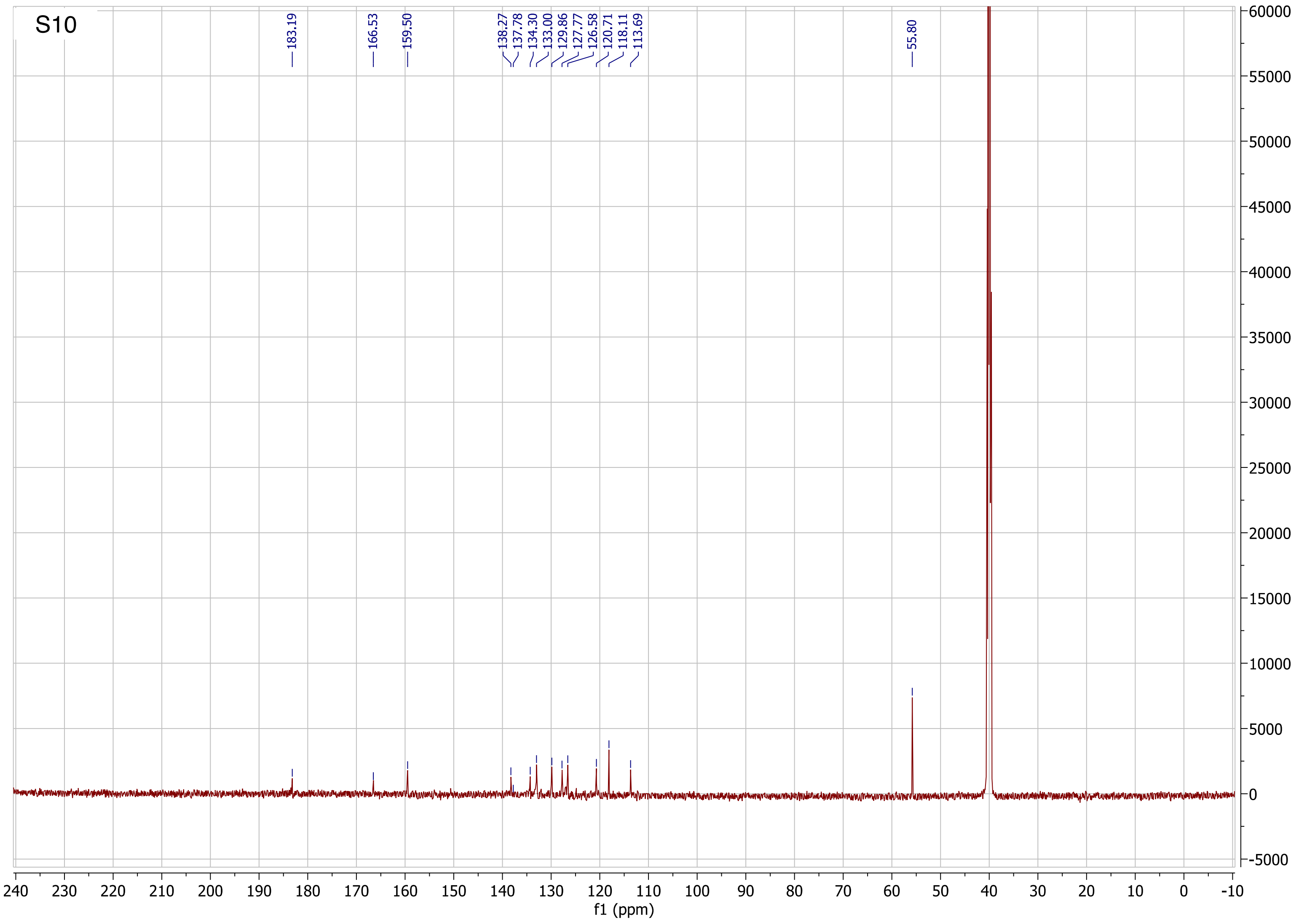

Figure S39. The ^{13}C NMR of compound S10.

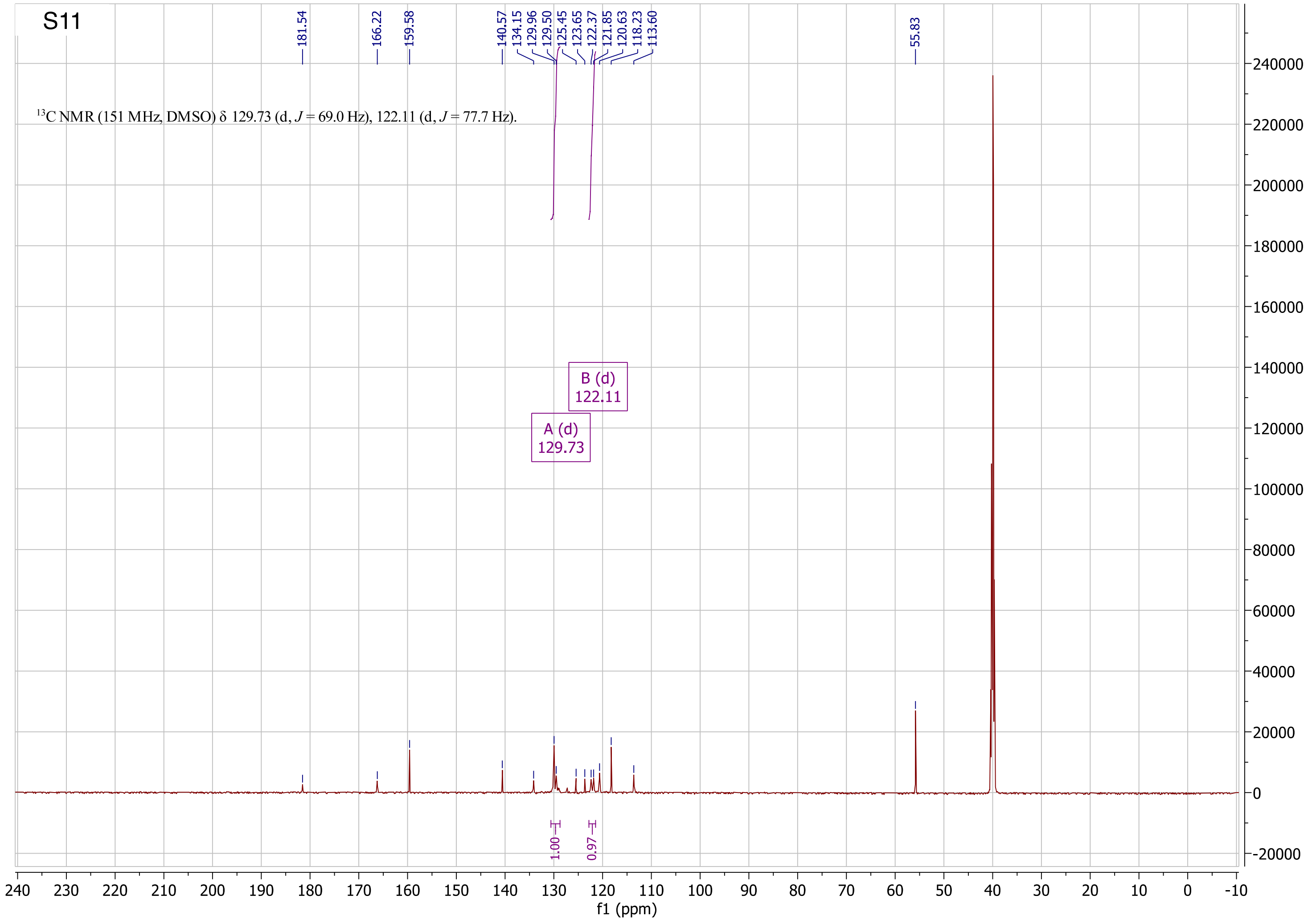

Figure S40. The ^{13}C NMR of compound S11.

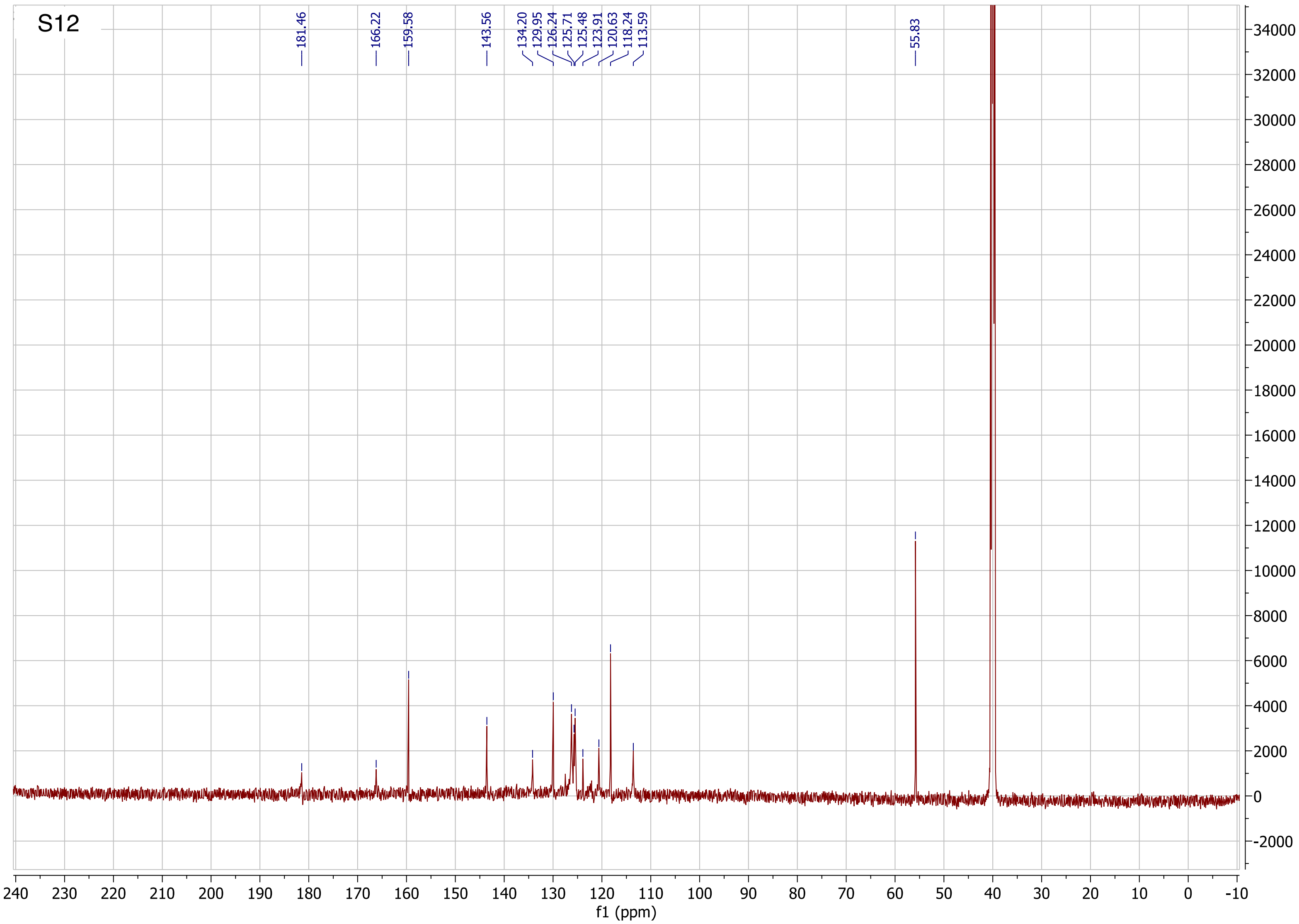

Figure S41. The ^{13}C NMR of compound S12.

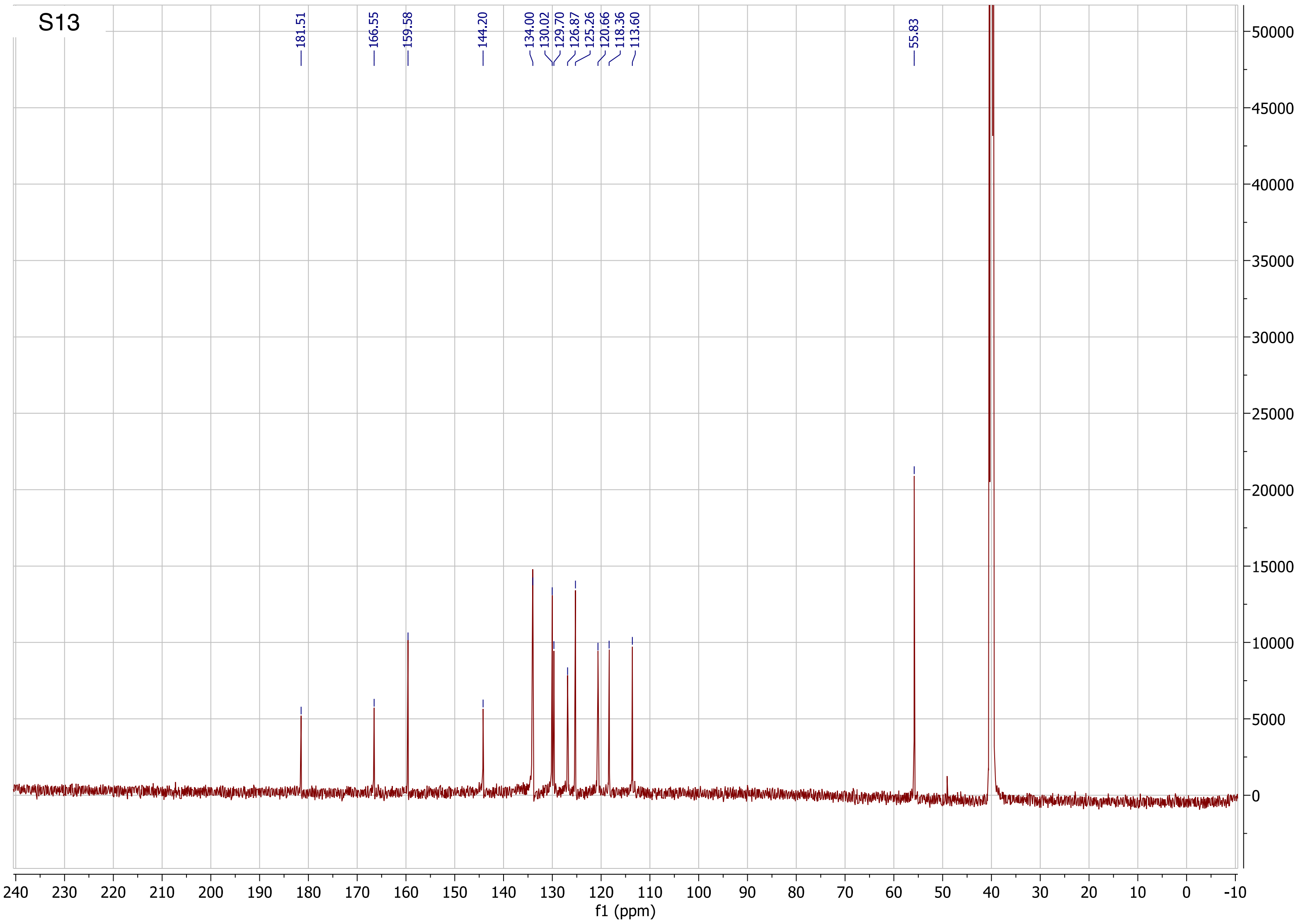

Figure S42. The ^{13}C NMR of compound S13.

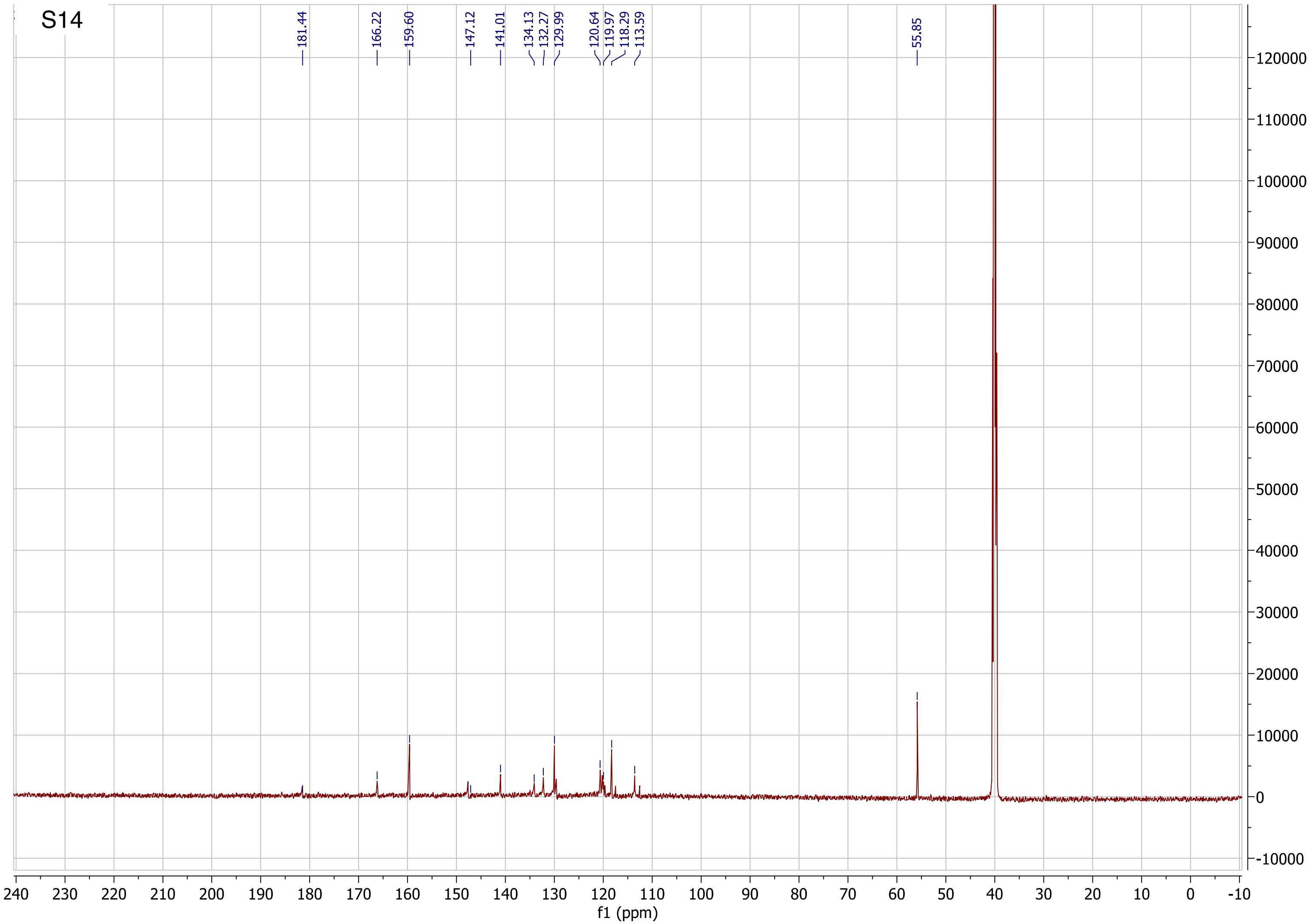

Figure S43. The ^{13}C NMR of compound S14.

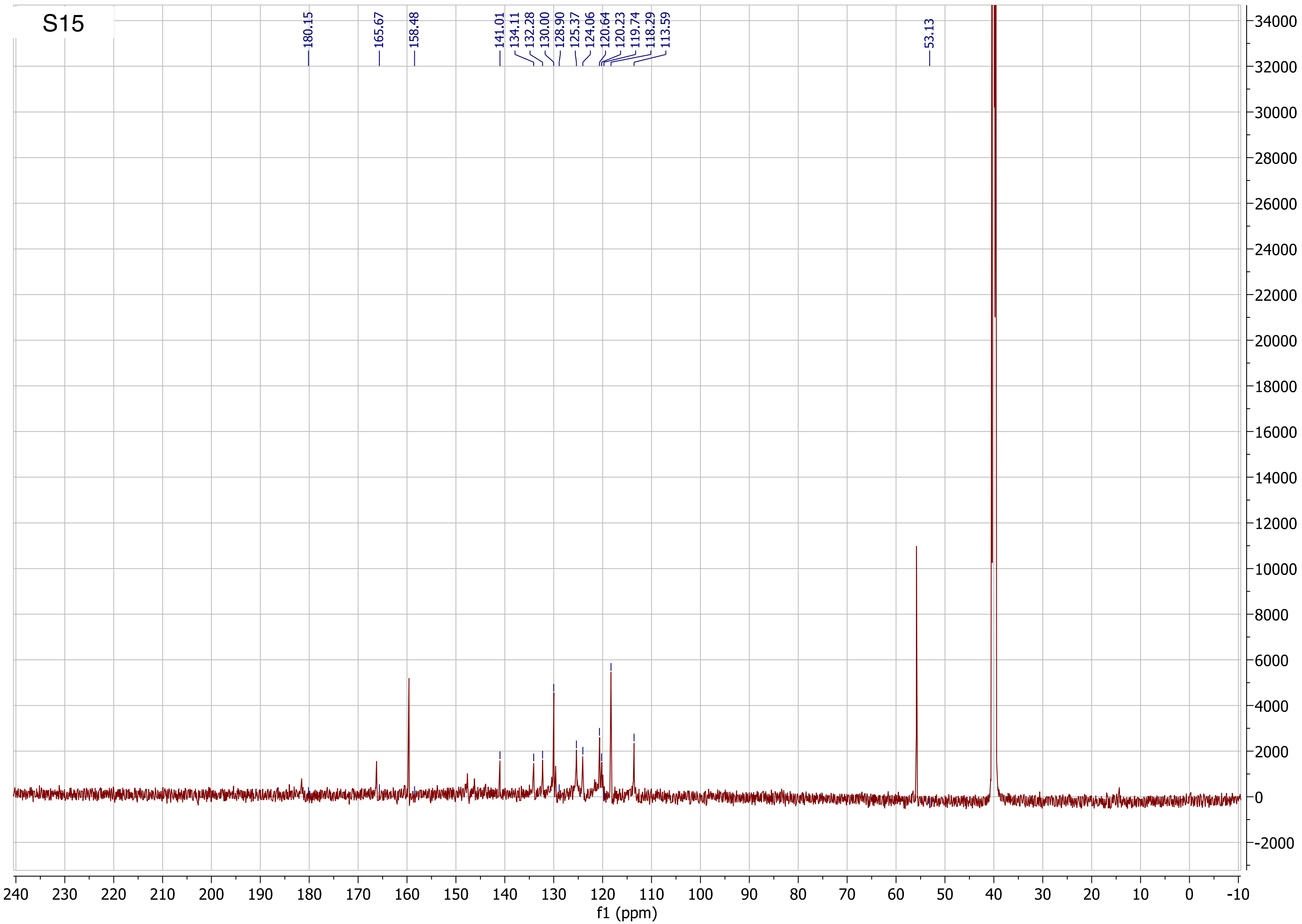

Figure S44. The ^{13}C NMR of compound S15.

[13.] C NMR spectra of 1,3,4-thiadiazole derivatives:

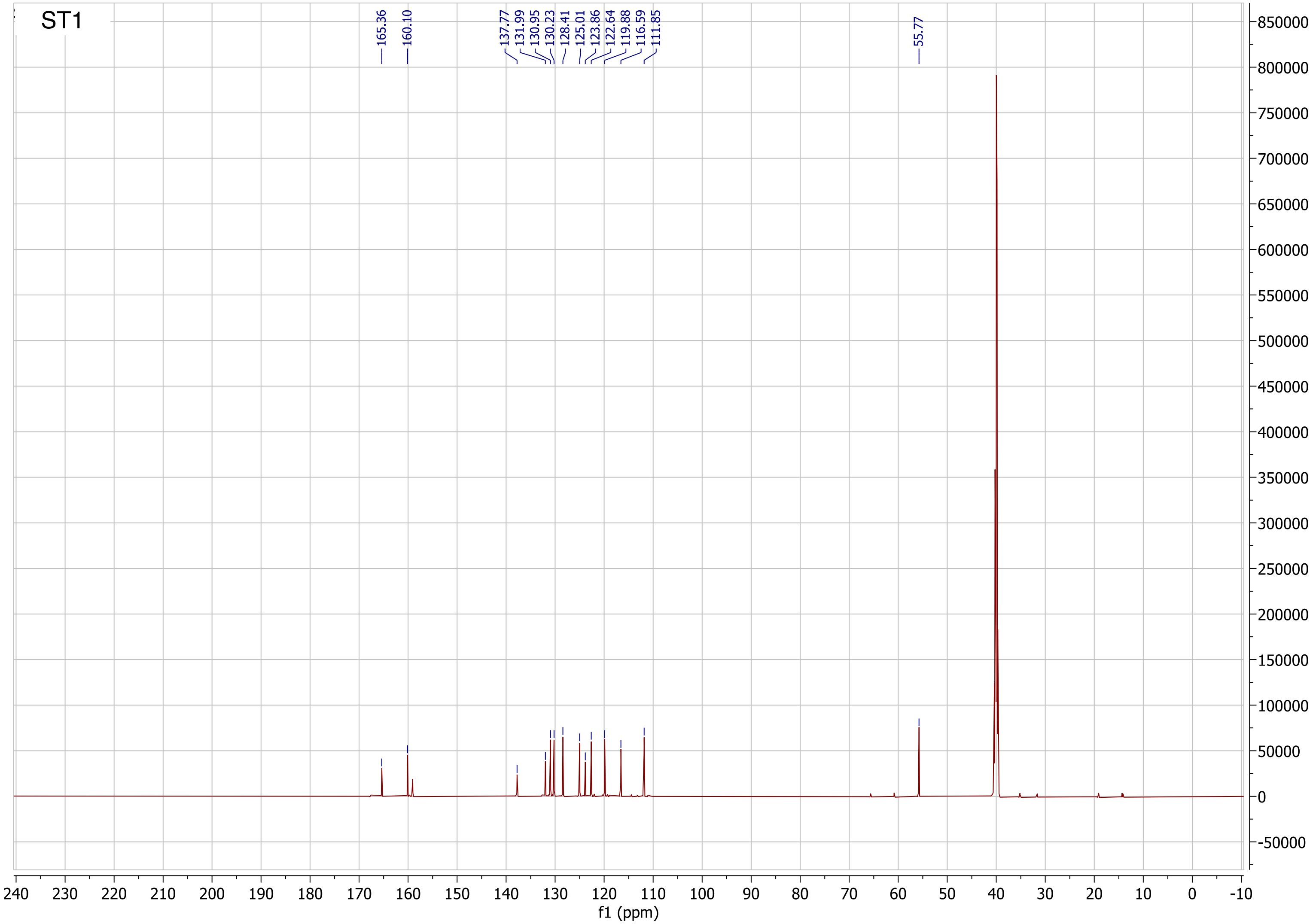

Figure S45. The ^{13}C NMR of compound ST1.

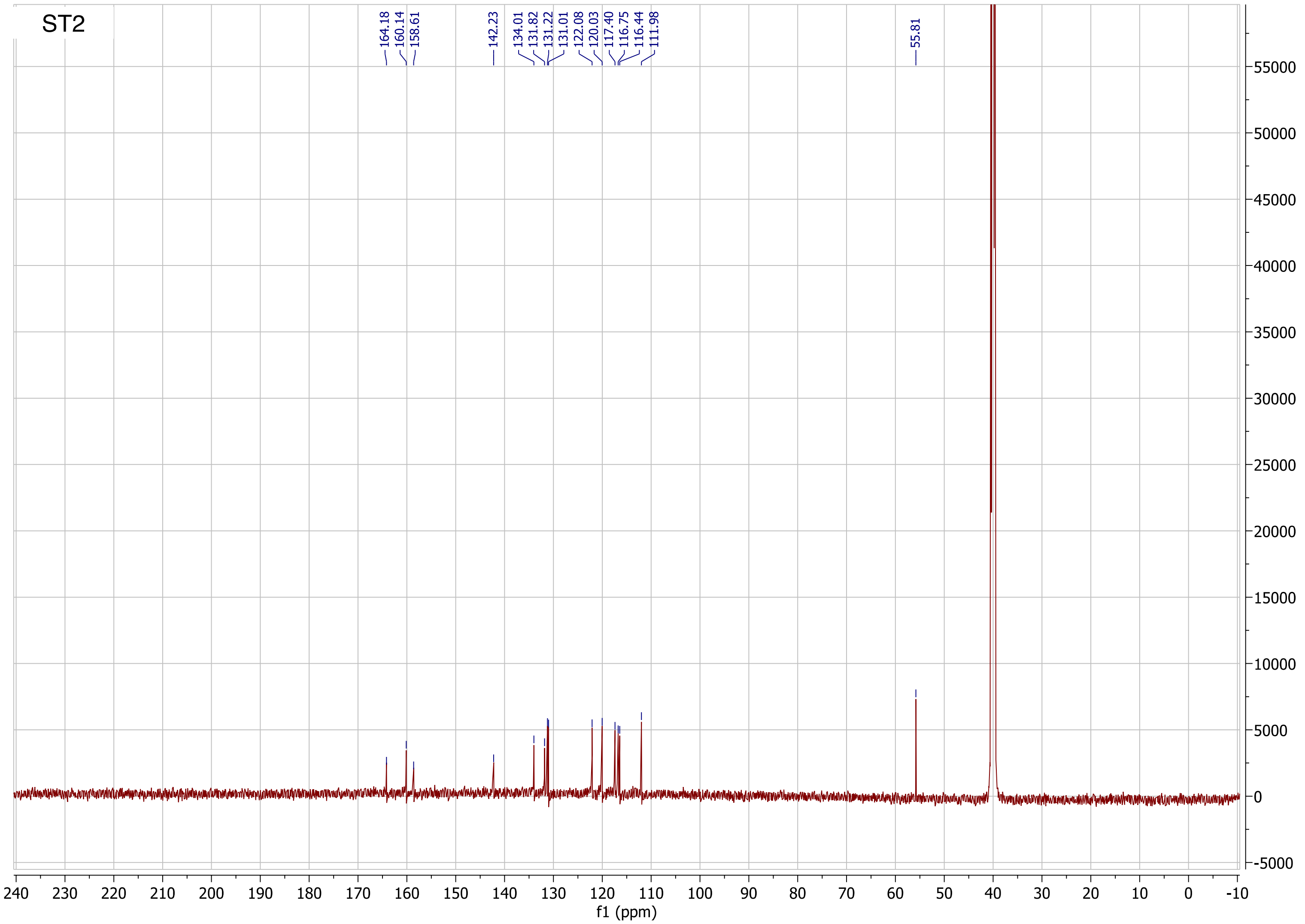

Figure S46. The ^{13}C NMR of compound ST2.

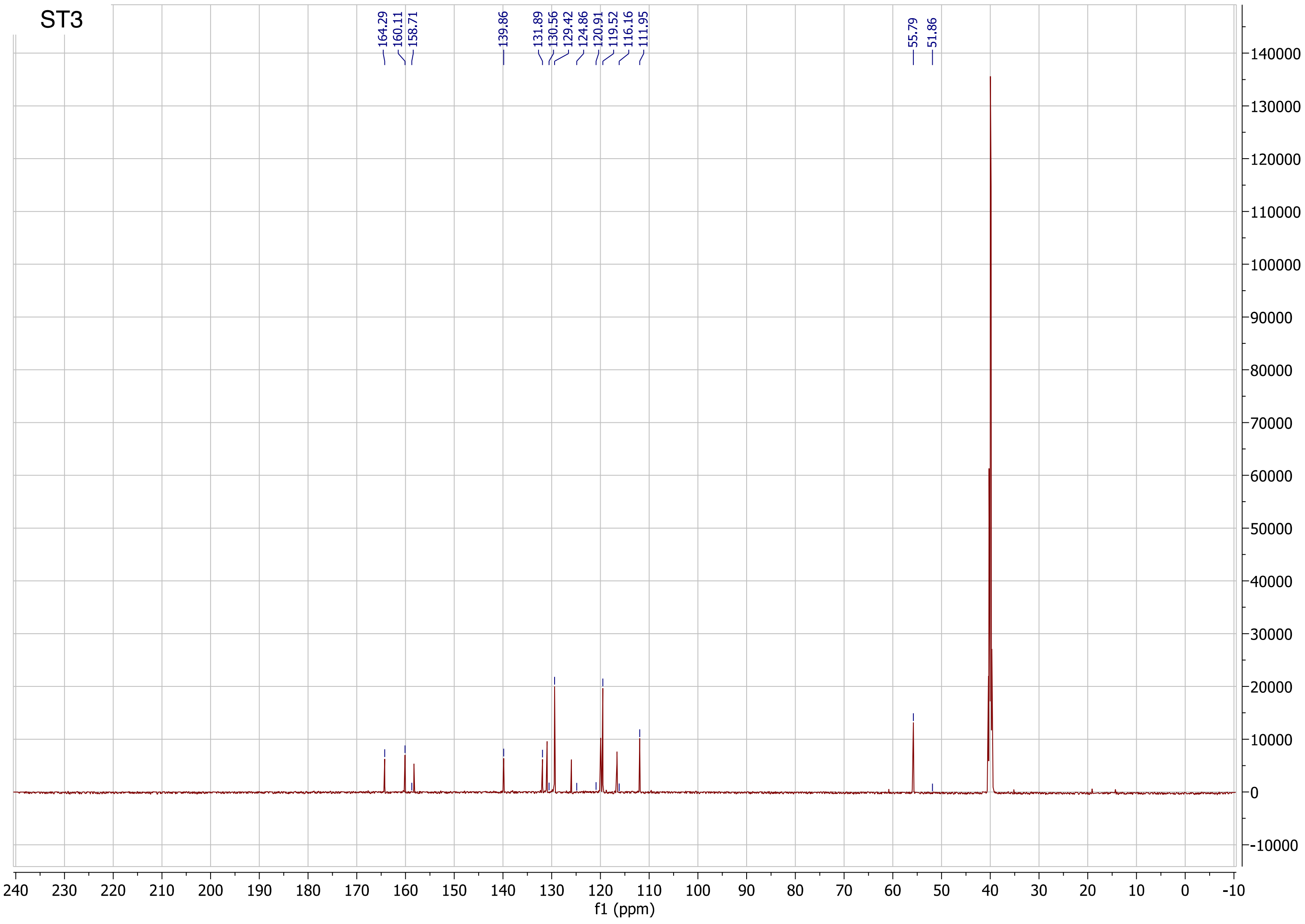

Figure S47. The ^{13}C NMR of compound ST3.

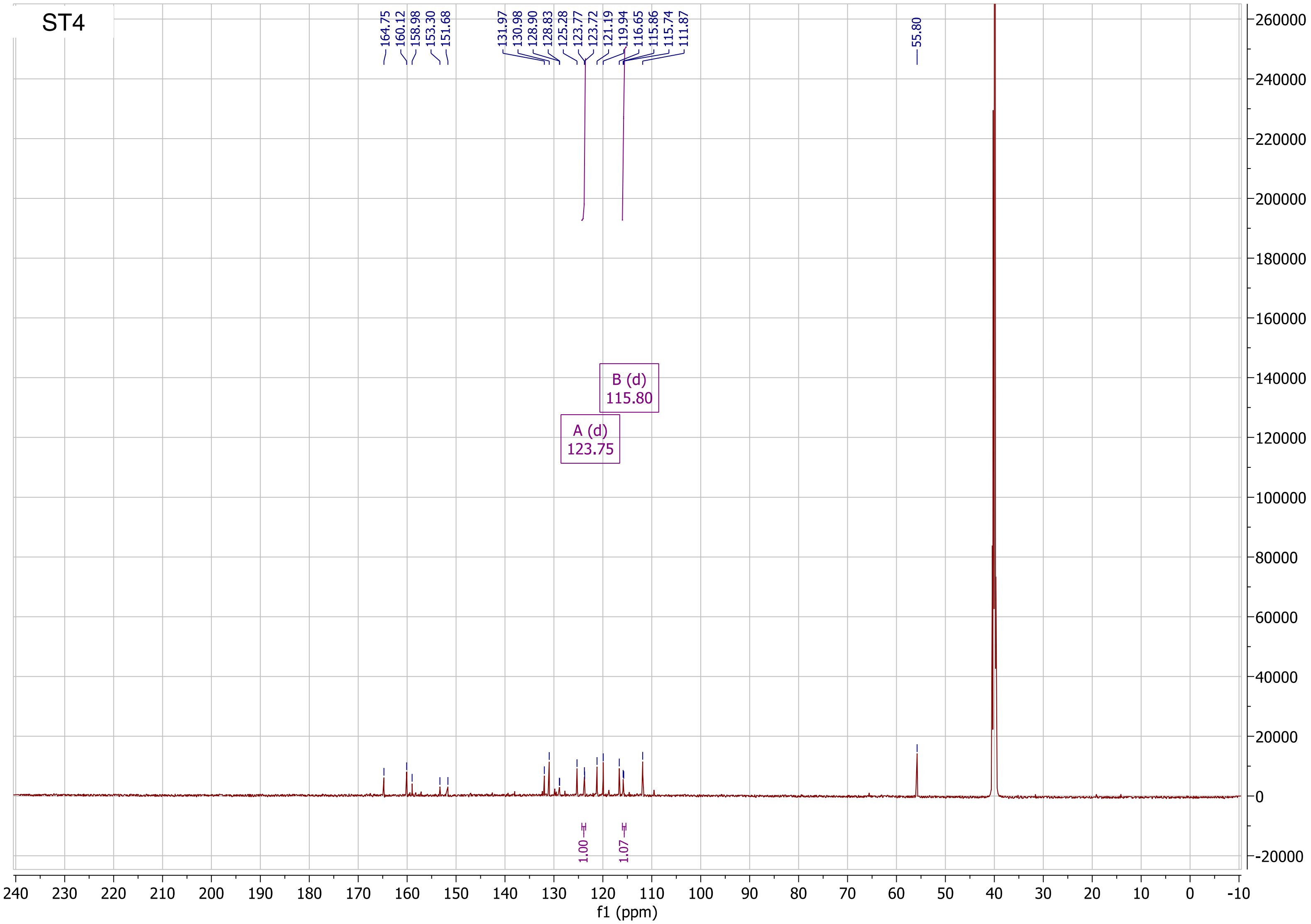

Figure S48. The ^{13}C NMR of compound ST4.

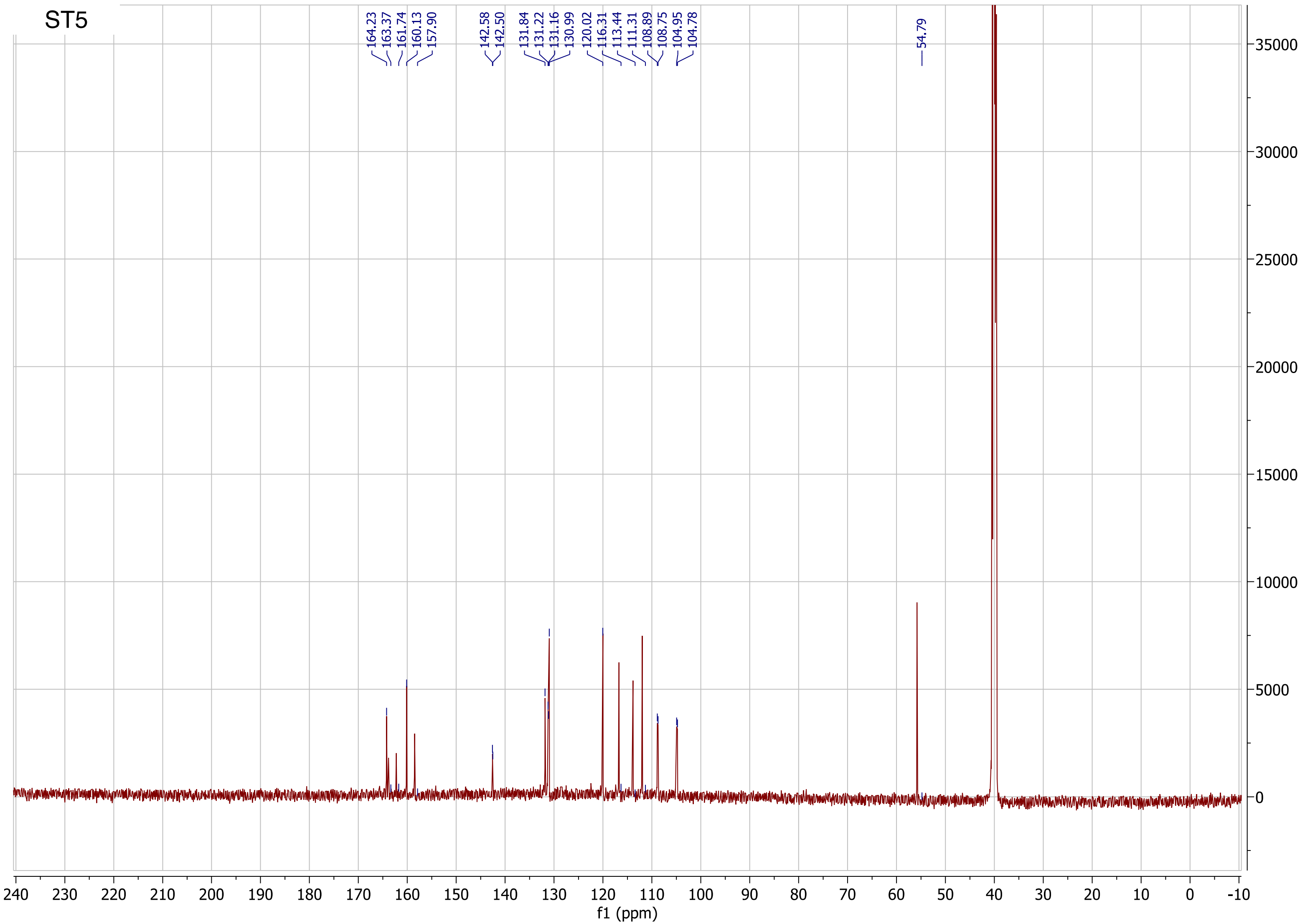

Figure S49. The ^{13}C NMR of compound ST5.

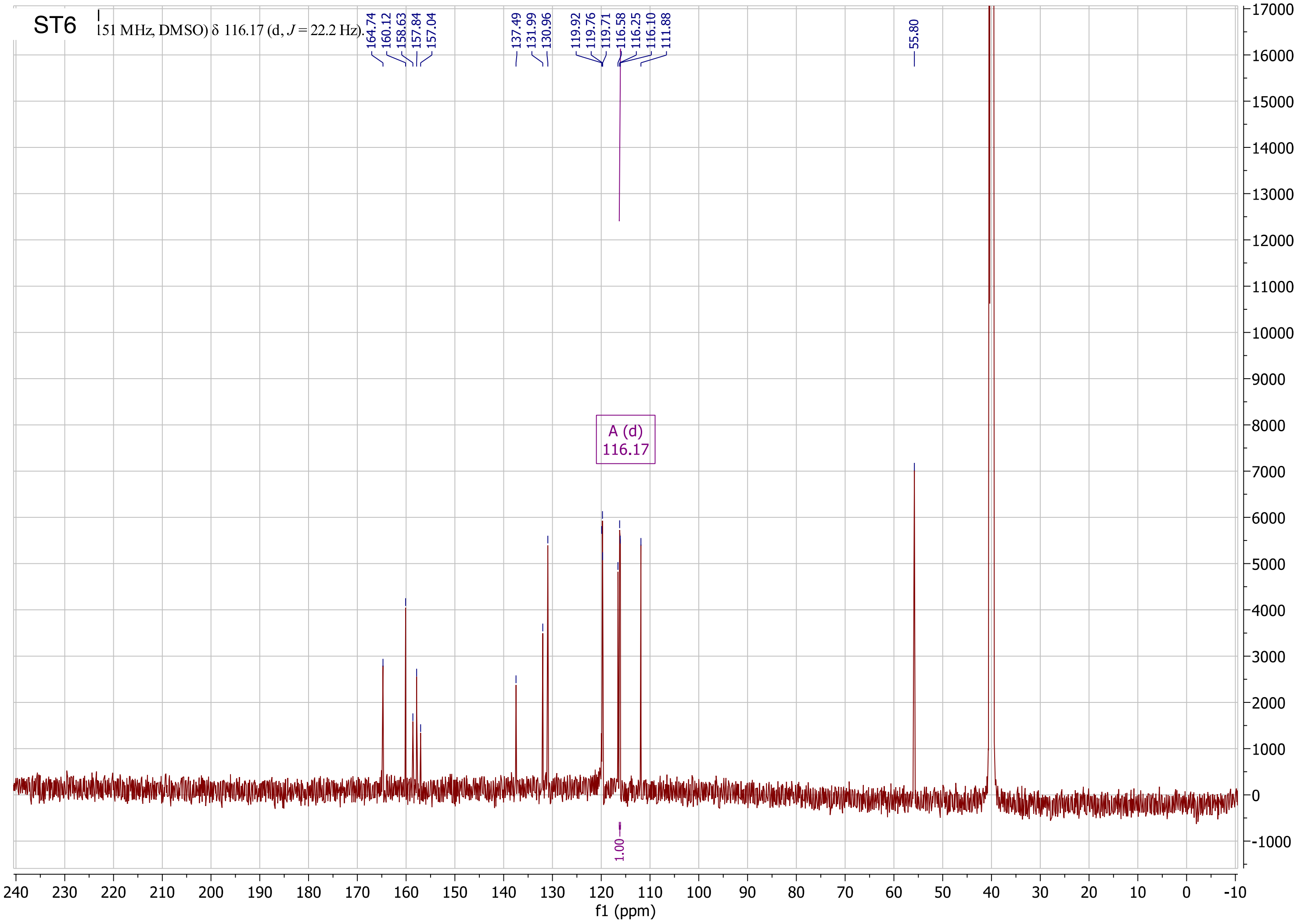

Figure S50. The ^{13}C NMR of compound ST6.

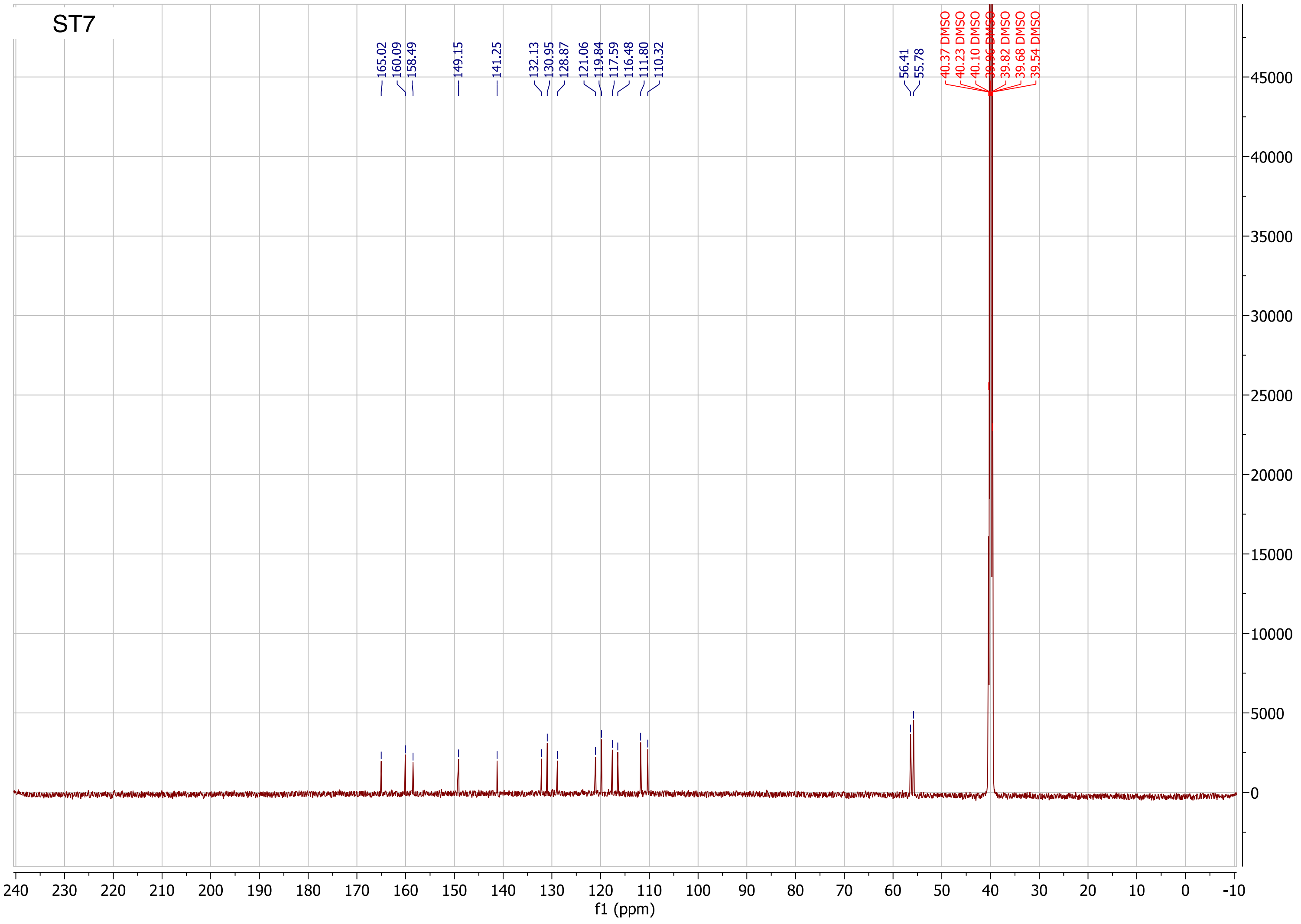

Figure S51. The ^{13}C NMR of compound ST7.

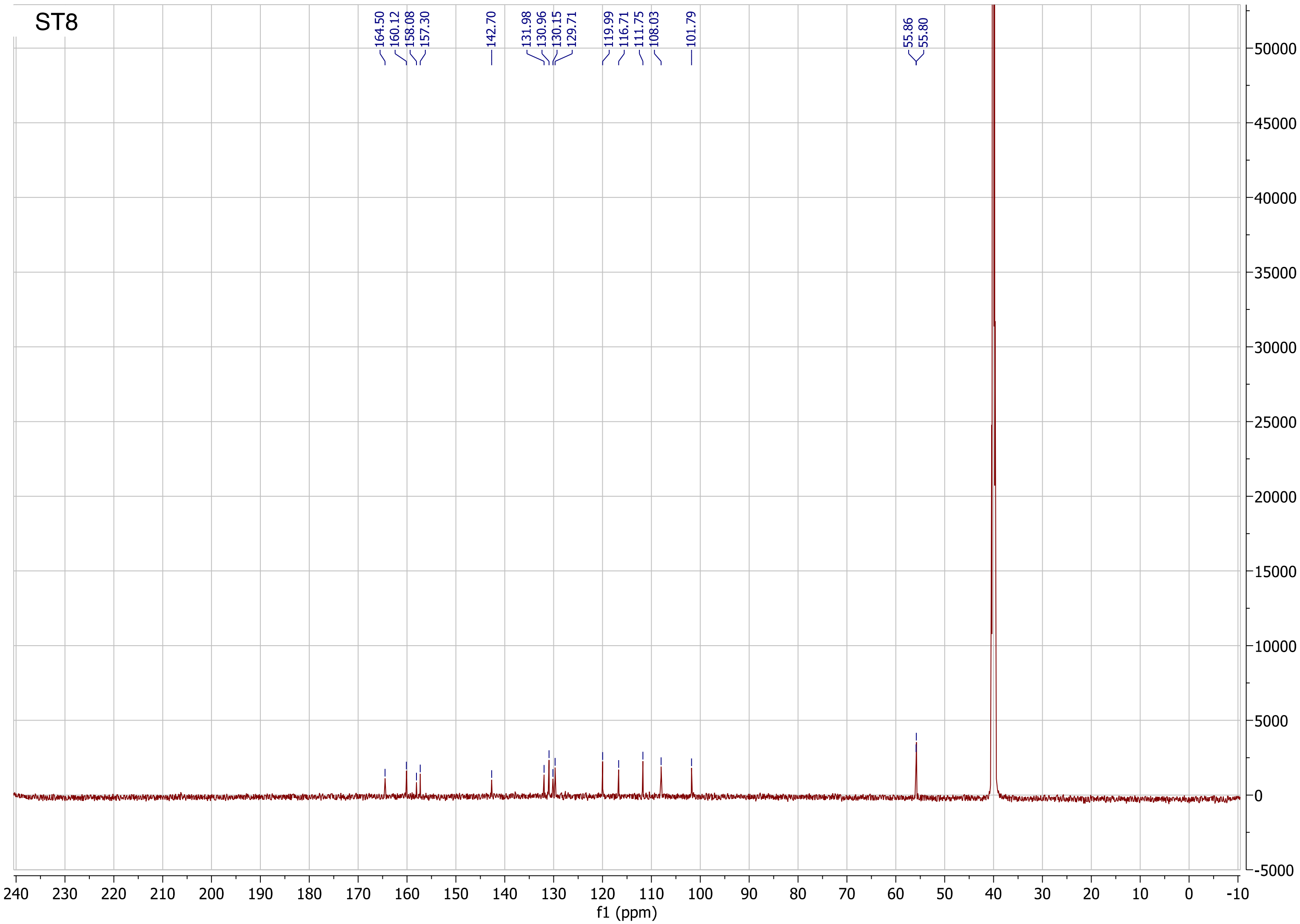

Figure S52. The ^{13}C NMR of compound ST8.

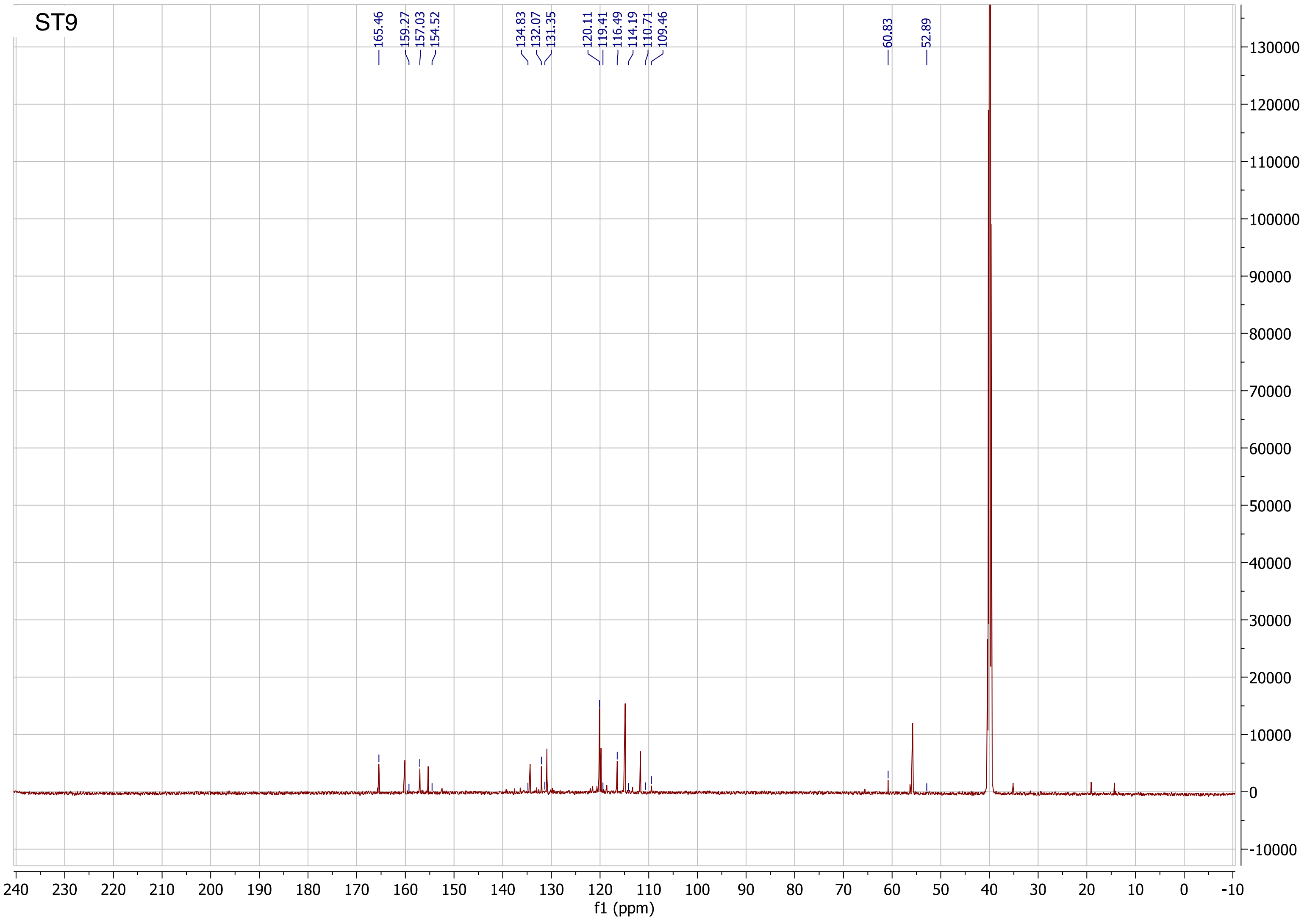

Figure S53. The ^{13}C NMR of compound ST9.

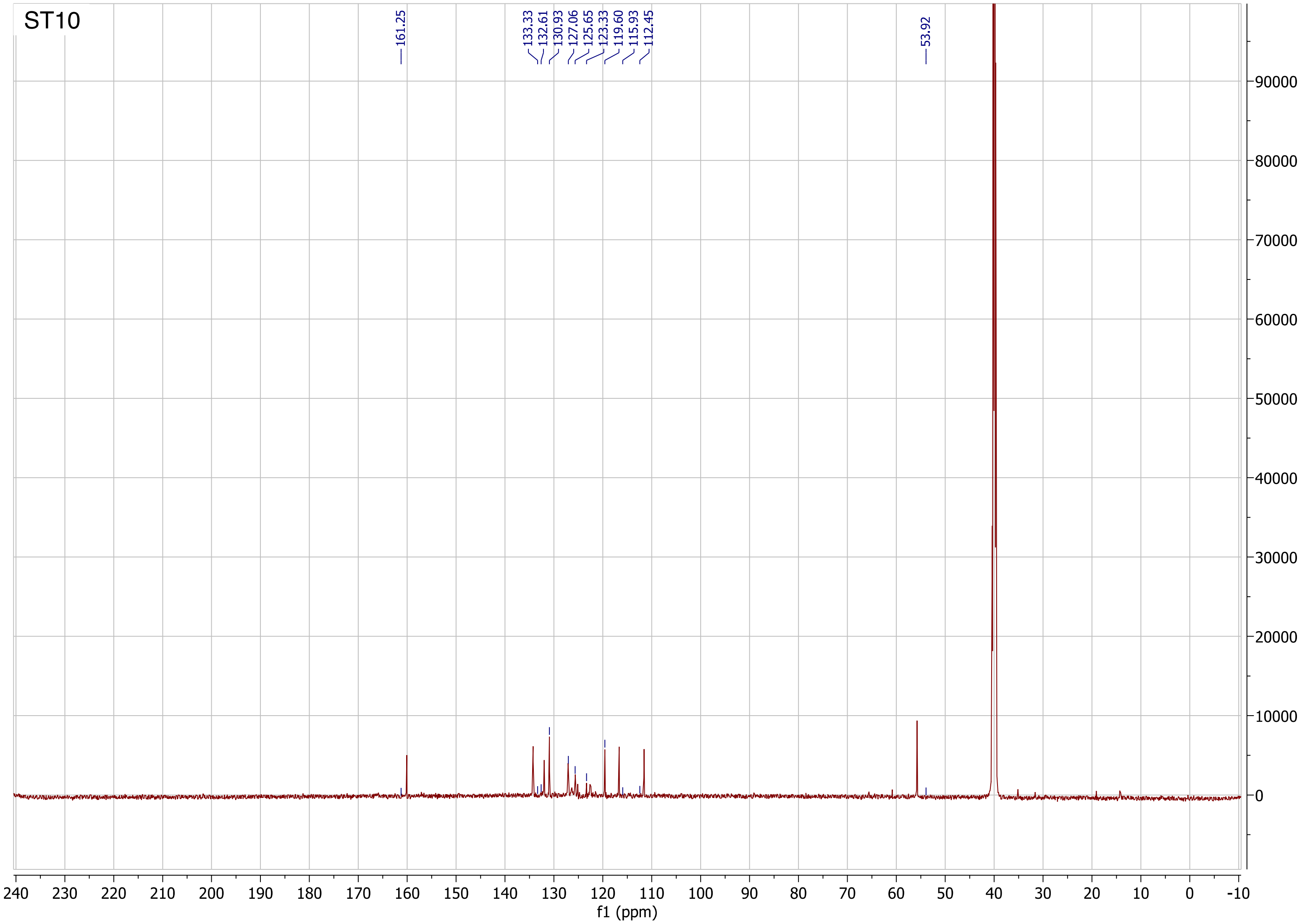

Figure S54. The ^{13}C NMR of compound ST10.

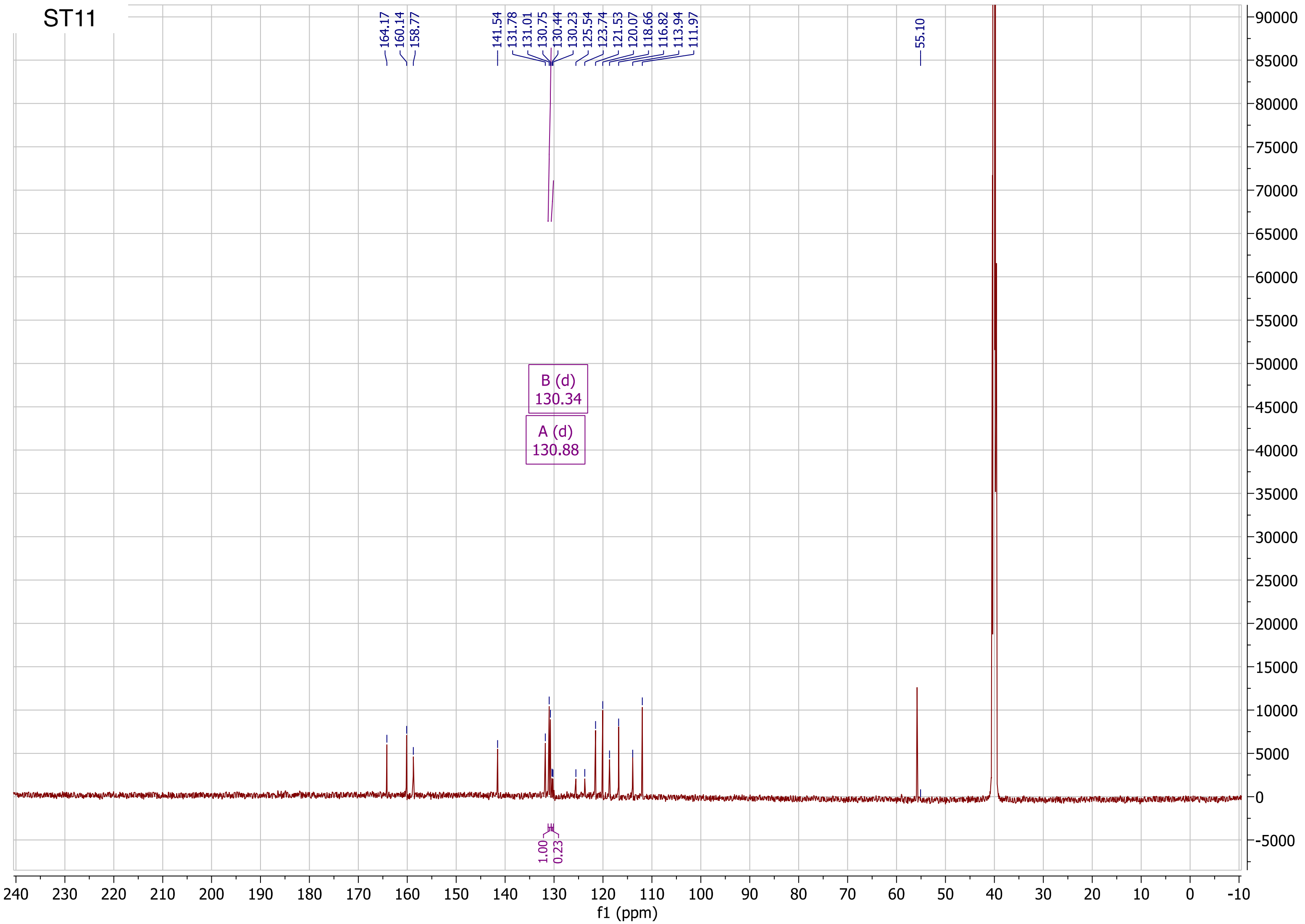

Figure S55. The ^{13}C NMR of compound ST11.

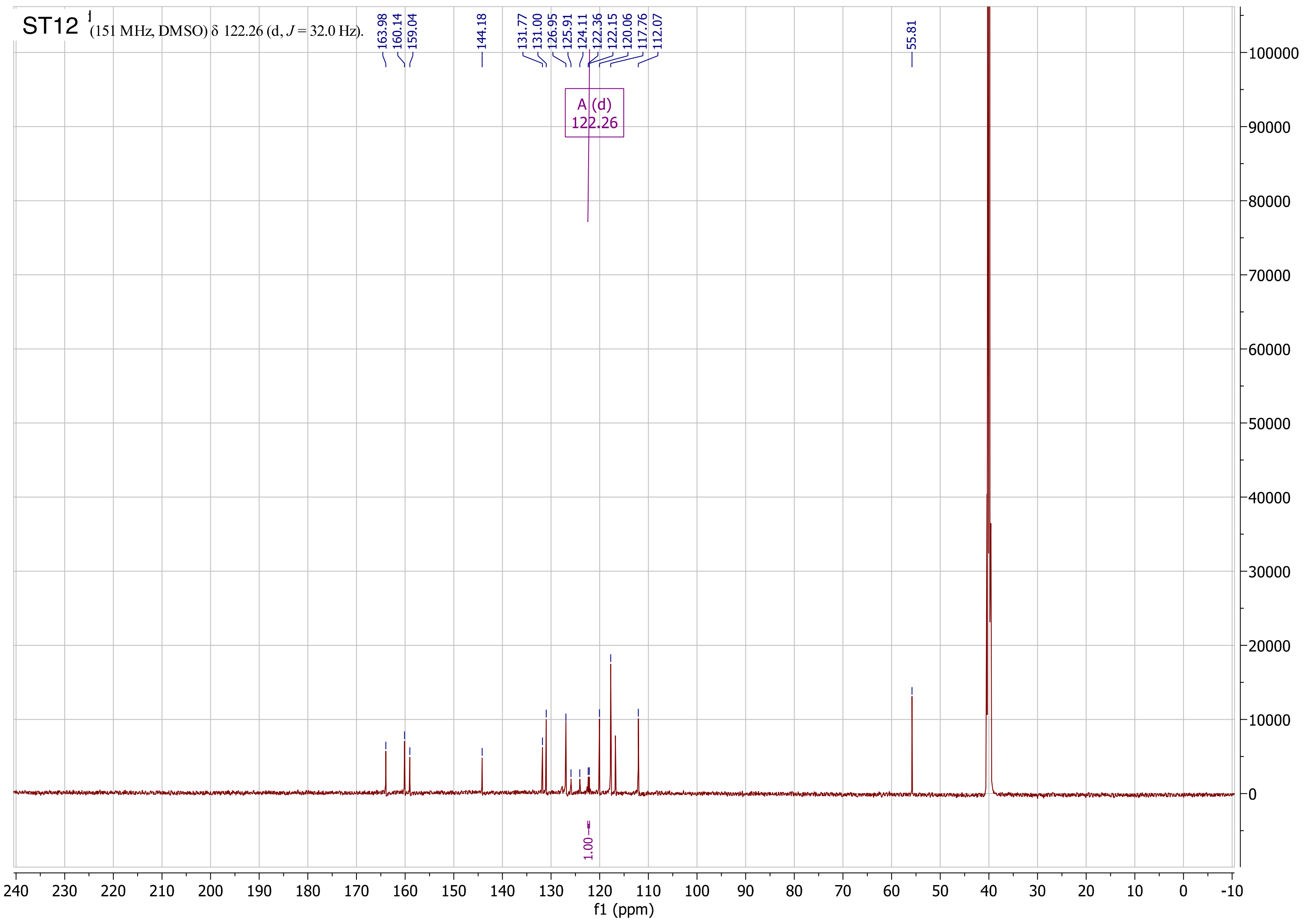

Figure S56. The ^{13}C NMR of compound ST12.

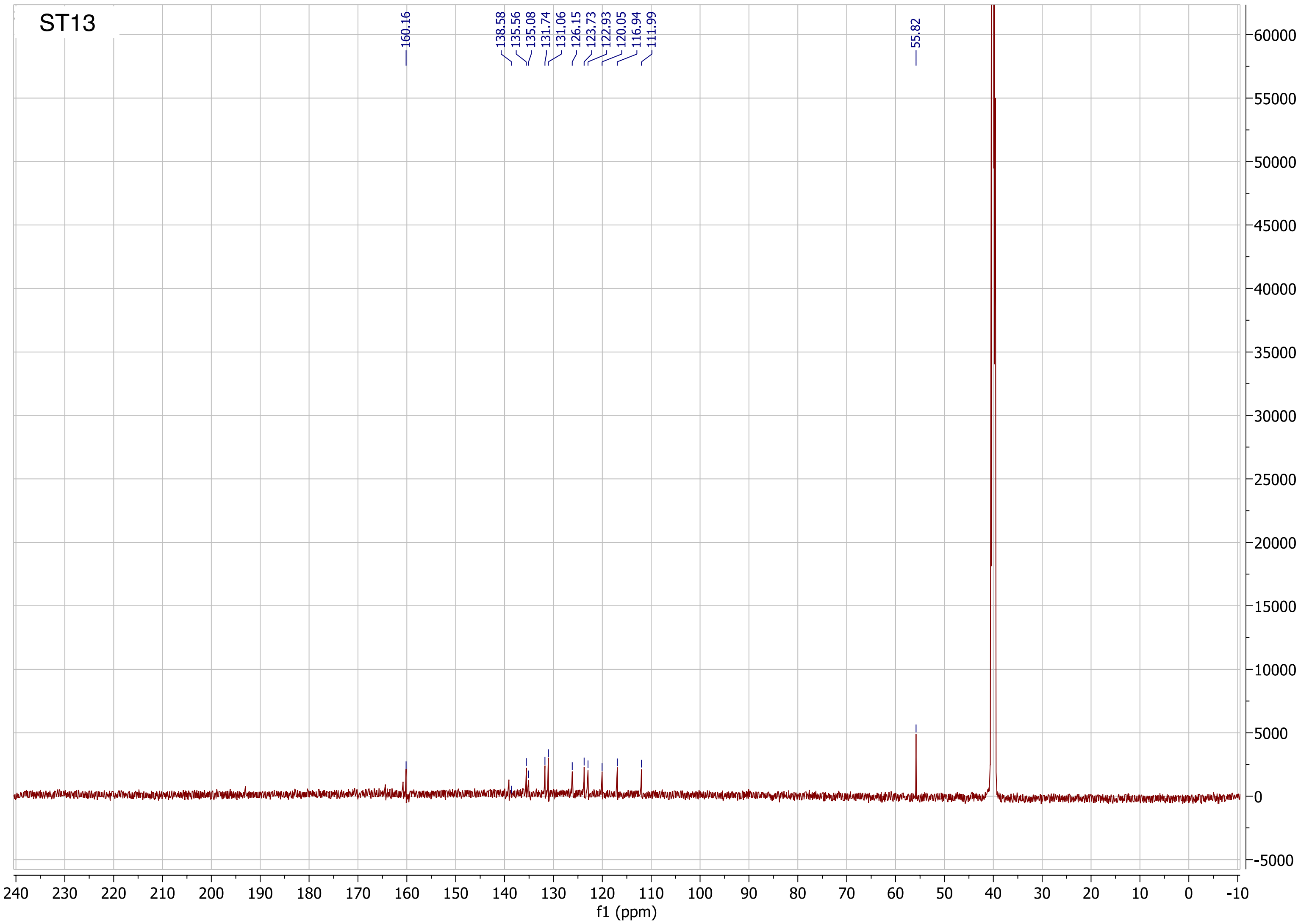

Figure S57. The ^{13}C NMR of compound ST13.

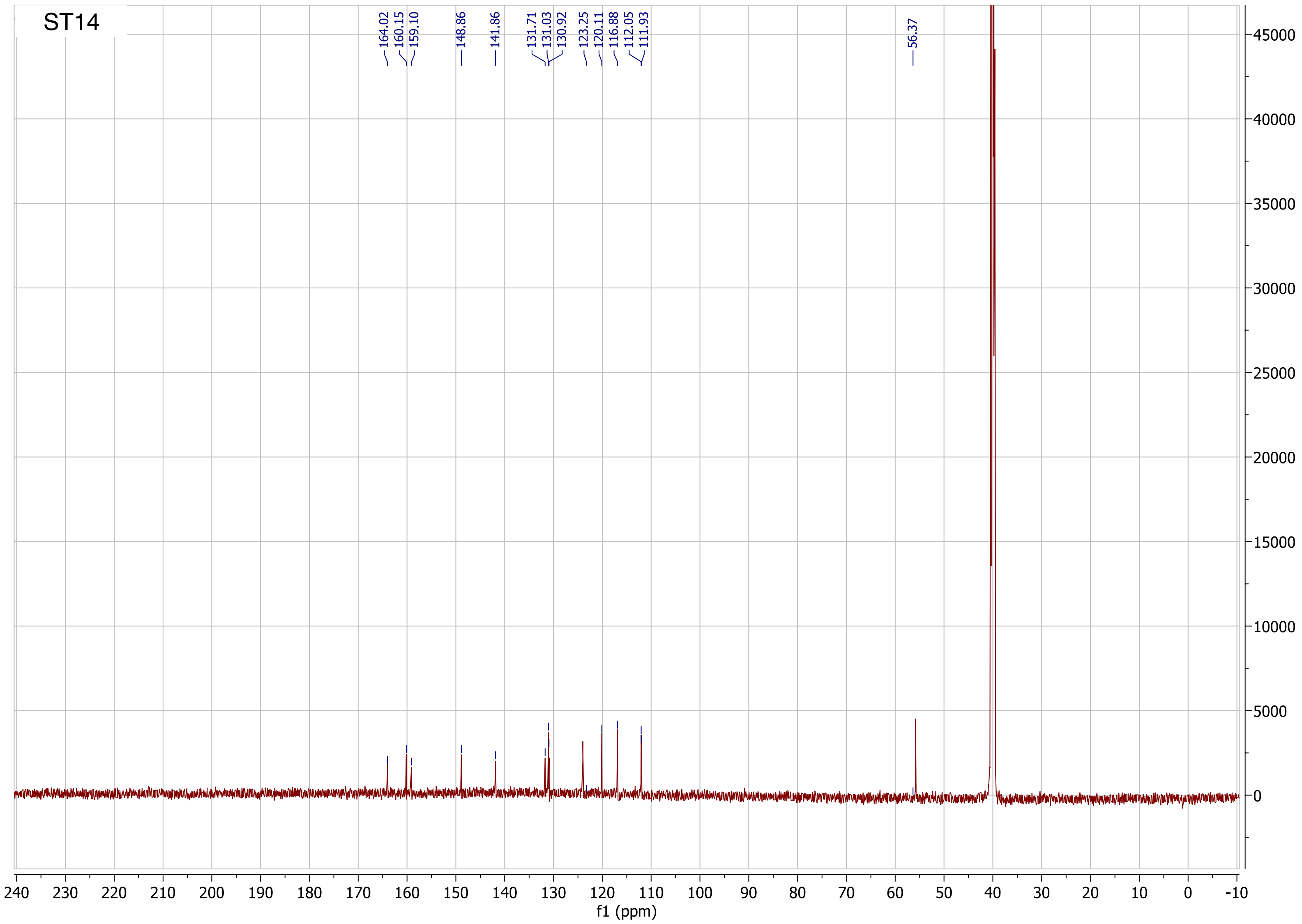

Figure S58. The ^{13}C NMR of compound ST14.

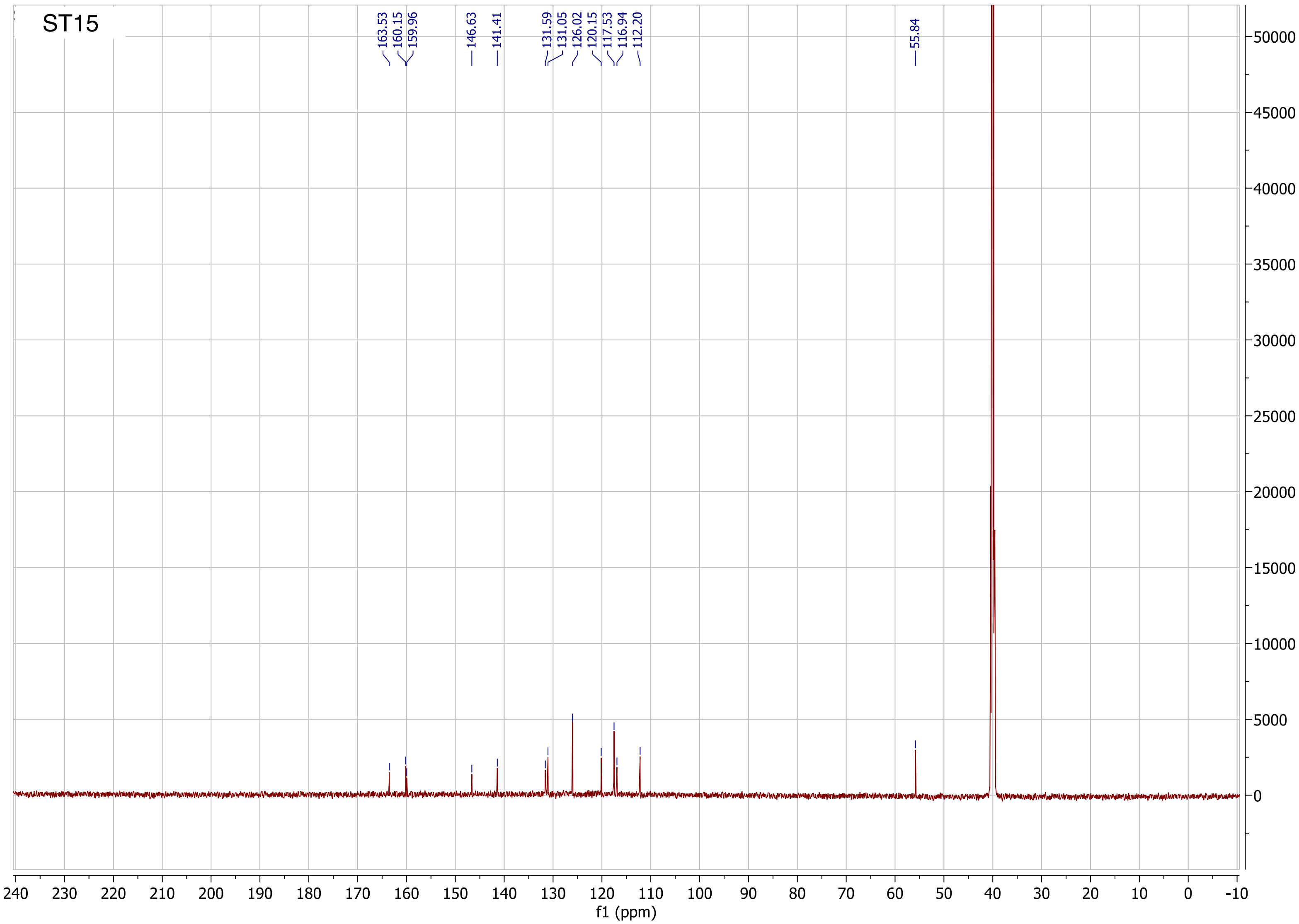

Figure S59. The ^{13}C NMR of compound ST15.
